# CellRank for directed single-cell fate mapping

**DOI:** 10.1038/s41592-021-01346-6

**Published:** 2022-01-13

**Authors:** Marius Lange, Volker Bergen, Michal Klein, Manu Setty, Bernhard Reuter, Mostafa Bakhti, Heiko Lickert, Meshal Ansari, Janine Schniering, Herbert B. Schiller, Dana Pe’er, Fabian J. Theis

**Affiliations:** 1 Institute of Computational Biology, Helmholtz Center Munich, Munich, Germany; 2 Department of Mathematics, Technical University of Munich, Munich, Germany; 3 Program for Computational and Systems Biology, Sloan Kettering Institute, Memorial Sloan Kettering Cancer Center, New York, NY USA; 4 Department of Computer Science, University of Tübingen, Tübingen, Germany; 5 Zuse Institute Berlin (ZIB), Berlin, Germany; 6 Institute of Diabetes and Regeneration Research, Helmholtz Center Munich, Munich, Germany; 7 German Center for Diabetes Research (DZD), Neuherberg, Germany; 8 Comprehensive Pneumology Center (CPC) / Institute of Lung Biology and Disease (ILBD), Helmholtz Zentrum München, Member of the German Center for Lung Research (DZL), Munich, Germany; 9 TUM School of Life Sciences Weihenstephan, Technical University of Munich, Munich, Germany; 10 Present Address: Basic Sciences Division and Translational Data Science IRC, Fred Hutchinson Cancer Research Center, Seattle WA, USA

**Keywords:** Computational models, Machine learning, Software, Statistical methods, Differentiation

## Abstract

Computational trajectory inference enables the reconstruction of cell state dynamics from single-cell RNA sequencing experiments. However, trajectory inference requires that the direction of a biological process is known, largely limiting its application to differentiating systems in normal development. Here, we present CellRank (https://cellrank.org) for single-cell fate mapping in diverse scenarios, including regeneration, reprogramming and disease, for which direction is unknown. Our approach combines the robustness of trajectory inference with directional information from RNA velocity, taking into account the gradual and stochastic nature of cellular fate decisions, as well as uncertainty in velocity vectors. On pancreas development data, CellRank automatically detects initial, intermediate and terminal populations, predicts fate potentials and visualizes continuous gene expression trends along individual lineages. Applied to lineage-traced cellular reprogramming data, predicted fate probabilities correctly recover reprogramming outcomes. CellRank also predicts a new dedifferentiation trajectory during postinjury lung regeneration, including previously unknown intermediate cell states, which we confirm experimentally.

## Abstract (web summary)

CellRank infers directed cell state transitions and cell fates incorporating RNA velocity information into a graph based Markov process.

## Main

Cells undergo state transitions during many biological processes, including development, reprogramming, regeneration and cancer, and they typically do so in a highly asynchronous fashion^1^. Single-cell RNA sequencing (scRNA-seq) successfully captures the heterogeneity that results from these processes, but it loses lineage relationships, since each cell can be measured only once. To mitigate this problem, scRNA-seq can be combined with lineage tracing methods^2,3^ that use heritable barcodes to follow clonal evolution over long time scales, or metabolic labeling methods^4–6^ that use the ratio of nascent to mature RNA molecules to link observed gene expression profiles over short time windows. Yet both strategies are mostly limited to in vitro applications, prompting the development of computational approaches to reconstruct pseudotime trajectories^1,7–12^, which leverage the observation that developmentally related cells tend to share similar gene expression profiles. Pseudotime approaches have been used extensively to order cells along differentiation trajectories and to study cell-fate decisions.

Computational trajectory inference typically demands prior biological knowledge to determine the directionality of cell state changes, often by specifying an initial cell^13^, thereby limiting its applicability to normal developmental scenarios with known cell-fate hierarchies. RNA velocity^14^ has been shown recently to alleviate this problem by reconstructing trajectory direction based on the spliced-to-unspliced mRNA ratio. The approach has been generalized to include transient cell populations and protein kinetics^15,16^; however, velocity estimates are noisy and the interpretation of high-dimensional velocity vectors has been limited mostly to low-dimensional projections, which do not easily reveal long-range probabilistic fates or allow quantitative interpretation.

Here, we present CellRank, a method that combines the robustness of similarity-based trajectory inference with directional information from RNA velocity to learn directed, probabilistic state-change trajectories under either normal or perturbed conditions. Unlike other approaches, CellRank automatically infers initial, intermediate and terminal populations of an scRNA-seq dataset and computes fate probabilities that account for the stochastic nature of cellular fate decisions as well as uncertainty in velocity estimates. We use fate probabilities to uncover putative lineage drivers and to visualize lineage-specific gene expression trends. We demonstrate CellRank’s capabilities on pancreatic endocrine lineage development, correctly recovering initial and terminal states in addition to lineage bias and key driver genes for somatostatin-producing delta cell differentiation. We show that CellRank generalizes beyond normal development by applying it to a reprogramming dataset, where predicted fate bias correctly recovers lineage-tracing-derived ground truth. Further, by applying CellRank to lung regeneration, we predict a new dedifferentiation trajectory and experimentally validate newly discovered intermediate cell states. CellRank outperforms methods that do not include velocity information, and is available as a scalable, user-friendly open-source software package with documentation and tutorials at https://cellrank.org.

## Results

### CellRank combines cell–cell similarity with RNA velocity to model cellular state transitions

**Extended Data Fig. 1:**
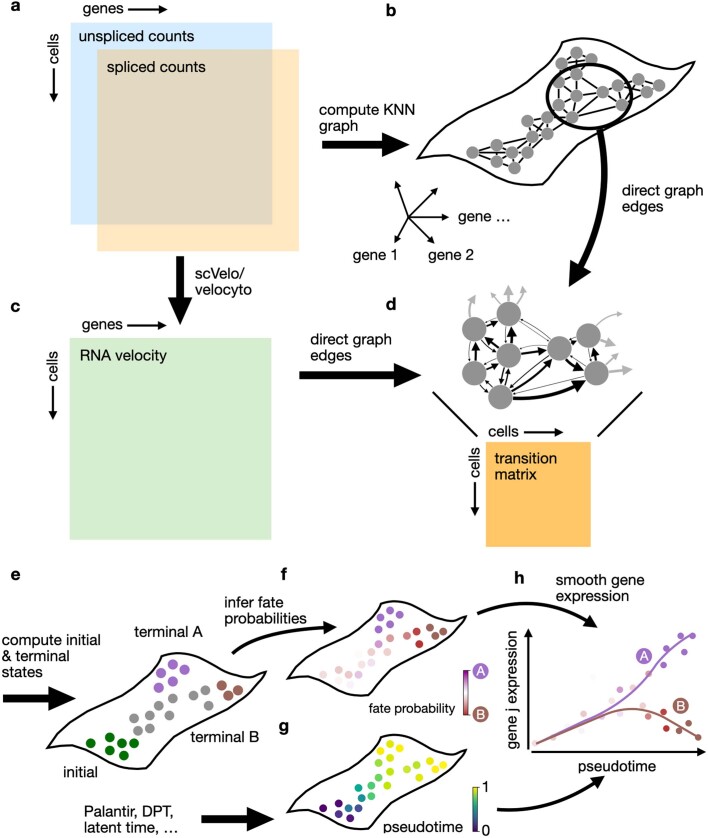
The CellRank algorithm. **a–h**. CellRank combines transcriptional similarity with velocity information to infer cell state trajectories. The algorithm starts with a pair of count matrices containing spliced and unspliced mRNA counts (**a**), computes a kNN graph in the space of principal components (**b**), uses scVelo or velocyto to compute RNA velocity vectors (**c**), and then combines the kNN graph with the velocity vectors to assign transition probabilities to graph edges (**d**). Arrow thickness indicates transition probability. A cell–cell transition matrix summarizes these probabilities. CellRank coarse-grains this matrix to compute macrostates, which it classifies as initial, intermediate or terminal (**e**), and then computes fate probabilities towards the terminal macrostates (**f**). Pseudotime is determined using an imported method such as Palantir, scVelo’s latent time or an alternative, based on macrostates defined by CellRank (**g**). The CellRank toolbox offers a number of downstream functionalities to visualize and extract information from pseudotime and cell fate probability vectors (see Methods), including the ability to combine these two quantities to chart smooth gene expression trends (**h**).

The CellRank algorithm aims to model the cell state dynamics of a system (Methods). CellRank detects the initial, terminal and intermediate cell states of the system and computes a global map of fate potentials, assigning each cell the probability of reaching each terminal state. Based on the inferred potentials, CellRank charts gene expression dynamics as cells take on different fates and identifies putative regulators of cell-fate decisions. The algorithm uses an scRNA-seq count matrix and corresponding RNA velocity matrix as input (Extended Data Figure 1a,c). Note that, while we use RNA velocity here to approximate the direction of cellular dynamics, CellRank generalizes to accommodate any vector field that provides a directional measure, such as metabolic labeling^4–6^ or real time information^17,18^.

**Fig. 1:**
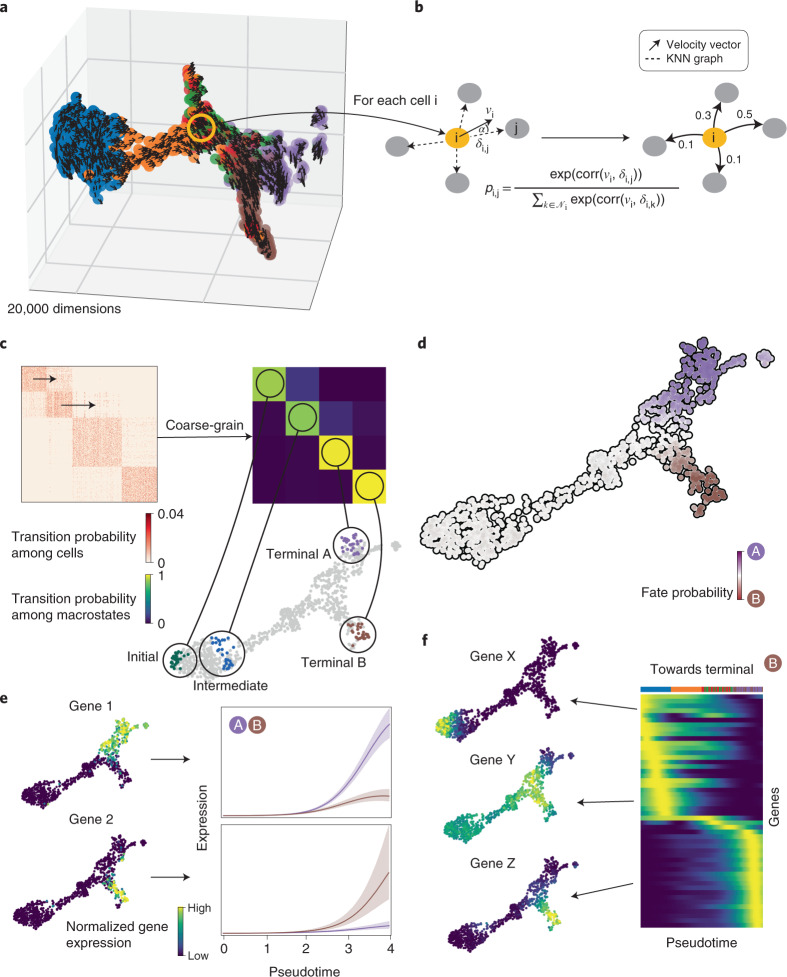
Combining RNA velocity with cell–cell similarity to determine initial and terminal states and compute a global map of cellular fate potential. **a**, 3D UMAP of 1,000 simulated cells with their velocity vectors, using DynGen^66^. Colors reflect DynGen ground truth branch assignment. CellRank models cell state transitions directly in high-dimensional gene expression space. **b**, A reference cell i with velocity vector *v*_i_ and its nearest neighbors. The vector *δ*_i,j_ is the difference in gene expression between cells j and i. To assign probability *p*_*i,j*_ to cell i transitioning to cell j in the neighborhood *N*_i_ of cell i, we transform correlations between the transcriptomic difference vectors *δ*_i,j_ and the velocity vector *v*_i_, essentially considering the angle *α* between these vectors. **c**, The directed transition matrix is coarse-grained into four macrostates. Heatmaps show transition probabilities among cells (left) and macrostates (right); sorting cells according to macrostate membership recovers block structure in the cell–cell transition matrix. We recover initial, intermediate and two terminal states. The 30 colored cells are mostly likely to belong to each macrostate in the UMAP. **d**, For each cell, we compute its probability of reaching A or B. We show these fate probabilities in a fate map, where each cell is colored according to the terminal state it is most likely to reach. Color intensity reflects the degree of lineage priming. **e**, Using these fate probabilities and a pseudotime, we plot gene expression trends, which are specific to either A or B. Left, each cell is colored based on the expression of the indicated genes; right, respective trends along pseudotime towards each fate. **f**, Expression trends in pseudotime of the top 50 genes whose expression correlates best with the probability of reaching B in a heatmap. Genes have been sorted according to their smoothed peak in pseudotime. One early gene (X), one intermediate gene (Y) and one late gene (Z) are highlighted by showing expression in the UMAP.

The main assumption underlying all pseudotime algorithms that faithfully capture trajectories^1,7–10^ is that cell states change in small steps with many transitional populations. CellRank uses the same assumption to model state transitions using a Markov chain, where each state in the chain is given by one observed cellular profile, and edge weights denote the probability of transitioning from one cell to another. The first step in chain construction is to compute an undirected K nearest neighbor (KNN) graph representing cell–cell similarities in the phenotypic manifold (Fig. 1a,b and Extended Data Fig. 1b; Methods). Each node in the graph represents an observed cellular profile, and edges connect cells that are most similar.

Unlike pseudotime algorithms, we infuse directionality by using RNA velocity to direct Markov chain edges (Extended Data Fig. 1c). The RNA velocity vector of a given cell uses splicing dynamics to predict which genes are currently being up- or downregulated, and thus points towards the likely future state of that cell. The more a neighboring cell lies in the direction of the velocity vector, the higher its transition probability (Methods). We compute a second set of transition probabilities based on gene expression similarity between cells and combine it with the first set via a weighted mean (Methods). The resulting matrix of directed transition probabilities is independent of any low-dimensional embedding and reflects transcriptional similarity as well as directional information given by RNA velocity.

**Extended Data Fig. 2:**
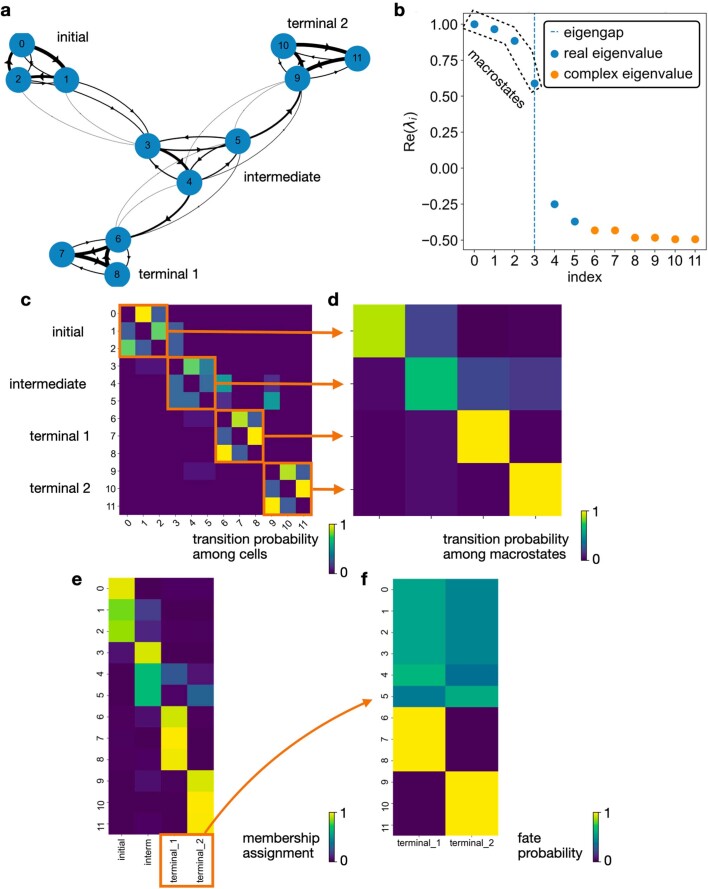
GPCCA and fate probabilities extract the essence of cellular state transitions. **a**. Markov transition graph of a toy example of cellular state changes. Starting from a cyclic initial state, cells transition via an intermediate state into either one of two terminal states, both of which are cycling again. Note that cell number 3 is more likely to go to cell number 4 than 5, which results in a global fate bias towards the first terminal state. **b**. The corresponding transition matrix can be decomposed into real Schur vectors, each corresponding to one eigenvalue. The 4 eigenvalues close to one are associated with the initial, terminal and intermediate states. Complex eigenvalues appear because the transition matrix is nonsymmetric. **c**. The original transition matrix. The block structure shows the separation into the 4 macrostates and the possible transitions between them. **d**. The coarse-grained transition matrix, identifying the different macrostates and their transition probabilities relative to one another. The initial state is the macrostate with almost no incoming but large outgoing transition probability. The intermediate state is the state with both large incoming and large outgoing transition probability, and relatively little self-transition probability. The terminal states are the states with large incoming, but almost no outgoing and large self-transition probability. **e**. Each macrostate is associated with a membership vector that assigns cells to the state in a soft fashion, that is using weights that sum to one. We show the 4 membership vectors in a heatmap. **f**. Fate probabilities towards the two terminal states. We correctly recover the global bias towards the first terminal state.

The transition matrix may be extremely large, noisy and difficult to interpret. We alleviate these problems by summarizing individual gene expression profiles into macrostates, regions of the phenotypic manifold that cells are unlikely to leave (Fig. 1c and Extended Data Fig. 2a–e). CellRank decomposes the dynamics of the Markov chain into these macrostates and computes coarse-grained transition probabilities among them. The number of macrostates is a model parameter that can be chosen using knee-point heuristics or previous knowledge about the biological system (Extended Data Fig. 2b; Methods). Individual cells are assigned to macrostates via a soft assignment. To compute macrostates and the induced coarse-grained transition probabilities, we adapt Generalized Perron Cluster Cluster Analysis (GPCCA)^19,20^ to the single-cell context (Methods).

Viewing the biological system at coarse resolution allows us to identify populations based on transition probabilities: terminal macrostates will have high self-transition probability, initial macrostates will have low incoming transition probability, and remaining macrostates will be intermediate. We automate the identification of terminal states through a stability index (SI) between zero and one, indicating self-transition probability; macrostates with an SI of 0.96 or greater are classified as terminal. We automate the identification of initial states through the coarse-grained stationary distribution (CGSD), which describes the long-term evolution of the coarse-grained Markov chain (Methods). The CGSD assigns small values to macrostates that the process is unlikely to revisit after leaving; these macrostates are classified as initial. The number of initial states is a parameter that is set to one by default.

Finally, CellRank uses the directed single-cell transition matrix to compute fate probability, the likelihood that a given cell will ultimately transition towards each terminal population defined in the previous step (Fig. 1d and Extended Data Fig. 2f). These probabilities can be efficiently computed for all cells by solving a linear system (Methods). Fate probabilities extend the short-range fate prediction given by RNA velocity to the global structure spanning initial to terminal states. The stochastic Markov chain-based formulation allows us to overcome noise in individual velocity vectors and cell–cell similarities by aggregating many of these into our final fate prediction. Moreover, by restricting transitions to be within the phenotypic manifold, CellRank captures cell state dynamics more faithfully.

**Extended Data Fig. 3:**
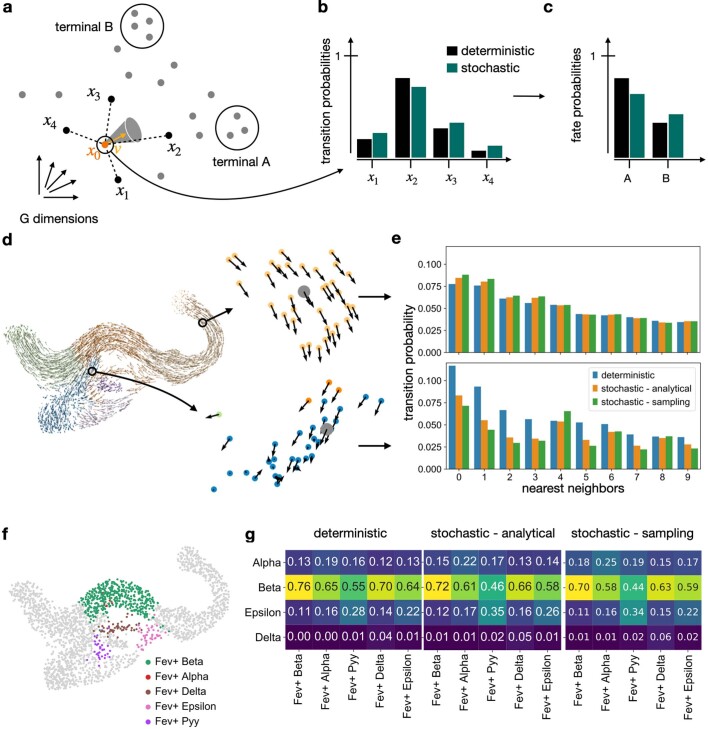
Uncertainty propagation adjusts for noise in RNA velocity vectors. **a**. When predicting the future state of cell *x*_0_, CellRank takes uncertainty in the velocity vector *v* in the high-dimensional gene space into account. **b**. Propagating noise changes the transition probabilities from one cell to its nearest neighbors. **c**. The adjusted transition probabilities agglomerate over longer paths to result in adjusted fate probabilities. **d**. Effect of noise propagation, illustrated using pancreas data. One cell from a low noise region, where velocity vectors from neighboring cells tend to point in the same direction (top), and one from a high noise region, where vectors from neighboring cells point in different directions (bottom), are highlighted. **e**. Transition probabilities from the reference cell to its 10 nearest neighbors using a deterministic or stochastic (analytical approximation or Monte Carlo sampling-based) formulation, for both the low and high noise cell. Corrections applied by stochastic approaches are larger in the high noise region. **f**. Subclustering of the Fev+ cluster in the pancreas data^24^. **g**. Comparing average fate probabilities per subcluster. These were obtained from not propagating (‘deterministic’) or propagating (‘stochastic – analytical’ and ‘stochastic – sampling’) velocity uncertainty. Both stochastic approaches agree in down-weighting probability towards the dominant beta fate and up-weighting probability towards the alpha, delta and epsilon fates.

Both the original velocyto and generalized scVelo models compute velocity vectors on the basis of spliced-to-unspliced count ratios^14,15^. These counts are influenced by many sources of biological and technical noise, such as ambient RNA, sparsity, doublets, bursting kinetics and low capture efficiency. Unspliced RNA in particular is rarer in the cell and suffers from low detection rates. The uncertainty in molecule counts translates into uncertainty in RNA velocity vectors, which can be estimated in scVelo (Extended Data Fig. 3a; Methods). CellRank accounts for these sources of uncertainty by propagating the estimated distribution over velocity vectors (Extended Data Fig. 3b,c). By default, it uses an analytical approximation that computes the expected value of the transition probabilities towards nearest neighbors, given the distribution over velocity vectors (Methods). The analytical approximation is very efficient and ensures that uncertainty can be estimated even for large datasets. Alternatively, CellRank has an option for far slower, more accurate computation of fate probabilities via Monte Carlo (MC) sampling (Methods).

We combine fate probability estimates with a pseudotemporal ordering to visualize gene expression programs executed by cells along trajectories leading to terminal states (Fig. 1e and Extended Data Fig. 1e–h; Methods). Pseudotime orders a progression of cell states from the initial state, while CellRank fate probabilities indicate how committed each cell is to every trajectory. By softly assigning cells to trajectories via fate probabilities, we capture the effect of gradual lineage commitment, whereby cells transition from an uncommitted state (contribution to several trajectories) to a committed state (contribution to a single trajectory)^21–23^. Palantir^21^, which is based on an iteratively refined shortest path in the space of diffusion components, is used for pseudotime ordering by default, where Palantir is provided with CellRank’s computed initial state. By correlating gene expression with fate probabilities, CellRank enhances the ability to uncover putative trajectory-specific regulators (Fig. 1f). By sorting putative regulators according to their peak in pseudotime, we visualize gene expression cascades specific to their cellular trajectory while accounting for the continuous nature of cellular fate commitment.

### CellRank recapitulates coarse-state dynamics of pancreatic endocrine lineage formation

**Fig. 2:**
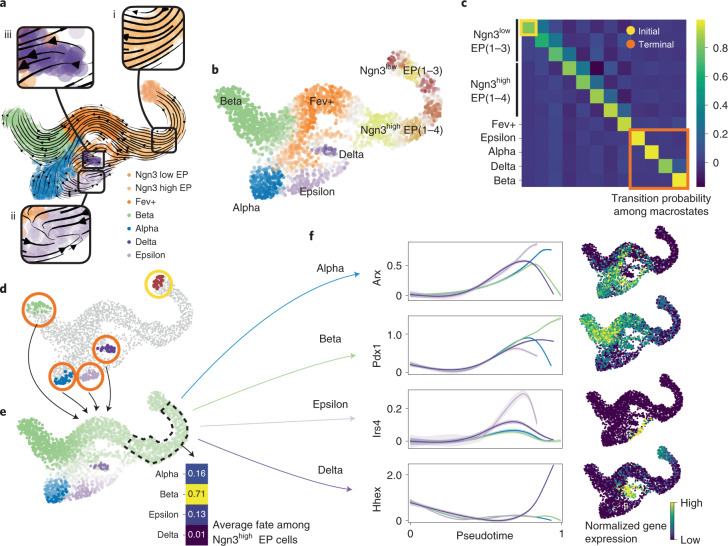
Delineating fate choice in pancreatic development. **a**, UMAP of murine pancreatic development at E15.5 with scVelo-projected velocities, shown as streamlines. Colors correspond to published cluster annotations^24^. CellRank provides further insights regarding (i) the fate of early cells, (ii) the identification of terminal states and (iii) likely progenitors of terminal populations (boxed insets). **b**, Soft assignment of cells to macrostates. Cells colored by most probable macrostate; color intensity reflects degree of confidence, and gray cells reside between several macrostates. **c**, Coarse-grained transition probabilities among macrostates. Terminal macrostates are outlined in red and the initial Ngn3^low^ EP_1 macrostate is outlined in yellow. **d**, Highlight of the 30 cells most confidently assigned to each initial and terminal macrostate, colored as in **b**. **e**, UMAP showing probabilities for reaching alpha, beta, epsilon and delta terminal fates. Fates colored as in **b**, with darker color indicating higher probability. Inset shows average fate probabilities of cells in the Ngn3^high^ EP cluster marked with a dashed line. **f**, Smoothed gene expression trends in pseudotime, each colored trend is weighted by CellRank fate probabilities as indicated for the lineage determinants Arx^33^ (alpha), Pdx1 (ref. ^34^) (beta) and Hhex^35^ (delta) as well as the lineage-associated gene Irs4 (ref. ^36^) (epsilon). The trend for each gene is shown for each trajectory leading to the indicated terminal population. Right column, expression values for the corresponding gene in the UMAP.

We applied CellRank to an scRNA-seq dataset of E15.5 murine pancreatic development^24^. A UMAP^25^ representation with original cluster annotations and scVelo-projected velocities recapitulated the main developmental trends^15^ (Fig. 2a); from an initial cluster of endocrine progenitors (EPs) expressing low levels of the transcription factor neurogenin 3 (*Neurog3* or *Ngn3*), cells traverse trajectories towards alpha, beta, epsilon and delta cell fates.

To investigate specific questions, such as the onset of lineage bias, precise location of initial and terminal states and probable progenitors of any terminal state, we argue against basing hypotheses purely on the projected velocity vectors, for three reasons. First, projecting onto only two or three dimensions may over-regularize the true velocities and lead to overly smooth vector fields. Interpreting cellular trajectories in two-dimensional (2D) or three-dimensional embeddings is often misleading, as high-dimensional distances cannot be fully preserved in lower dimensions; this is why most neighborhood-based dimensionality reduction techniques such as t-distributed stochastic neighbor embedding (t-SNE)^26,27^ and uniform manifold approximation and projection (UMAP)^28^ do not conserve global relationships well^29–31^. Second, visual interpretation of projected vectors ignores uncertainty in RNA velocity and therefore leads to overconfidence in the inferred trajectories. Third, velocities are available only locally, whereas CellRank aggregates these local signals globally, computing longer range trends. The single-cell field has reached a consensus that clustering cells in 2D or 3D representations must be avoided^32^ and, similarly, we argue that velocity vectors projected onto two or three dimensions must not be used to address detailed questions of trajectory inference. CellRank overcomes these limitations and allows us to model global trajectories, as we demonstrate on pancreas data below.

**Extended Data Fig. 4:**
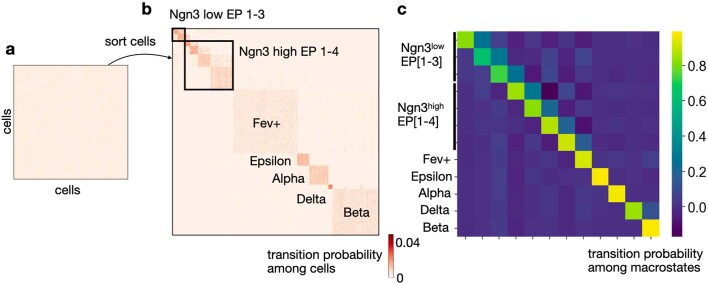
Recovering structure in the transition matrix. **a**. Heatmap of the transition matrix for the pancreas dataset from Fig. 2a. The ordering of cells (rows and columns) in the matrix is arbitrary. The colorbar has been adjusted such that values larger than the 90th percentile are clipped to the 90th percentile to avoid skewing the colorbar towards extreme values. However, there is still no visible structure in the matrix because of sparsity, noise and the random order of cells. **b**. Same matrix as in (**a**), just re-ordered such that cells which likely belong to the same macrostate are next to each other. This recovers the structure of the developmental dynamics. Note that the sparsity structure of the matrix is symmetric (KNN graph is symmetric) while the actual values are not (RNA velocity infused directionality). **c**. Coarse-grained transition matrix from Fig. 2. Macrostates defined in this matrix were used to reorder cells in (**b**).

We computed CellRank’s directed transition matrix, and then coarse-grained it into 12 macrostates (Fig. 2b) based on eigenvalue gap analysis (Supplementary Fig. 1a,b), revealing a block-like structure in the transition matrix (Fig. 2c and Extended Data Fig. 4a–c). Macrostates, annotated according to their overlap with the underlying gene expression clusters (Methods), comprised all developmental stages in this dataset, from an initial Ngn3^low^ EP state, to intermediate Ngn3^high^ EP and Fev+ states, to terminal hormone-producing alpha, beta, epsilon and delta cell states.

**Extended Data Fig. 5:**
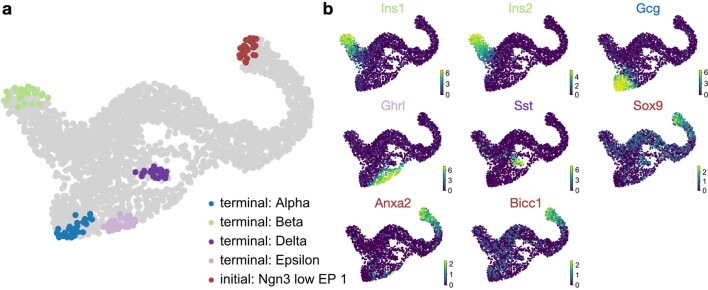
Marker genes confirm CellRanks initial and terminal state annotations in the pancreas data. **a**. CellRank-computed initial and terminal states from Fig. 2d. **b**. Cells are colored based on the expression level of the indicated gene in each UMAP. The terminal states express the key marker genes relevant for each respective cell type. Showing for beta: *Ins1* and *Ins2* (insulin), alpha: *Gcg* (glucagon), epsilon: *Ghrl* (ghrelin), delta: *Sst* (somatostatin)^33^. For the initial state, we show expression of ductal cell markers *Sox9*, *Anxa2* and *Bicc1*^24,33^.

The three most stable states according to the coarse-grained transition matrix were alpha (SI 0.97), beta (SI 1.00) and epsilon (SI 0.98) macrostates, which were accordingly labeled as terminal by CellRank, consistent with known biology (Fig. 2d). We recovered one relatively stable (SI 0.84) macrostate that largely overlapped with delta cells. We identified the Ngn3^low^ EP_1 state as initial because it was assigned the smallest CGSD value (2 × 10^−6^). The initial and terminal states agree with the expression of well-known marker genes, including *Ins1* and *Ins2* for beta, *Gcg* for alpha, *Ghrl* for epsilon, *Sst* for delta cells and ductal cell markers *Sox9*, *Anxa2* and *Bicc1* for the initial state^24,33^ (Extended Data Fig. 5a,b).

We computed fate probabilities and summarized them in a fate map (Fig. 2e). This analysis correctly identified the beta cell fate as dominant in the Ngn3^high^ EP cluster at E15.5, consistent with known biology^24^ (Fig. 2e, inset), as also visualized with pie charts on a directed implementation of partition-based graph abstraction^8^ (PAGA) (Supplementary Fig. 3; Methods). Using a cell in the Ngn3^low^ EP_1 macrostate as the starting state for Palantir^21^, we ordered cells in pseudotime (Supplementary Fig. 4) and overlaid the expression of master regulators *Arx*^33^ (alpha), *Pdx1*^34^ (beta) and *Hhex*^35^ (delta), and the lineage-associated gene *Irs4*^36^ (epsilon) (Fig. 2f) to visualize trends based on CellRank’s fate probabilities. All of these genes were upregulated correctly when approaching their associated terminal populations.

All components of CellRank are extremely robust to parameter variation, based on sensitivity analysis for the number of macrostates (Supplementary Fig. 5), weight given to transcriptomic similarities, number of neighbors in the KNN graph, scVelo minimal gene counts, number of highly variable genes and number of principal components (PCs). CellRank is robust to random subsampling of cells (Supplementary Figs. 6 and 7).

We used the pancreas dataset to investigate the effects of uncertainty propagation (Extended Data Fig. 3d). We selected two cells, one from a low noise region where velocity vectors of neighboring cells tend to agree and one from a high noise region. To compute transition probabilities towards nearest neighbors, we used a deterministic approach that does not propagate uncertainty, as well as our analytical approximation and MC sampling. Differences between deterministic and stochastic transition probabilities were greatest in the high noise region, highlighting that uncertainty propagation automatically downweights transitions towards cells in noisy areas where individual velocity vectors are less trustworthy (Extended Data Fig. 3e). We confirmed that our analytical approximation and the asymptotically exact sampling scheme give similar results (Extended Data Fig. 3f,g). Overall, propagating uncertainty leads to increased robustness of fate probabilities (Supplementary Figs. 2, 8 and 9).

**Extended Data Fig. 6:**
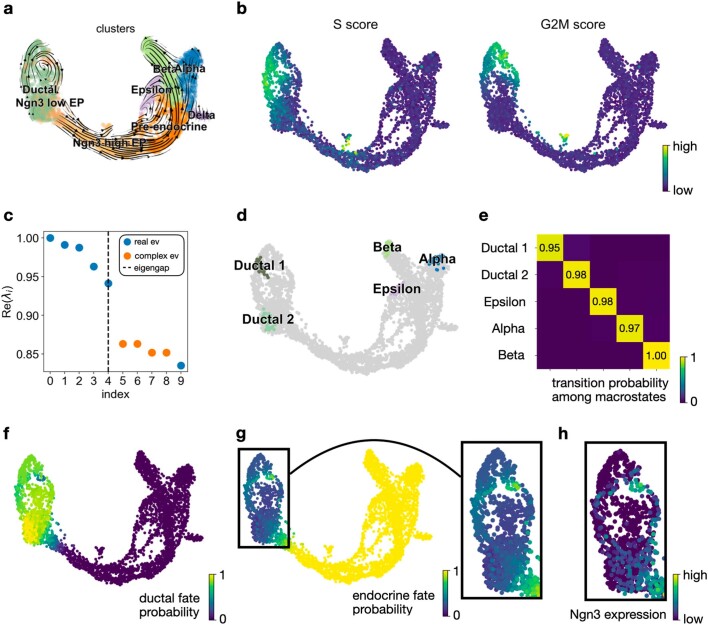
CellRank recovers ductal and endocrine maturation. **a**. Published^15^ UMAP of the pancreas data^24^, colored by original cluster annotations, including all clusters in Fig. 2 as well as ductal cells. Streamlines show scVelo^15^ velocities. **b**. Cell cycle scores for S and G2M phase, computed using scanpy^76^ based on the gene set from ref. ^107^. Cycling cells mainly map to the ductal cluster. **c**. Spectrum of the CellRank-computed transition matrix. An eigengap appears after the first 5 eigenvalues, suggestive of 5 macrostates. **d**. UMAP showing 5 macrostates. **e**. Coarse-grained transition matrix. Diagonal elements give the stability index (SI) values. These automatically identify the Ductal 2, Epsilon, Alpha and Beta macrostates to be terminal. **f**. Fate probabilities towards the Ductal 2 state (‘ductal fate probability’). Among the 25 genes that correlate best with ductal fate probability in the ductal/Ngn3^low^ clusters is *Sox9*, an established marker for ductal cell maturation^33^. **g**. Combined fate probabilities towards the Alpha, Beta and Epsilon macrostates (‘endocrine fate probability’). Among the 10 genes that correlate best with endocrine fate probability in the ductal/Ngn3^low^ clusters are *Ngn3* and *Insm1*, which are established endocrine progenitor markers^33^. Inset shows endocrine fate probabilities in the ductal/Ngn3^low^ clusters. **h**. *Ngn3* expression for the subset of cells shown in (**g**). *Ngn3* expression correlates best with endocrine fate probabilities (Pearson correlation = 0.66).

To evaluate whether CellRank can overcome situations in which the signals of differentiation and proliferation are confounded, we included a population of cycling ductal cells (Extended Data Fig. 6a,b). Coarse-grained transition probabilities among five macrostates automatically identified ductal and endocrine terminal states (Extended Data Fig. 6c–e), and fate probabilities towards the ductal and endocrine lineages correlated well with known lineage markers (Extended Data Fig. 6f–h).

### CellRank identifies putative gene programs driving delta cell differentiation

**Extended Data Fig. 7:**
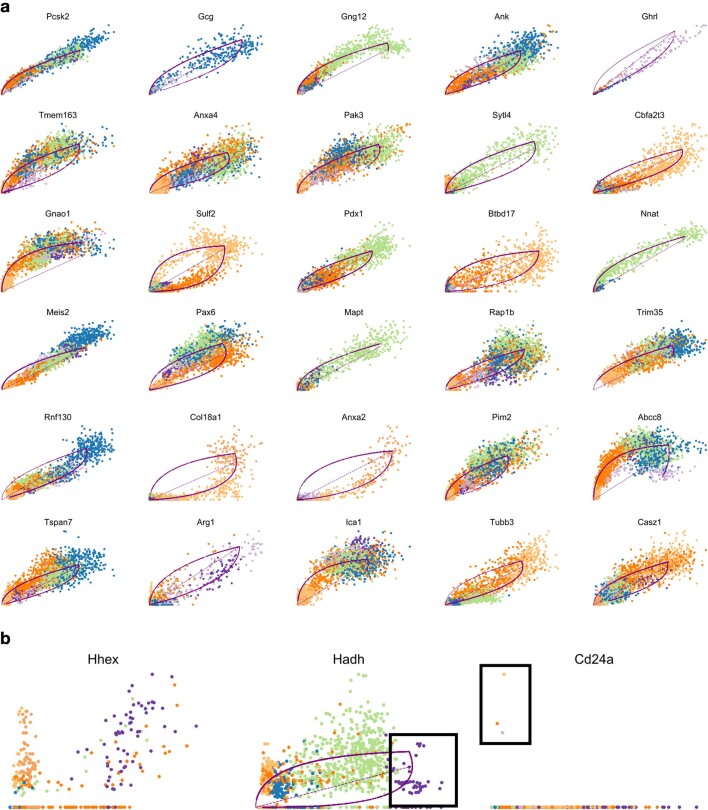
Splicing kinetics do not capture delta cell development. **a**. Phase portraits of the top 30 genes which are assigned the highest likelihoods by scVelo’s dynamical model of the mRNA lifecycle. Unspliced counts are on the x-axis, spliced counts are on the y-axis. Cells are colored according to the clusters from Fig. 2a. The solid purple curve is scVelo’s dynamical fit and the dashed purple line is scVelo’s inferred steady-state ratio. The top 30 genes are dominated by drivers for the alpha (*Gcg*^33^), epsilon (*Ghrl*^33^) and beta (*Gng12*^108^*, Pdx1*^34,70^) lineages while delta drivers are not present. **b**. Phase portraits of known delta-associated genes *Hhex*^35^ *and Cd24a*^38,39^ as well as the CellRank-identified putative lineage driver *Hadh. Cd24a* was filtered out by scVelo’s gene filtering threshold because it only had unspliced counts in 3 cells (see box). *Hhex* could not be fit by scVelo because of too little expression and too large noise levels. *Hadh* could be fit by scVelo, however, delta cells are an outlier in this fit (see box) and were not correctly assigned to the steady-state. Thus, all three genes do not meaningfully contribute to velocity vectors towards delta cells.

Delta cells highlight how CellRank’s global approach overcomes limitations in RNA velocity. Delta cells are very rare in our data (70 cells or 3% of total; Supplementary Fig. 10) and, more importantly, no known drivers of delta cell development were among the 30 scVelo genes with highest likelihoods (Extended Data Fig. 7a). Moreover, genes implicated in delta cell development were not captured well by scVelo’s model of splicing kinetics (Extended Data Fig. 7b). We hypothesize that splicing kinetics fail to capture delta cell differentiation because these cells appear late in pancreatic development and thus are very rare in our data^37^.

**Fig. 3:**
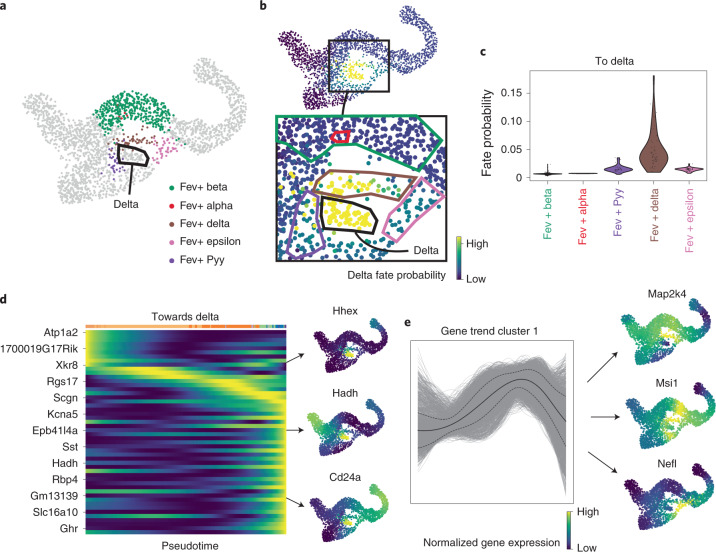
Zooming into the delta state to explain differentiation paths. **a**, Orthogonal subclustering of Fev+ hormone-negative endocrine cells from Bastidas-Ponce et al.^24^. Delta cells are indicated by the black outline. **b**, CellRank probabilities for acquiring the terminal delta cell fate. Cells are colored by the probability of reaching the delta state. Inset, group of cells likely to become delta, showing differentiation path predicted by CellRank. Fev+ subclusters are marked by outlines, colored as in **a**. Delta cells are marked with a black outline. **c**, CellRank probabilities in each Fev+ subcluster. Note that this does not include the delta cluster itself. Cells in the Fev+ delta subcluster are assigned significantly higher probability by CellRank (two-sided Welch’s *t*-test, 51 Fev+ Delta cells versus 536 other Fev+ cells, *t* = 8.6, *P* = 1.7 × 10^−11^; Methods). **d**, Smoothed gene expression trends of the top 50 genes whose expression values correlate best with delta fate probabilities, sorted according to peak in pseudotime. Not all gene names are shown (see Supplementary Fig. 11 for full list). Right, UMAP projected MAGIC^67^ imputed expression values of *Hhex* and *Cd24a*, examples of known regulators that were identified automatically, as well as of *Hadh*. **e**, Smoothed gene expression trends along the delta lineage for all 12,987 genes that are expressed in at least ten cells, clustered using louvain^68^. Cluster 1 contains transiently upregulated genes. The solid line denotes mean trend, dashed lines denote 1 s.d. Genes in cluster 1 are sorted according to their correlation with delta fate probabilities. Right, expression on the UMAP of *Map2k4*, *Msi1* and *Nefl*, among the genes that correlated best.

**Extended Data Fig. 8:**
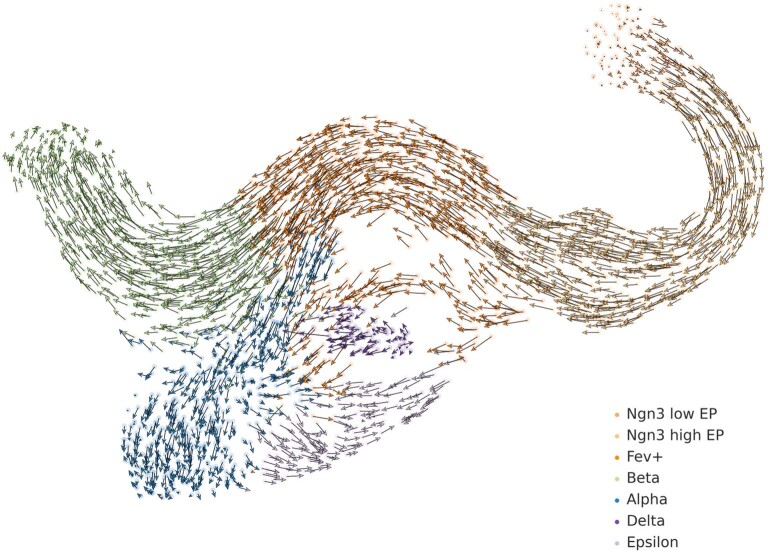
Projected velocities do not reveal how delta cells are generated. scVelo velocities projected onto the UMAP do not reveal a likely path towards delta cells. Velocities reveal short-range fate relationships but cannot be combined to give long-range fate predictions from looking at an embedding.

The development of delta cells is not well understood^33^. Mature delta cells can be identified by *Sst* expression (Extended Data Fig. 5), but immature cells are much more difficult to identify. *Hhex* is the only widely accepted transcription factor required to maintain delta cell differentiation, and specifically marks delta cells in the adult islets of Langerhans^35^, and *Cd24a* has recently been implicated in human delta cell development^38,39^. To learn more about delta cell development, we focused on CellRank fate probabilities towards the relatively stable delta macrostate (SI 0.84), which was not automatically classified as terminal^33^ (Fig. 3a,b). Velocities projected onto the UMAP do not disclose likely delta cell precursors (Extended Data Fig. 8), but CellRank fate probabilities show one path with highest likelihood, through cells that were annotated as delta precursors in a study^24^ involving subclustering of the Fev+ population (Fig. 3c). Therefore, while RNA velocity fails to capture the dynamics of delta cell development, they can be recovered successfully by CellRank because it constrains velocities to the phenotypic manifold via the KNN graph, incorporates cell–cell similarly and models long-range trends.

To discover more delta genes, we correlated gene expression values in the Fev+ cluster against CellRank delta fate probabilities (Methods). Smoothed gene expression trends for the 50 genes with highest correlation showed a cascade of gene activation events (Fig. 3d and Supplementary Fig. 11). Among the top 50 genes are *Hhex* and *Cd24*, as well as *Sst*, the hormone produced by mature delta cells^33^. Genes with no previously described role in delta cell differentiation include *Hadh* (a target of *Foxa2*, implicated in pancreatic differentiation^40^), *Isl1* (a transcription factor involved in pancreatic differentiation^41^) and *Pkhd1* (a target of *Hnf1a/b*^42^, transcription factors involved in pancreatic differentiation^43^). Next, we focused on a cluster of transiently upregulated genes (Fig. 3e). When ranked by their correlation with delta fate, we identified *Map2k4*, *Msi1* and *Nefl* as new candidate regulators. *Msi1* is regulated by *Rfx4* (ref. ^44^), which is a paralog of *Rfx6* that is structurally related to *Rfx3* (ref. ^45^), both of which are involved in endocrine differentiation^46,47^.

### Lineage tracing supports fate probabilities in reprogramming

**Fig. 4:**
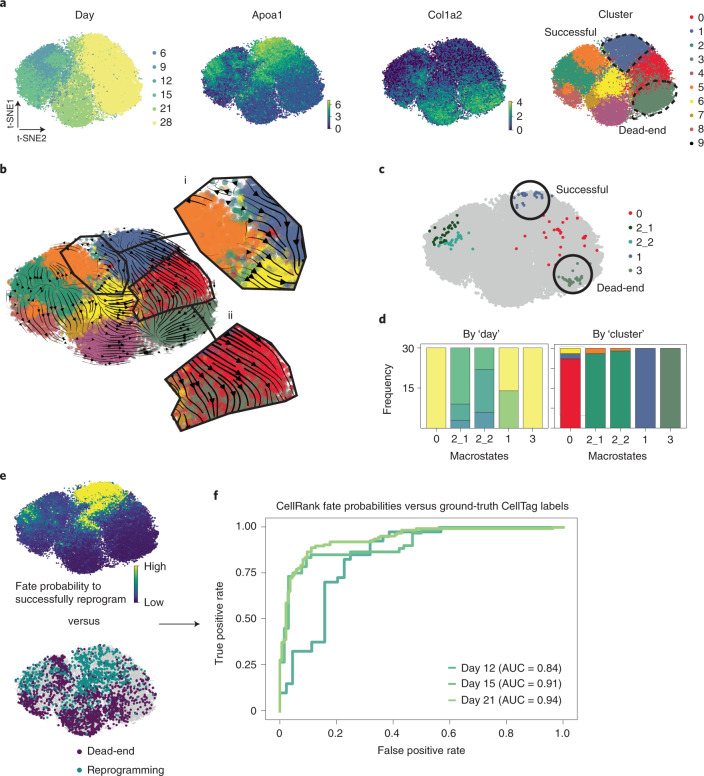
CellRank predicts reprogramming outcome. **a**, t-SNE^26,27^ embeddings of 48,515 MEFs reprogramming to iEPs^48,69^. Each dot represents a cell, colored by day after reprogramming induction by retroviral overexpression of *Foxa1* and *Hnf4a*^49^; expression of iEP marker *Apoa1*, indicative of reprogramming success; expression of MEF marker *Col1a2*, indicative of reprogramming failure; or original cluster annotations from Biddy et al.^49^. Clusters 1 and 3 (dashed lines) represent successful and dead-end states, respectively^49^. **b**, scVelo^15^ velocities, projected on the t-SNE embedding in **a** and shown as streamlines. Velocities do not disclose a route towards successful reprogramming (i) and falsely show a transition from successful to dead-end states (ii). **c**, CellRank-computed macrostates, colored by cluster from **a** that they mostly overlap with. **d**, Distribution over reprogramming day (left) and cluster (right), colored by the same labels as in **a**. Macrostates 1 and 3 contain only late-stage cells (days 21 and 28) from clusters 1 and 3, respectively, thus representing the successful and dead-end states. **e**, CellRank’s fate probabilities towards the successful macrostate 1 (top) and ground truth labels from CellTagging^49^ lineage tracing (bottom). For 3,049 cells across all days in the time course, these labels show the likely reprogramming outcome^49^. **f**, AUC of CellRank fate probabilities at days 12, 15 and 21, based on classifiers trained to predict reprogramming outcome using CellTag labels (**e**) as ground truth (Methods).

The pancreas example demonstrated how CellRank can be used to study differentiation trajectories during normal development. Moving to a perturbation scenario, we applied CellRank to a dataset of 48,515 mouse embryonic fibroblasts (MEFs) reprogramming towards induced endoderm progenitors^48^ (iEPs) across six timepoints^49^ (Methods). Only around 1% of cells are expected to reprogram successfully (marked by *Apoa1*), while the other cells enter a ‘dead-end’ state (marked by *Col1a2*)^49^ (Fig. 4a). This dataset contains CellTagging lineage tracing information that can be used to reconstruct clonal relationships across cells, thus providing ground truth on the ultimate fate (successful versus dead-end) of early cells^49^. We were interested to see how well CellRank’s fate probabilities recovered ground truth in this challenging setting.

We computed velocities using scVelo^15^ and projected them on the original t-SNE embedding of Biddy et al.^49^ (Fig. 4b). Projected velocities failed to show a path towards the successful state, most likely because the reprogramming signal is too weak to be picked up in such low dimensions. CellRank’s macrostates, in contrast, included both a dead-end and the rare successful state (Fig. 4c,d). By computing fate probabilities towards these states and comparing them with lineage-tracing derived labels (Fig. 4e; Methods), we found that fate probabilities were highly predictive of reprogramming outcome and that predictive accuracy decreased for earlier days in the time course, as expected (Fig. 4f).

### CellRank outperforms competing methods

**Fig. 5:**
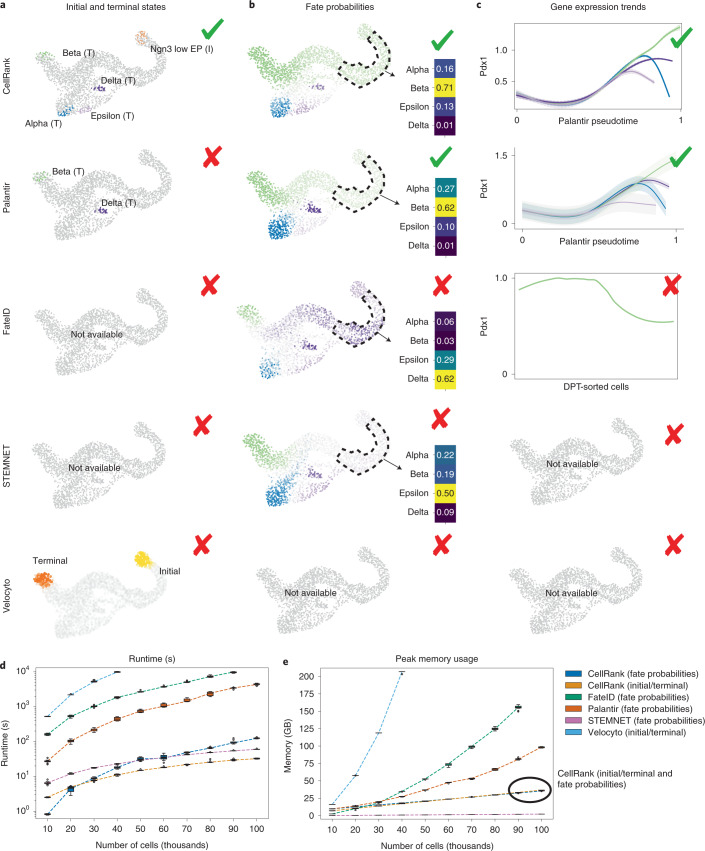
CellRank outperforms other cell-fate inference methods. **a**–**c**, Methods were compared on pancreas data. CellRank automatically identified the terminal alpha, beta and epsilon states as well as the initial Ngn3^low^ EP state (**a**). Delta cells were picked up as a macrostate and given the terminal label manually. Palantir^21^ identified terminal beta and delta states. The velocyto^14^ state distribution is depicted as overlapping with beta (terminal, orange) and Ngn3^low^ EP (initial, yellow) cells, respectively (Supplementary Note 2). Only CellRank and Palantir correctly predict beta to be the dominant fate among Ngn3^high^ EP cells (**b**). Velocyto does not compute fate probabilities. Gene expression trends for the beta-regulator *Pdx1*^34,70,71^ (**c**). On the *x* axis is the pseudotime used by the corresponding method, on the *y* axis is gene expression. For FateID^51^, the *x* axis is given by the cell indices, which are assigned to the beta lineage, sorted by DPT^7^. We show one smoothed trend per lineage for CellRank and Palantir, and a smoothed trend along just the beta lineage for FateID because it does not allow one gene to be visualized simultaneously along several lineages. CellRank and Palantir correctly identify upregulation of *Pdx1* along the beta lineage. FateID fails to do so while STEMNET^50^ and velocyto do not offer options to visualize lineage-specific gene expression trends (Supplementary Note 2). **d**,**e**, Boxplots comparing methods in terms of computational runtime (**d**) and peak memory usage (**e**) on a 100,000 cell reprogramming dataset^49^ (Supplementary Note 2). We split the datasets into ten subsets of increasing size and ran each method ten times on each subset. Boxes cover 25% to 75% quantiles, line indicates median, whiskers extend to 1.5× the interquartile range, and dots represent outliers; dashed lines connect the medians. CellRank’s peak memory usages for initial/terminal states and fate probabilities are very similar and thus the lines overlap.

To evaluate the impact of including velocity information, we benchmarked CellRank with similarity-based methods that provide cell-fate probabilities (Palantir^21^, STEMNET^50^ and FateID^51^) and a velocity-based method that computes initial/terminal states (velocyto^14^) on the pancreas data (Supplementary Note 2). Only CellRank correctly identified both initial and terminal states (Fig. 5a). Palantir requires user-provided initial states and identified only two out of four terminal states, and STEMNET and FateID cannot determine either initial or terminal states. Velocyto cannot identify individual initial or terminal states, but outputs distributions for initial and terminal states which only overlap with beta and Ngn3^low^ EP cells, respectively. Next, we supplied all methods with CellRank’s terminal states and tested cell-fate probabilities, finding that only CellRank and Palantir correctly identified beta as the dominant fate among Ngn3^high^ EP cells (Fig. 5b). Velocyto does not provide fate probabilities. For lineage-specific gene expression, CellRank and Palantir correctly predicted trends for key lineage drivers, whereas FateID failed to predict (transient) upregulation of *Pdx1* and *Pax4* along the beta lineage^33^ as well as upregulation of *Arx* along the alpha lineage^33^. STEMNET and velocyto do not provide expression trends (Fig. 5c and Supplementary Figs. 12–14).

We also benchmarked runtime and memory usage on an scRNA-seq dataset of 100,000 cells reprogramming from MEFs to iEPs^49^ (Fig. 5d and Supplementary Note 2). It took CellRank about 33 s to compute macrostates from this large dataset (Supplementary Table 1). For fate probabilities, the (generalized) linear model STEMNET was fastest as expected, taking only 1 min, while CellRank took about 2 min and Palantir took 1 h 12 min. FateID on 90,000 cells took even longer and failed on 100,000 cells due to memory constraints, whereas velocyto was the slowest, exceeding our time budget of 10,000 s for cell numbers exceeding 40,000. Memory usage results looked similar, with CellRank requiring three- and five-times less peak memory than Palantir and FateID, respectively, to compute fate probabilities on 100,000 cells (Fig. 5e and Supplementary Table 2). Only STEMNET required even less memory. Velocyto was most memory-hungry, requiring more memory on 40,000 cells than any other method on 100,000 cells. On 100,000 cells without parallelization, CellRank had a peak memory usage of less than 15 GiB, making it possible to run such large cell numbers on a laptop (Supplementary Table 3).

### Fate probabilities predict a new dedifferentiation trajectory in lung regeneration

**Extended Data Fig. 9:**
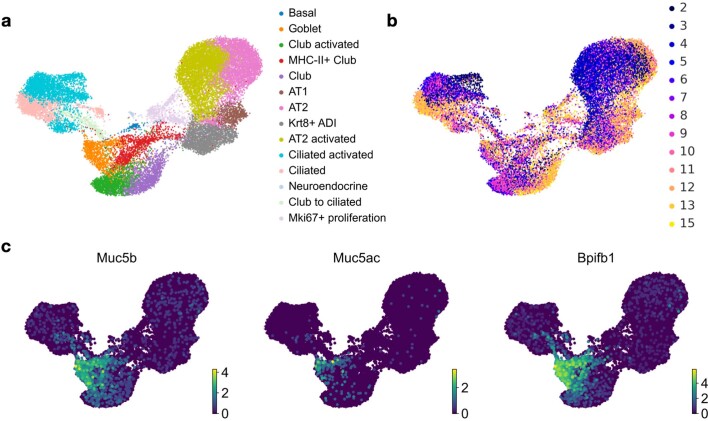
Cluster labels and timepoint annotations for lung data. **a**. Original cluster labels for the lung regeneration data^52^ in a UMAP projection. The data contains 24,882 murine lung epithelial cells sequenced using the Drop-seq workflow^53^ at 13 timepoints spanning days 2–15 past bleomycin injury. The ‘activated’ label refers to cell states that emerge after bleomycin injury. **b**. Same as (**a**) with timepoints colored in. Time points refer to time passed since bleomycin injury. **c**. Expression of goblet cell markers *Muc5b, Muc5ac and Bpifb1* agrees with the goblet annotation of (**a**).

To demonstrate CellRank’s ability in the context of regeneration, where the typical assumption of unidirectional transition to more differentiated states does not hold, we applied it to murine lung regeneration in response to acute injury^52^. The scRNA-seq dataset comprised 24,882 lung airway and alveolar epithelial cells, sequenced at 13 timepoints spanning days 2–15 after bleomycin injury (Extended Data Fig. 9a,b) with Drop-seq^53^, a lower resolution single-cell platform. A high degree of plasticity between epithelial cell types has been observed when homeostasis is perturbed and the tissue environment changes, including injury-induced reprogramming of differentiated cell types to bona fide long-lived stem cells in the lung^54^ and other organs^55^. In the current airway cell lineage model, multipotent basal cells give rise to club cells, which in turn can give rise to secretory goblet and ciliated cells^56^. It has been shown that upon ablation of basal stem cells, luminal secretory cells can dedifferentiate into fully functional basal stem cells^54^. Here, we applied CellRank for unbiased discovery of unexpected regeneration trajectories among airway cells.

**Fig. 6:**
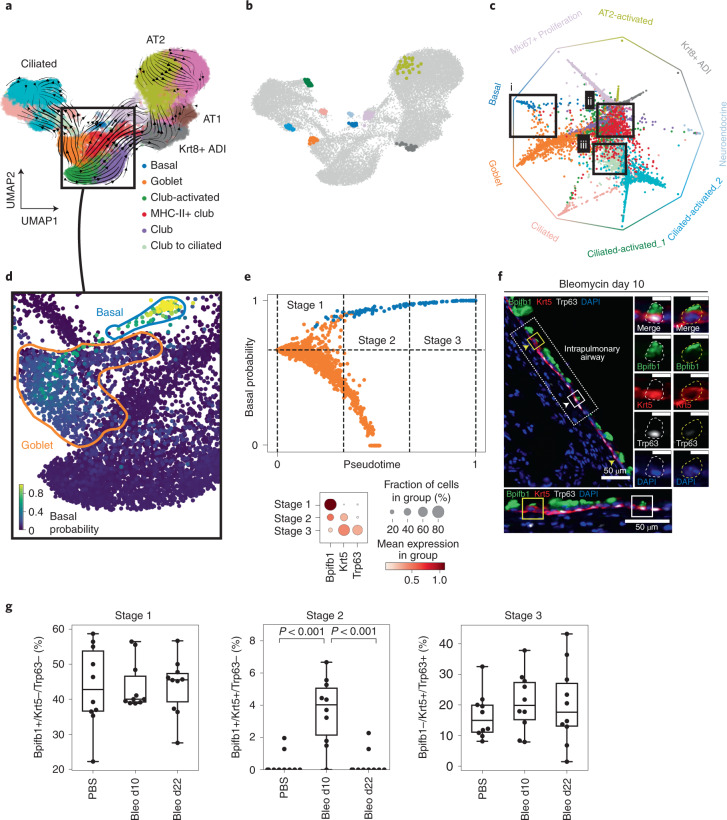
CellRank predicts a new dedifferentiation trajectory in murine lung regeneration. **a**, UMAP of 24,882 epithelial cells from 13 timepoints, spanning days 2–15 after lung injury by bleomycin treatment in mice colored according to original cluster annotations^52^. Streamlines show averaged and projected scVelo velocities and the box highlights a subset of airway cells. **b**, CellRank-computed macrostates, showing the 30 most confidently assigned cells for each state. Names and colors were assigned to macrostates according to the clusters from **a** that they overlapped most with. **c**, Circular projection^50,72^ of cells according to fate probabilities towards the macrostates shown in **b**, colored by cluster annotations from **a**. Macrostates are arranged on the edge of a circle; each cell is placed inside the circle according to its probability of reaching any of the terminal states (Methods). Cells in the center have higher multilineage potential, whereas cells closer to one of the corners are committed. Boxes highlight goblet cells likely to reach the basal terminal state (i), MHC-II+ club cells with high multilineage potential (ii) and club cells likely to transition to ciliated cells (iii). **d**, Cells in UMAP colored by CellRank-computed fate probabilities towards the basal cell macrostate, showing a route from goblet to basal cells. **e**, CellRank fate probabilities and Palantir pseudotime^21^ are used to define three stages of the dedifferentiation trajectory (top, Methods). Dedifferentiation stages are characterized by expression of *Bpifb1* (goblet), *Krt5* (early basal) and *Trp63* (late basal); stage 1 corresponds to goblet, stage 2 to intermediate and stage 3 to basal cells (bottom). **f**, Immunofluorescence stainings for Bpifb1 (green), Krt5 (red), Trp63 (white) and 4′,6-diamidino-2-phenylindole (blue) in mouse lung tissue sections 10 days after bleomycin injury. We find cells from the intermediate stage 2 (Bpfib1+/Krt5+/Trp63–) in bleomycin-injured lungs (yellow squares and arrow heads). Scale bars, 50 μm; 10 μm for enlarged images. In each panel, dotted-line boxes are magnified at the bottom, and solid-line boxed cells are magnified to the right, showing individual and merged channels. Representative images are derived from two independent biological replicates. **g**, Quantification of cell abundances by stage in wild type (PBS), 10 days after bleomycin injury (bleo d10) and 22 days postinjury (bleo d22) mice. Ten independent pulmonary airway regions per condition examined over two biologically independent experiments were quantified. Bleo d10 is significantly enriched for stage 2 cells (nested one-way analysis of variance with Tukey’s multiple comparison test, *P* < 10^−3^).

**Extended Data Fig. 10:**
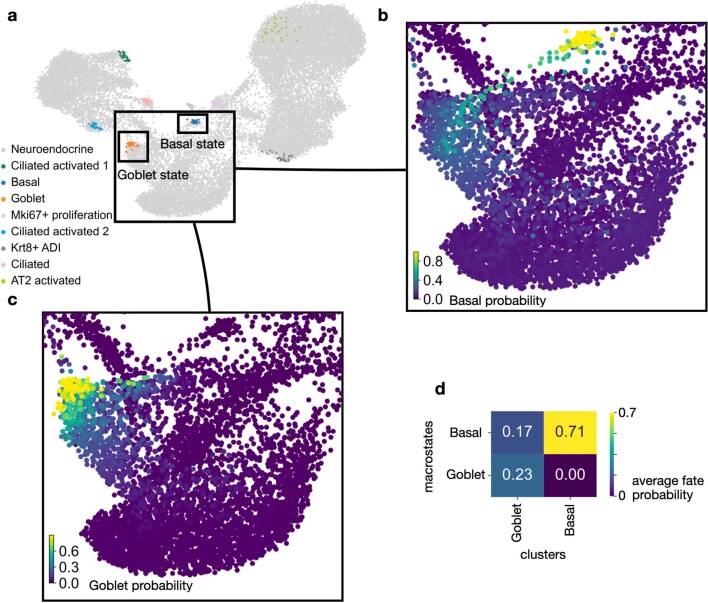
CellRank predicts a goblet to basal dedifferentiation trajectory. **a**. Cellrank identifies 9 macrostates. Airway cells, including club, goblet and basal cells, are highlighted. **b**. Single-cell fate probabilities of transitioning towards the basal state. A ‘band’ of cells within the goblet cluster exhibits high basal probability. **c**. Single-cell fate probabilities of transitioning towards the goblet state. Basal cells do not show any probability of transitioning towards the goblet state. **d**. Quantification of the results from (**b**) and (**c**). Goblet cells have a large probability of transitioning towards basal cells, but basal cells have no probability of transitioning towards the goblet state, confirming that the recovered trajectory proceeds from goblet to basal.

We computed scVelo velocities, applied CellRank and identified nine macrostates that were used to compute fate probabilities (Fig. 6a,b). Fate probabilities assigned high multilineage potential to MHC-II+ club cells, as previously reported^52^ (Fig. 6c). Focusing our analysis on airway cells, we identified three macrostates in ciliated cells, one in basal cells and one in goblet cells. In agreement with lineage tracing experiments^57^, we observed a high probability for club cells to give rise to ciliated cells (Fig. 6c). The goblet cell macrostate was distinguished from club cells by the expression of specific mucin genes such as *Muc5b* and *Muc5ac*, as well as secreted proteins involved in innate immunity, such as *Bpifb1* (Extended Data Fig. 9c). Analysis of fate probabilities towards basal and goblet states showed that goblet cells are likely to dedifferentiate towards Krt5+/Trp63+ basal cells (Fig. 6c,d and Extended Data Fig. 10).

We computed a diffusion map on basal and goblet cells alone to study the trajectory at higher resolution (Supplementary Fig. 15a). We confirmed that the fraction of basal cells increases over time and that gene-wise velocities support the dedifferentiation hypothesis (Supplementary Fig. 15b,c). Using CellRank and the CGSD, we identified early cells in the transition, from which we computed a pseudotime using Palantir (Supplementary Fig. 16). We combined pseudotime with the probability of transitioning towards the basal fate to define stages in the dedifferentiation trajectory in the data subset (Fig. 6e), splitting cells with at least 66% probability of reaching the basal state into three equal pseudotime bins. Stage 1 consists of goblet cells characterized by high expression of goblet marker *Bpifb1*. Stage 2 comprises an intermediate set of cells that express both *Bpifb1* and basal marker *Krt5*. Stage 3 consists of terminal basal cells, characterized by basal markers *Krt5* and *Trp63*, and no expression of *Bpifb1* (Fig. 6e).

Our new goblet cell dedifferentiation model predicts that, after injury, the frequency of stage 2 cells should increase as these represent intermediate cells in the dedifferentiation bridge towards basal cells. To validate this prediction, we assessed *Bpifb1*, *Krt5* and *Trp63* expression by immunofluorescence of mouse airway epithelial cells at days 10 and 21 after bleomycin treatment, as well as in untreated animals. Cells from stage 1 (goblet) and stage 3 (basal) were found in both control and treated mice. However, intermediate stage 2 cells were found only in 10-day post-treatment mice (Fig. 6f,g). Furthermore, we also found triple-positive cells, but these appeared only after injury (Supplementary Fig. 17). Goblet cell hyperplasia—an increase in the number of mucous secreting cells in the airways—is a prominent feature in several chronic inflammatory conditions^58^. The new dedifferentiation trajectory to basal stem cells that CellRank analysis predicted is unexpected, suggesting a route for generating multipotent stem cells in the resolution phase of the regenerative response to injury.

## Discussion

We have shown that CellRank combines gene expression similarity with RNA velocity to robustly estimate directed cellular trajectories in development, reprogramming and regeneration across experimental platforms (10x and Drop-seq). Applied to pancreatic development, CellRank outperformed existing methods by accurately recovering initial and terminal states, fate potentials and gene expression trends, efficiently computing terminal states (seconds) and fate potentials (few minutes) on 100,000 cells. Similarity-based trajectory approaches have been limited mainly to studying biological processes in which the starting cell and direction are clear. In contrast, CellRank generalizes beyond normal development, successfully recovering lineage-derived ground truth during in vitro fibroblast reprogramming and predicting a new goblet-to-basal cell dedifferentiation trajectory upon lung injury. We validated the existence of a new intermediate state between goblet and basal cells experimentally, although the direction of the proposed trajectory still needs to be confirmed with lineage tracing.

CellRank includes a number of innovations, including uncertainty propagation and high-dimensional vector field analysis. Other approaches attempting vector field analysis have either ignored the stochastic nature of fate decisions and velocity uncertainty^59^, or do not focus on trajectory reconstruction^60^. The original velocyto^14^ model proposed to find initial and terminal states by simulating a Markov process forwards or backwards in time; however, that implementation relied on a 2D t-SNE embedding that does not adhere to the phenotypic manifold or enable separation into individual initial and terminal states.

RNA velocity vectors are noisy estimates of the current state of gene regulation. CellRank takes care of uncertain velocity vectors by propagating their distribution in a manner that scales with local noise level and increases robustness. A current limitation is that we need to compute moments over velocity vectors in the local neighborhood to approximate their distribution. In future, we predict an end-to-end framework that propagates uncertainty from raw counts to end-state assignments and fate probabilities. We note that if the velocity vectors are systematically biased (for example, due to driver genes with insufficient unspliced reads to estimate their kinetics), then computed fate probabilities will reflect these biases, despite uncertainty propagation.

In contrast to previous Markov chain-based methods^7^, our approach is based on a directed nonsymmetric transition matrix. Eigenvectors of nonsymmetric transition matrices are generally complex and do not permit a physical interpretation, implying that it would not be possible to apply the eigendecomposition to learn about aggregate dynamics. This could be addressed using computationally expensive simulation-based approaches, but CellRank takes a more principled approach based on the real Schur decomposition—a generalization of the eigendecomposition to nondiagonalizable matrices.

In the pancreas data, CellRank identified alpha, beta and epsilon states automatically, but the delta macrostate required us to manually assign terminal status, likely because delta cells are rare in this dataset and their regulation is not detected correctly by velocities. To overcome the deficiencies of splicing data, it may be possible to extend the CellRank model to epigenetic information such as chromatin accessibility, leveraging the directional information coded by the typical delay between epigenetic and transcriptional changes^61,62^. Such information could be included by introducing limited memory to the Markov chain.

For delta cell development, we showed how gene expression can be correlated with fate probabilities to identify putative driver genes. Alternatively, drivers could be identified through statistical tests on the parameters of the generalized additive models (GAMS) used for fitting lineage-specific gene expression trends. Existing models could benefit from CellRank fate probabilities for assigning cells to lineages^63^. Further studies are needed to validate our new proposed markers for delta cell differentiation. We anticipate applying this framework to demonstrate its use on cycling cells and cancer contexts.

CellRank could also be extended by using temporal information, such as timepoints in the lung dataset^52^, to regularize the model, by only allowing transitions consistent with experimental time^64^. Further, lineage tracing information could regularize the model to obey clonal dynamics^65^. CellRank could also be easily applied to data from metabolic labeling^4–6^. As a general framework for interpreting high-dimensional vector fields, we anticipate that CellRank will be useful to describe complex trajectories in regeneration, reprogramming and cancer, where determining the direction of the process is often challenging.

## Methods

### The CellRank algorithm

The aim of the CellRank algorithm is to detect the initial, intermediate and terminal states of a system, and to define a global fate map that assigns, for each cell, the probability of reaching each terminal state. CellRank can compute gene expression trends along trajectories in the inferred fate map and visualize these in several ways.

Inputs to CellRank are a cell (*N*) by gene (*G*) count matrix *X*∈*R*^*N*×*G*^, and velocity matrix *V*∈*R*^*N*×*G*^ that defines a vector field representing RNA velocities^14,15^. Note that CellRank can use any vector field; for example, *V* could represent directed information given by metabolic labeling^4–6,73^. CellRank comprises three main steps:

1. Compute transition probabilities—the likelihood that a cell will transition from one state, defined by its gene expression profile, to another—by integrating two sources of evidence: transcriptomic similarity between the source and target cells and an extrapolation of a cell’s current expression profile into the near future using RNA velocity. We aggregate these probabilities in the transition matrix *P* and use it to model cell state transitions as a Markov chain.
2. Coarse-grain the Markov chain into a set of initial, terminal and intermediate macrostates, and assign each cell to each macrostate via membership matrix *χ*. The assignment is soft, meaning that each macrostate is assigned with a certain confidence. We compute transition probabilities among macrostates in the matrix *P*_c_, allowing us to classify macrostates as initial, terminal or intermediate.
3. Compute fate probabilities towards a subset of the macrostates (typically terminal states, but possibly also intermediate states, depending on the biological question). We compute how likely each cell is to transition into each of the selected macrostates and return these probabilities in a fate matrix *F*.

### CellRank extracts the essence of cellular state transitions

CellRank decomposes a biological system into a set of dynamical macrostates, associated with regions in the phenotypic manifold, which cells are unlikely to leave once they have entered. We compute how likely each cell is to belong to each macrostate and accumulate these soft assignments in a membership matrix $\chi \in {R^{N \times {n_s}}}$ where *n*_s_ is the number of macrostates. Further, we compute a coarse-grained t﻿ransition matrix ${P_c} \in {R^{{n_s} \times {n_s}}}$, which specifies transition probabilities among macrostates and reduces the biological system to its essence: dynamical macrostates of observed cell state transitions and their relationship to one another. We classify macrostates as initial, bearing very small incoming, but large outgoing, transition probability; terminal, with large incoming, very little outgoing and large self-transition probability; and intermediate, with both incoming and outgoing probabilities.

### CellRank computes probabilistic fate potentials

Next, CellRank efficiently computes the probabilities that each cell will transition to each of the *n*_t_ terminal states, and returns a fate matrix $F \in {R^{N \times {n_t}}}$. Matrix *F* extends the short-range fate relationships given by RNA velocity to the global scale: from initial to terminal states along the phenotypic manifold. We account for high noise levels in the velocity vectors via a stochastic Markov chain formulation, by restricting predicted transitions to align with the phenotypic manifold and by propagating velocity uncertainty into the Markov chain.

### CellRank uncovers gene expression trends towards specific terminal populations

The outputs of the CellRank algorithm are:

- Membership matrix $\chi \in {R^{N \times {n_s}}}$. Row *i* in *χ* softly assigns cell *i* to the set of *n*_s_ macrostates.
- Coarse-grained transition matrix ${P_c} \in {R^{{n_s} \times {n_s}}}$.
- Fate matrix $F \in {R^{N \times {n_t}}}$. Row *i* in *F* specifies how likely cell *i* is to transition towards any terminal state.

We use the fate matrix *F* to model gradual lineage commitment, which can be visualized jointly for all terminal states in CellRank by using circular projections. Fate biases can be aggregated to the cluster level and visualized as pie charts on a new directed version of PAGA graphs^8^. Further, we use *F* to fit gene expression trends towards the identified terminal states. Trends can be clustered to discover the main regulatory dynamics towards different terminal states. For the identification of putative regulators towards specific terminal states, we correlate gene expression values with fate probabilities.

#### Modeling approach

CellRank models cell state transitions but, unlike other velocity-based methods, it follows the success of pseudotime methods by restricting state changes to those consistent with the global structure of the phenotypic manifold (that is, a KNN graph based on gene expression similarity). Our approach biases the likely future state of an observed cell by combining transcriptional similarity with RNA velocity to direct edges in the graph, and assigns a probability to each cell state transition. When computing these probabilities, we take into account uncertainty in the velocity vectors. By aggregating individual, stochastic transitions in the global structure of the phenotypic manifold, we uncover the fate bias for individual cells. CellRank assumes that:

- State transitions are gradual; each state in the progression is, in general, transcriptomically similar to the previous state. Cells traverse a low-dimensional phenotypic manifold from initial to terminal states via a set of intermediate states.
- The set of sampled cellular profiles spans the entire state-change trajectory; that is, intermediate states have been covered and the trajectory has no ‘gaps’.
- While a cell’s history may be stored epigenetically, we model average cellular dynamics where state transitions occur without memory.
- RNA velocity approximates the first derivative of gene expression. This need not hold precisely for every gene in each cell as we treat state transitions as a stochastic process, enforce alignment with the manifold and propagate uncertainty, but it should hold for enough cells to enable estimation of the overall directional flow. In particular, this should hold for the main driver genes of the biological process. We urge users to assess this for their particular system by using scVelo’s dynamical model of splicing kinetics to check whether the top likelihood genes contain biological drivers, and whether their fits have converged. For example, insufficiently resolved splicing kinetics prevent the model from correctly resolving a small state of pancreatic terminal Delta cells (Extended Data Fig. 7).

Based on these assumptions, we model cellular state transitions using a Markov chain: a stochastic process *X* = (*X*_t_)_t∈*T*_ that models the evolution of the distribution of a random variable *X*_t_ over a state space *Ω* where the future distribution depends only on the current distribution and not on the past distribution, that is, ${\mathrm{Pr}}\, (X_{{\mathrm{t}}_{n + 1}} = x|X_{{\mathrm{t}}_1} = x_1,X_{{\mathrm{t}}_2} = x_2, \ldots ,X_{{\mathrm{t}}_n} = x_n) = {\mathrm{Pr}}\, (X_{{\mathrm{t}}_{n + 1}} = x|X_{{\mathrm{t}}_n} = x_n)$. The Markov chain traverses a discrete and finite state space Ω, where each state in the chain is given by an observed cellular transcriptional profile. To define the Markov chain, we need to compute a transition matrix *P*∈*R*^*N*×*N*^, which describes how likely one cell is to transition into another. We construct *P* using a KNN graph based on transcriptional similarity between cells and a given vector field. While CellRank generalizes to any given vector field, we demonstrate it using RNA velocities, based on unspliced-to-spliced read ratios, computed with scVelo^15^.

### Defining initial, intermediate and terminal states in biological terms

We define an initial (terminal) state as an ensemble of measured gene expression profiles which, when taken together, characterize the start (end) point of one particular sampled biological process. We define an intermediate state as an ensemble of gene expression profiles that characterize a point between initial and terminal states on the cell state transition trajectory.

### Translating initial, intermediate and terminal states into mathematical terms

The macrostates defined above can be derived mathematically although the membership matrix *χ* and the coarse-grained transition matrix *P*_c_. Our assignment of cells to macrostates maximizes ‘crispness’^74^—limited overlap between macrostates and large self-transition probabilities—as we show below. This procedure recovers the kinetics of the Markov chain over long timescales, that is, macrostates and their transitions reflect the limiting behavior of the Markov chain. We identify initial states as those macrostates with little incoming, but large outgoing, transition probability in *P*_c_. Intermediate states have both incoming and outgoing transition probability, and terminal states have large incoming, but little outgoing, and large self-transition probability. Macrostates are metastable—they define regions of phenotypic space that cells are unlikely to leave once they have entered. Terminal states are typically highly metastable, whereas intermediate states are typically only weakly metastable. Initial states can constitute weakly metastable states, if the probability of leaving is small, potentially because of heavily cycling populations.

### Reversing the Markov chain to recover initial states

If cells begin traversing their trajectory rapidly, initial states may not be stable enough to be identified as macrostates by coarse-graining the Markov chain. In these cases, we reverse the Markov chain, that is, we flip the arrows in the velocity vector field *V*. The initial state now constitutes a terminal (that is, metastable) state of the reversed dynamics and may be recovered by coarse-graining and interpreting the reversed Markov chain.

### Defining fate probabilities towards macrostates

Biologically, we define the probability that cell *i* will reach macrostate (fate) *j*∈{1,...,*n*_s_} as the probability that cell *i* executes a series of gene expression program changes to match the phenotype of cells in macrostate *j*. In the context of fate probabilities, we are typically interested in terminal or intermediate macrostates. Mathematically, we translate this to the probability of a random walk on the Markov chain initialized in cell *i* to reach any cell belonging to macrostate *j* before reaching any cell belonging to another macrostate. CellRank efficiently computes these probabilities in closed form using absorption probabilities.

#### Computing the transition matrix

We model each observed cell by one microstate in the Markov chain. To compute transition probabilities among cells, we make use of transcriptomic similarity to define the global topology of the phenotypic manifold and of RNA velocity to direct local movement on the manifold. To model the global topology of the phenotypic manifold, the first step of the CellRank algorithm is to compute a KNN graph.

### Computing a KNN graph to align local transitions with global topology

We compute a KNN graph to constrain the set of possible transitions to those consistent with the global topology of the phenotypic manifold; each cell can only transition into a nearest neighbor. While CellRank can generalize to any similarity kernel, we compute the KNN graph here as follows:

- Project the data onto the first *L* PCs to obtain a matrix *X*_PC_∈*R*^*N*×*L*^, where rows correspond to cells and columns correspond to PC features.
- For each cell *i*, compute Euclidean distances to its K nearest neighbors in *X*_PC_. Accumulate distances in a matrix *D*∈*R*^*N*×*N*^.
- The KNN relationship will lead to a directed graph because it is not a symmetric relationship. Symmetrize the KNN relations encoded by *D*, such that cells *i* and *j* are nearest neighbors if either *i* is a nearest neighbors of *j*, or *j* is a nearest neighbors of *i*. This will yield an undirected symmetric version *D*_sym_ of *D*, where each cell has at least *K* nearest neighbors.
- Compute a symmetric adjacency matrix *A* based on *D*_sym_ containing similarity estimates between neighboring cells according to the manifold structure. To approximate cell similarities, we use the method implemented in the UMAP algorithm, which adapts the singular set and geometric realization functors from algebraic topology to work in the context of metric spaces and fuzzy simplicial sets^28,75^.

We choose *K* = 30 nearest neighbors by default, but CellRank is robust to the choice of *K* (Supplementary Fig. 6b). The default similarity metric is that of SCANPY^76^, although similarity may be computed using a Gaussian kernel with density-scaled kernel width as introduced by Coifman et al.^77^ and adapted to single-cell context by Haghverdi et al.^7^. The number of PCs is *L* = 30 by default, but can be adjusted based on knee-point heuristics or the percentage of variance explained. CellRank is robust to the exact choice of *L* (Supplementary Fig. 6e).

### Directing the KNN graph based on RNA velocity

Next, we direct the edges of the KNN graph using RNA velocity information, giving higher probability to those neighbors whose direction best aligns with the direction of the velocity vector. Specifically, for cell i with gene expression profile *x*_i_∈*R*^G^ and velocity vector *v*_i_∈*R*^G^, consider its neighbors *j*∈{1,2,...,*K*_i_} with gene expression profiles {*x*_1_,*x*_2_,...,*x*_*K*i_}. Note that the graph construction outlined above leads to a symmetric KNN graph, where *K*_i_ is not constant across all cells, but *K*_i_≥*K*∀*i*∈{1,...,*N*}. For each neighboring cell *k*, compute the corresponding state-change vector with cell i, *s*_ik_ = *x*_k_−*x*_i_∈*R*^*G*^. Next, we compute Pearson correlations ${c_i} \in {R^{{K_i}}}$ of *v*_i_ with all state-change vectors {*s*_ik_}. Intuitively, *c*_i_ contains the cosines of the angles that the mean-centered *v*_*i*_ forms with the mean-centered state-change vectors {*s*_ik_}. A value of one means perfect correlation between the gene expression changes predicted by the local velocity vector and the actual change observed when going from the reference cell to any of its nearest neighbors. Pearson correlations have been computed in similar ways by scVelo^15^ and velocyto^14^ to project the velocity vectors into a given embedding. In the following, we show how these ideas can be formalized and extended to account for uncertainty in the velocity vector. CellRank’s final transition matrix differs fundamentally from velocyto’s, with important implications for identifying rare populations and local dynamics.

### Transforming correlations into transition probabilities

To use the vector *c*_i_ as a set of transition probabilities to neighboring cells, we need to make sure it is positive and sums to one. For cell i, define a set of transition probabilities ${p_i} \in {R^{{K_i}}}$ via

$$p_{\mathrm{ik}} = \frac{{e^{\sigma c_{\mathrm{ik}}}}}{{\mathop {\sum }\nolimits_\mathrm{l} e^{\sigma c_{\mathrm{il}}}}},$$

where *σ* > 0 is a scalar constant that controls how centered the categorical distribution will be around the most likely value, that is around the state-change transition with maximum correlation (below). We repeat this for all (*i*, *k*) which are nearest neighbors to compute the transition matrix *P*_*v*_∈*R*^*N*×*N*^. This scales linearly in the number of cells (*N*), nearest neighbors (*K*) and genes (*G*), as the KNN graph is sparse.

### Automatically determine *σ*

Reasoning that *σ* should depend on typical Pearson correlation between velocity vectors and state-change vectors observed in the dataset, we use the heuristic:

$$\sigma = \frac{1}{{{{{\mathrm{median}}}}(\{ |c_{\mathrm{ik}}|\forall (\mathrm{i,k})\} )}}.$$

Thus, if the median absolute Pearson correlation observed in the data is large (small), we use a small (large) value for *σ*. The intuition behind this is to slightly upscale all correlations for sparsely sampled datasets, where velocity vectors point only roughly in the direction of neighboring cells. Values for *σ* computed this way range from 1.5 for the lung example^52^ to 3.8 for the pancreas example^24^.

### Coping with uncertainty in the velocity vectors

scRNA-seq data is a noisy measurement of the gene expression state of individual cells. RNA velocity is derived from these measurements and is itself therefore very noisy. In particular, the unspliced reads required to estimate velocities are very sparse, and their abundance varies by the amount of relevant intronic sequence per gene. Besides this inherent noise, preprocessing decisions in the alignment of spliced and unspliced reads impact the final velocity estimate^78^. Further uncertainty in the velocity estimate arises because modeling assumptions may not always be satisfied:

- The velocyto^14^ model assumes that the data captures the steady state of each gene. The scVelo^15^ model circumvents this assumption by dynamic modeling, extending RNA velocity to transient cell populations; however, only a few transitional cells are available to estimate these dynamics.
- Both models assume that the key driver genes for a given transition are intron-rich and may therefore be used to estimate splicing ratios. This assumption has been shown to hold in many neurological settings, but remains unclear in systems such as hematopoiesis. In our pancreas analysis, *Cd24a* is an example of a gene that is expressed in most cells (62%), but only has unspliced counts in three cells (Extended Data Fig. 7b). This gene is important for delta cell development, yet it has too few unspliced counts to robustly compute RNA velocity.
- Both models assume that a single set of per-gene kinetic parameters *α* (transcription rate), *β* (splicing rate) and *γ* (degradation rate) may be used across all cells, but this assumption is often violated because of alternative splicing or cell-type-specific regulation^79–82^.
- Both models assume no batch effects in the data. To the best of our knowledge, there are currently no tools to correct for batch effects in velocity estimates.
- Both models assume that cell state transitions captured in the data are compatible with the time scale of splicing kinetics. However, this is often not known a priori and may explain the limited success of RNA velocity in studying hematopoiesis so far.

To cope with the substantial uncertainty present in RNA velocity, we adapt four strategies:

- We restrict the set of possible transitions to those consistent with the global topology of the phenotypic manifold as described by the KNN graph.
- We use a stochastic formulation based on Markov chains to describe cell state transitions. For cell i with velocity vector *v*_i_, we allow transitions to each nearest neighbor j with transition probability *p*_ij_. This means that we even allow backward transitions, against the flow prescribed by the velocity vector field, with small probability. This reflects our uncertainty in *v*_i_.
- We combine RNA velocity information with transcriptomic similarity.
- We propagate uncertainty in *v*_i_ into the downstream computations.

### Emphasizing transcriptomic similarity

Thus far, we have combined RNA velocity with transcriptomic similarity by computing a similarity-based KNN graph to restrict the set of possible transitions. To further take advantage of the information captured by the KNN graph and to increase robustness of the algorithm to noisy velocity vectors, we combine the velocity-based transition matrix *P*_v_ with a similarity-based transition matrix *P*_s_ via

$$P = (1 - \lambda )P_{\mathrm{v}} + \lambda P_{\mathrm{s}}\;{\mathrm{for}}\; \lambda \in [0,1].$$

The matrix *P*_s_ is computed by row-normalizing the adjacency matrix *A*, which we introduced above in the context of the KNN graph. The parameter *λ* defines how much weight is given to the connectivity-based (that is, transcriptomic similarity-based) transition probabilities. In practical applications, we have found that using values around *λ* = 0.2 increase robustness with respect to noisy velocity estimates. The matrix *P* is the final transition matrix estimated by the CellRank algorithm.

#### Coarse-graining the Markov chain

Each cell in the transition matrix *P* constitutes a microstate of the Markov chain, but it is difficult to interpret the cellular trajectory directly from *P* because it is a fine-grained, noisy representation of cell state transitions. We seek to reduce *P* to its essence: macrostates, representing key biological states, and the probabilities of transitioning between them. We accomplish this using pyGPCCA, which uses the GPCCA^19,20,83^—a method developed to study conformational dynamics in proteins. We adapt it to the single-cell setting and use it to project *P* onto a much smaller coarse-grained transition matrix *P*_c_ that describes transitions among macrostates. A macrostate is associated with a subset *M* of the state space *M*⊂*Ω*. The macrostates are defined through a so-called membership matrix *χ*. Rows of *χ* contain the soft assignment of each cell to the set of macrostates.

### Generalized Perron Cluster Cluster Analysis

For the projected or embedded dynamics to be Markovian, we require the projection of *P* onto *P*_c_ to be based on an invariant subspace of *P*, that is, a subspace *W* for which

$$P^ \top x \in W \; \forall x \in W.$$

In the case of reversible *P*, invariant subspaces are spanned by the eigenvectors of *P*^74^. In our case, however, *P* is nonreversible and the eigenvectors will, in general, be complex. Since the GPCCA method cannot cope with complex vectors, we rely on real invariant subspaces of the matrix *P* for the projection. Such subspaces are spanned by the real Schur vectors of *P*^19,20,84^ that are provided by a real Schur decomposition

$$P = QRQ^ \top .$$

The columns of the matrix *Q*∈*R*^*N*×*N*^ are the Schur vectors and the Schur form *R*∈*R*^*N*×*N*^ is quasi-upper triangular^85^. *R* has 1 × 1 or 2 × 2 blocks on the diagonal, where the former are given by the real eigenvalues and the latter are associated with pairs of complex conjugate eigenvalues.

### Invariant subspaces of the transition matrix

Columns of *Q* corresponding to real eigenvalues span real invariant subspaces. Columns of *Q* corresponding to pairs of complex conjugate eigenvalues span real invariant subspaces when kept together, but not if they are separated. Particularly, for columns *q*_j_ and *q*_k_ of *Q* belonging to a pair of complex conjugate eigenvalues, the space *W*_0_ = span(*q*_j_, *q*_k_) is invariant under *P*, but the individual *q*_j_ and *q*_k_ are not^86^. Depending on the constructed subspace, different dynamical properties of *P* will be projected onto *P*_c_. Choosing Schur vectors belonging to real eigenvalues close to 1, metastabilities are recovered, while for Schur vectors with complex eigenvalues close to the unit circle, cyclic dynamics are recovered^19,20^. Both options are available in CellRank, defaulting to the recovery of metastabilities.

### Projecting the transition matrix

Let $\tilde Q \in R^{N \times n_\mathrm{s}}$ be the matrix formed by selecting *n*_s_ columns from *Q* according to some criterion (metastability or cyclicity). Let $\chi \in R^{N \times n_\mathrm{s}}$ be a matrix obtained via linear combinations of the columns in $\tilde Q$, that is

1 $$\chi = \tilde QA,$$

for an invertible rotation matrix $A \in R^{n_\mathrm{s} \times n_\mathrm{s}}$. Rows of *χ* define membership to macrostates; we describe *χ* and *A* in more detail below. We obtain the projected transition matrix via an invariant subspace projection^19,20^,

$$P_\mathrm{c} = \left(\chi ^ \top D\chi \right)^{ - 1}\left(\chi ^ \top DP\chi\right),$$

where *D* is the diagonal matrix of a weighted scalar product. The Schur vectors in $\tilde Q$ must be orthogonal with respect to this weighted scalar product, that is $\tilde Q^{\it{ \top }}D\tilde Q = I$ with the *n*_s_-dimensional unit matrix *I*, to yield the required invariant subspace projection. The diagonal elements of *D* are in principle arbitrary, but a convenient choice would be the uniform distribution or some distribution of the cellular states of interest, for example, the stationary distribution, if it exists. Choosing the uniform distribution, as is the default in CellRank, would result in an indiscriminate handling (without imposing any presumptions about their distribution) of the cellular states. Note that the matrix inversion above is performed on a very small matrix of size *n*_s_×*n*_s_.

### Properties of the invariant subspace projection

Coarse-grained transition probabilities among macrostates are defined via an invariant subspace projection of *P* onto the set of macrostates. More precisely, *P* is projected onto a low-dimensional invariant subspace defined by the membership vectors *χ*, which are linear transformations of the Schur vectors. By applying an invariant subspace projection, the projection error vanishes and the coarse-graining operation commutes with the propagation operation^20,87^. In other words, given an initial density over cell states, the following yield the same result: (1) propagating the cell density using the original matrix *P* and then projecting the propagated density onto the set of macrostates, or (2) projecting the initial cell density onto the set of macrostates and propagating it using *P*_c_. It follows that the projected, coarse-grained Markov chain preserves the slow timescales of the process, that is the transitions between metastable subsets of the phenotypic manifold^20^.

### Computing membership vectors

In principle, we could use any invertible rotation matrix *A* above. However, we would like to interpret the columns of *χ* as membership vectors that define assignment weights for all cells. For this reason, we seek a matrix that minimizes the overlap between membership vectors in *χ*, that is, a rotation matrix *A* that minimizes off-diagonal entries in $\chi ^{\it{ \top }}D\chi$. This is equivalent to maximizing

$$\mathrm{trace}(S) = \mathrm{trace}\left(\tilde D^{ - 1}\chi ^ \top D\chi \right).$$

The matrix $\tilde D^{ - 1}$ chosen to row-normalized can be expressed as

$$\tilde D^{ - 1} = \mathrm{diag}\left( {\frac{1}{{\mathop {\sum }\nolimits_\mathrm{j} (\chi ^TD\chi )_{1\mathrm{j}}}},...,\frac{1}{{\mathop {\sum }\nolimits_\mathrm{j} (\chi ^TD\chi )_{n_s\mathrm{j}}}}} \right)$$

Choosing Schur vectors with real eigenvalues close to one, thus recovering metastability, maximizing trace(*S*) can be interpreted as maximizing the metastability of the macrostates in the system. In practice, we minimize

2 $$f_{n_{\mathrm{s}}}\left( A \right) = n_{\mathrm{s}} - {{{\mathrm{trace}}}}\left( S \right),$$

as our objective function where *S* is a function of *A* (above). This objective function is bounded below by zero and convex on the feasible set defined through linear constraints^74^. We must minimize *f*_ns_ with respect to the constraints

$$\chi _{\mathrm{ij}} \ge 0\; \forall i \in \{ 1,...,N\} \; \forall {\mathrm{j}} \in \{ 1,...,n_{\mathrm{s}}\} \; ({{{\mathrm{positivity}}}}),$$

$$\mathop {\sum }\limits_{\mathrm{j}} \chi _{\mathrm{ij}} = 1\; \forall i \in \{ 1,...,N\} \; ({{{\mathrm{partition}}}}\,{{{\mathrm{of}}}}\,{{{\mathrm{unity}}}}).$$

We can re-express these conditions, using equation (1) and a result from Weber^88^ such that they can be written in terms of the invertible rotation matrix *A* and the matrix of selected Schur vectors ${\tilde{Q}}$,

$$A(1,{\mathrm{j}}) = - \mathop {{{{{\mathrm{min}}}}}}\limits_{{\mathrm{l}} = 1,...,N} \mathop {\sum }\limits_{i = 2}^{n_{\mathrm{s}}} \tilde Q({\mathrm{l,i}}) \, A({\mathrm{i,j}}) \; \forall {\mathrm{j}} \in \{ 1,...,n_{\mathrm{c}}\} \;({{{\mathrm{positivity}}}}),$$

$$A({\mathrm{i}},1) = \delta _{{\mathrm{i}},1} - \mathop {\sum }\limits_{{\mathrm{j}} = 2}^{n_{\mathrm{s}}} A({\mathrm{i,j}})\; \forall {\mathrm{i}} \in \{ 1,...,n_{\mathrm{c}}\} \; ({{{\mathrm{partition}}}}\,{{{\mathrm{of}}}}\,{{{\mathrm{unity}}}}).$$

Optimizing equation (2) subject to these constraints is no trivial task. Among the several possibilities to solve the optimization problem, a convenient choice is to perform unconstrained optimization on ${A_{2:{n_s},2:{n_s}}}$ using a trick: to impose the constraints after each iteration step, thus transforming the unfeasible solution into a feasible solution^74^. However, this approach is nondifferentiable. Therefore, in CellRank, we use the derivative-free Nelder-Mead method as implemented in the Scipy routine scipy.optimize.fmin^89^ for the optimization.

### Positivity of the projected transition matrix

Note that *P*_c_ may have negative elements if macrostates share a large overlap. In practice, this is caused by a suboptimal number of macrostates *n*_s_ and can be resolved by changing that number. We may interpret *P*_c_ as the transition matrix of a Markov chain between the set of macrostates if it is non-negative within numerical precision^20^.

### Tuning the number of macrostates

The number of macrostates *n*_s_ can be chosen in a number of ways, all available through CellRank:

- Using the eigengap heuristic for the real part of the eigenvalues close to one.
- Define the crispness *ξ* of the solution as the value of ${{{\mathrm{trace}}}}(\tilde D^{ - 1}\chi ^{\it{ \top }}D\chi )/n_\mathrm{s}$, see Röblitz and Weber^74^. The larger this value, the smaller the overlap between the macrostates, and, in turn, the sharper or ‘crisper’ the recovered macrostates. Crispness can be computed for different numbers of macrostates *n*_s_ and the number *n*_s_ with the largest value of *ξ* should be selected.
- To avoid having to solve the full problem for too many values of *n*_s_, do a preselection using the minChi criterion^74^: based on an initial guess for *A*, compute a membership matrix *χ* and calculate minChi = min_i,j_(*χ*_ij_). In general, this value will be negative because the starting guess is infeasible. The closer to zero the value of minChi, the more we can expect *n*_s_ to yield a crisp decomposition of the dynamics.
- Combining the minChi criterion and the crispness to avoid solving the full problem for many *n*_s_, but still select the *n*_s_ with the crispest decomposition. This is done by first selecting an interval of potentially good numbers of macrostates *n*_s_ via the minChi criterion and afterwards using the crispness to select the best *n*_s_ from the preselected macrostate numbers.

### Scalable Python implementation of GPCCA

Following the original MATLAB implementation^90^, we wrote up GPCCA as a general algorithm in Python and created a package for it: pyGPCCA^91^. pyGPCCA comes with a comprehensive documentation and testing suite to make sure it is easily maintainable and extendable. While pyGPCCA serves as the backbone for CellRank, we anticipate it to be used outside the single-cell community as well, for example in the study of protein conformational dynamics. A naive implementation of the Schur decomposition would scale cubical in cell number. We alleviate this problem by using SLEPc to compute a sorted partial real Schur decomposition using an iterative, Krylov-subspace-based algorithm that optimally exploits the sparsity structure of the transition matrix^92,93^. Overall, this reduces the computational complexity of our algorithm to be roughly linear in cell number (Fig. 5d and Supplementary Table 1). This allows CellRank to scale well to very large cell numbers.

### Determine terminal states

To automatically identify terminal states, we look for the most stable macrostates in the coarse-grained transition matrix *P*_c_. Define the SI of a macrostate *m*∈{1,...,*n*_s_} through its corresponding diagonal value in *P*_c_, that is, through its self-transition probability ${P_{{c_{mm}}}}$. The intuition behind this is that cells in terminal populations should have very little probability to transition to cells in other populations and should distribute most (if not all) of their probability mass to cells from the same terminal population. To identify the number of terminal states, we set a threshold on SI; that is, we classify all states as terminal for which $\mathrm{SI} \ge {\it{\epsilon }}_{\mathrm{SI}}$ with ${\it{\epsilon }}_{\mathrm{SI}} = 0.96$ by default.

### Determine initial states

To automatically identify initial states, we introduce the CGSD $\pi _\mathrm{p} \in R^{n_\mathrm{s}}$ given by

$$\pi _\mathrm{p} = \chi ^ \top \pi$$

where $\pi \in R_ + ^N$ is the stationary distribution of the original transition matrix *P*. The stationary distribution satisfies

$$\pi ^ \top P = \pi ^ \top {{{\mathrm{and}}}}\,\mathop {\sum }\limits_\mathrm{i} \pi _\mathrm{i} = 1.$$

In other words, the stationary distribution *π* is an invariant measure of *P* and can be computed by normalizing the top left eigenvector of *P* (corresponding to eigenvalue 1). Under certain conditions (ergodicity^94^) imposed on the Markov chain, the stationary distribution is the distribution that the process converges to if it evolves for long enough, that is, it describes the long-term evolution of the Markov chain. In the same vein, the CGSD *π*_c_ describes the long-term evolution of the Markov chain given by *P*_c_. The CGSD *π*_c_ assigns large (small) values to macrostates that the process spends a large (little) amount of time in, if it is run infinitely long. As such, we may identify initial states by looking for macrostates that are assigned the smallest values in *π*_c_. The intuition behind this is that initial states should be states that the process is unlikely to visit again once it has left them. The number of initial states is a parameter with a default of one, which can be set to detect several initial states.

### Determine intermediate state

Remaining macrostates, which have been classified as neither terminal nor initial, are classified as intermediate. Intermediate states in developmental processes usually have a consistent signal of moving onto more mature states, even if there is some pausing, and are therefore placed correctly on the phenotypic manifold by the KNN graph. If this movement signal is in *P*, it will also be present in *P*_c_. As long as RNA velocity vectors roughly capture the direction of differentiation for intermediate states, CellRank will correctly tell apart intermediate from terminal states by restricting the velocity vectors to be consistent with the local manifold structure.

### Handling reducible Markov chains

A Markov chain is irreducible if it is possible to get from any state to any other state in a finite number of transitions (it is reducible if not). Our transition matrix construction ensures that, as long as the underlying KNN graph is connected, the resulting Markov chain will be irreducible. That is because we allow for transitions against the direction of the local RNA velocity vector, with small probability. If the KNN graph is not connected, then the resulting Markov chain will be reducible; for example, there is an outlier cell type that does not participate in the main dynamics of the data. Reducible Markov chains pose no problem to GPCCA. In the example, the outlier cell type will be assigned its own macrostate with no transition probability to other macrostates in *P*_c_. Likewise, there will be no incoming transition probability to the outlier macrostate, thus making it easy for the user to identify this in *P*_c_ as a macrostate that does not participate in the overall dynamics. To compute the initial states among the remaining macrostates, it is best to exclude the outlier macrostate. Upon exclusion, the remaining coarse-grained Markov chain will be irreducible again, hence a unique stationary distribution exists that can be used to identify the initial states, as described above.

#### Computing fate probabilities

Given the soft assignment of cells to macrostates by *χ* and the identification of terminal states through *P*_c_, we compute how likely each cell is to transition towards these terminal states. Let *n*_t_ be the number of terminal states. For the sake of clarity, we only describe fate probabilities towards terminal states; however, the computations below apply just as well to intermediate states, if that is the biological question. For each terminal macrostate t for t∈{1,...,*n*_t_}, we choose *f* cells that are strongly assigned to *t* according to *χ*. That is, for terminal macrostate t, we extract the corresponding column from *χ* and we calculate the terminal index set *R*_t_ of cells that have the largest values in this column of *χ*. If cell *i* is part of the terminal index set *R*_t_, we assume cell i is among the *f* most eligible cells to characterize the terminal macrostate t in terms of gene expression. We store the indices of the remaining cells in the transient index set *T*. The index sets {*R*_t_|t∈{1,...,*n*_t_}} and *T* form a disjoint partition of the state space, which means they do not overlap and they cover the entire state space. For each cell i in *T*, we would like to compute a vector of probabilities $f_\mathrm{i} \in R^{n_\mathrm{t}}$ which specifies how likely this cell is to transition into any of the terminal states characterized through {*R*_t_}. To interpret *f*_i_ as a categorical distribution over cell fate, we require *f*_i,t_≥0∀_i_∈*T*∀*t*∈{1,...,*n*_t_} and $\mathop {\sum }\limits_\mathrm{t} f_{\mathrm{it}} = 1\forall \mathrm{i} \in T$. We accumulate the *f*_i_ column-wise in the fate matrix $F \in R^{N \times n_\mathrm{t}}$.

### Absorption probabilities disclose cell fates

We could approximate the *f*_i_ based on sampling: initialize a random walk on the Markov chain in cell *i*; walk until any cell from a terminal set *R*_t_ is reached; record t and repeat this many times; and finally, count how often random walks initialized in cell i terminated in any of the terminal index sets *R*_t_. In the limit of repeating this infinitely many times, the normalized frequencies over reaching either terminal set will be equal to the desired fate probabilities for cell i, under reasonable assumptions on the Markov chain (irreducibility). Luckily, this does not require a sampling-based approach, as we can leverage a closed-form solution: absorption probabilities.

### Computing absorption probabilities

Key to the concept of absorption probabilities are recurrent and transient classes, which we will define here for the present case of a finite and discrete state space. Let *i*∈Ω and *j*∈Ω be two states of the Markov chain. In our case, i and j are cells. We say that i is accessible from j, if and only if, there exists a path from j to i according to the transition matrix *P*. A path is a sequence of transitions which has nonzero transition probability. Further, i and j communicate if, and only if, i is accessible from j and j is also accessible from i. Communication defines an equivalence relation on the state space *Ω*, that is, it is a reflexive, symmetric and transitive relation between two states^94^. It follows that the state space *Ω* can be partitioned into its communication classes {*C*_1_,...,*C*_k_}. The communication classes are mutually disjoint nonempty and their union is *Ω*. In other words: any two states from the same class communicate, states from different classes never communicate. We call a communication class *C*_j_ *closed* if the submatrix of *P* restricted to *C*_j_ has all rows sum to one. Intuitively, if *C*_j_ is closed, then a random walk which enters *C*_j_ never leaves it again. Closed communication classes are also called recurrent *classes*. If a communication class is not recurrent, we call it transient. In Theorem 1, we reproduce the statement of Thm. 28 in Tolver^94^ to compute absorption probabilities towards states that belong to recurrent classes on the Markov chain.

#### Theorem 1—absorption probabilities

Consider a MC with transition matrix *P∈R*^*N×N*^. We may rewrite *P* as

$$\left[ {\begin{array}{*{20}{c}} {\tilde P} & 0 \\ S & Q \end{array}} \right],$$

where $\tilde P$ and *Q* are restrictions of *P* to recurrent and transient states, respectively, and *S* is the restriction of *P* to transitions from transient to recurrent states. The upper right 0 is due to the fact that there are no transitions back from recurrent to transient states. Define the matrix *M* = (*I* − *Q*)^−1^.Then, the ijth entry of M describes the expected number of visits of the process to state j before absorption, conditional on the process being initialized in state i. M is often referred to as the fundamental matrix of the MC. Further, the matrix *A* = (*I* − *Q*)^−1^*S* contains, in the ijth entry, the probability of j being the first recurrent state reached by the MC, given that it was started in i.

For a proof, see Thm. 28 in Tolver^94^. To compute fate probabilities towards the terminal index sets *R*_t_ defined above, we approximate these as recurrent classes; that is, we remove any outgoing edges from these sets. We then apply Theorem 1, which, for each cell i∈*T* yields absorption probabilities towards each of the *f* cells in each of the *n*_*t*_ recurrent index sets. We aggregate these to yield absorption probabilities towards *R*_t_ by summing absorption probabilities towards individual cells in these sets.

### CellRank provides an efficient implementation to compute absorption probabilities

A naive implementation of absorption probabilities scales cubically in the number of transient cells due to the matrix inversion *A* = (*I* − *Q*)^−1^*S*. The number of transient cells is smaller than the total cell number only by a small constant, so the naive approach can be considered cubic in total cell number. This will inevitably fail for large cell numbers. We alleviate this by rewriting the above as a linear problem,

3 $$(I - Q)A = S.$$

Note that *Q* is very sparse as it describes transitions between nearest neighbors. Per row, *Q* has approximately *K* entries. To exploit the sparsity, iterative solvers are very appealing as their per iteration cost applied to this problem is linear in cell number and in the number of nearest neighbors. To apply an iterative solver, we must, however, rewrite equation (3) such that the right-hand side is vector valued,

$$(I - Q)a_1 = s_1,...,(I - Q)a_{fn_\mathrm{t}} = s_{fn_\mathrm{t}},$$

where *fn*_t_ is the total number of cells which belong to approximately recurrent classes. To solve these individual problems, we use the iterative GMRES^95^ algorithm which efficiently exploits sparsity. For optimal performance, we use the PETSc implementation, which makes use of efficient message passing and other practical performance enhancements. Finally, we parallelize solving the *fn*_t_ linear problems. Taken together, these tricks allow us to compute absorption probabilities quickly even for large cell numbers (Fig. 5d and Supplementary Table 1).

### Visualizing fate probabilities through circular embeddings

In this presentation, we follow work by Velten et al.^50^, which in turn is based on circular a posteriori projections^72^. Let $F \in {R^{N \times {n_t}}}$ by the matrix of fate probabilities for *N* cells and *n*_t_ terminal states such that *F*_i,:_ contains the fate probabilities for cell i. We seek a 2D arrangement of cells that reflects their fate probabilities. Therefore, we evenly space terminal states around the unit circle and assign each state an angle *α*_t_. We then transform each cell’s vector of fate probabilities *F*_i,:_ into a 2D representation (*x*_i_, *y*_i_) using

$$x_\mathrm{i} = \mathop {\sum }\limits_\mathrm{t} f_{\mathrm{it}}\cos \alpha _\mathrm{t},$$

$$y_\mathrm{i} = \mathop {\sum }\limits_\mathrm{t} f_{\mathrm{it}}\sin \alpha _\mathrm{t}.$$

As the representation depends on the order in which terminal states are arranged around the unit circle, we compute pairwise similarities among fate probabilities *F*_:,t_ corresponding to each terminal state t, and we choose the arrangement that maximizes pairwise similarities. By default, we use cosine correlation to quantify similarity.

### Quantifying multilineage potential through fate probabilities

CellRank provides two ways of quantifying multilineage potential on the basis of computed fate probabilities:

- through *S*_i_, the entropy over fate probabilities *F*_i,:_ (called ‘diffusion potential’ in Palantir^21^)
- through $KL(F_{\mathrm{i},:}||\overline F _:)$, the Kullback–Leibler (KL) divergence between fate probabilities *F*_*i*,:_ and the average fate probability per lineage across cells $\overline F _:$ (called ‘priming degree’ in STEMNET^50^)

Intuitively, *S*_*i*_ quantifies how far from uniform the distribution *F*_*i*,:_ is and $KL(F_{i,:}||\bar F_:)$ quantifies how far from the average fate distribution *F*_*i*,:_ is. The higher *S*_*i*_ and the lower $KL(F_{i,:}||\bar F_:)$, the more uncommitted a cell is. In situations where the initial cells are already expected to have a dominant direction of fate bias, we suggest using the KL divergence, as it will increase monotonically as cells move to terminal states while the entropy will reach its maximum at the point between initial and terminal states that come closest to uniform.

#### Propagating velocity uncertainty

So far, we have assumed that individual velocity vectors are deterministic, that is, they have no measurement error. However, RNA velocity is estimated on the basis of spliced and unspliced gene counts, which are noisy quantities. Hence, the velocity vectors *v*_i_ themselves should be treated as random variables that follow a certain distribution. Our aim is to propagate the distribution over *v*_i_ into our final quantities of interest—state assignments and fate probabilities—but no closed-form equation relates these final quantities to *v*_i_. A possible solution is to use an MC scheme where we draw velocity vectors, compute final quantities based on the draw and repeat this many times. In the limit of infinite draws, this will give us the distribution over final quantities, given the distribution in *v*_i_. However, we need to repeat our computation many times, which will become prohibitively expensive for large datasets. To get around this problem, we construct an analytical approximation to the MC-based scheme. This approximation will have to be evaluated only once and we can omit the sampling. We show, in a practical example, that the analytical approximation gives very similar results to the sampling-based scheme and improves over a deterministic approach by a large margin.

### Modeling the distribution over velocity vectors

Before we can propagate uncertainty, we need to model the uncertainty in the velocity vectors estimated by scVelo^15^ or velocyto^14^. Ideally, these packages would model uncertainty in the raw spliced and unspliced counts and propagate this into a distribution over velocity vectors. However, as that is currently not the case, we will make an assumption about their distribution and use the KNN graph to fine-tune expectation and variance by considering neighboring velocity vectors. To ease notation and illustrate core ideas, we will drop the subscript i in this section and focus on one fixed cell and its velocity vector *v*. Let’s assume that *v* follows a multivariate normal (MVN) distribution,

$$v \sim N(\mu ,{\Sigma}_v),$$

with mean vector *μ*∈*R*^G^ and covariance matrix Σ_*v*_∈*R*^G×G^. The MVN is a reasonable choice here as velocities can be both positive and negative and, for most genes, as we expect to see both up- and downregulation, velocity values will be approximately symmetrical around their expected value. Let us further assume the covariance matrix to be diagonal, that is, gene-wise velocities are independent—a reasonable assumption, as gene-wise velocities in velocyto^14^ and scvelo^15^ are computed independently. To compute values for *μ* and Σ_*v*_, consider velocity vector *v* and its *K* nearest neighbors. To estimate *μ* and the diagonal elements of Σ_*v*_, we compute first- and second-order moments over the velocity vectors of these neighboring cells.

### Propagating uncertainty into state assignments and fate probabilities

We seek to approximate the expected value of the final quantities of interest (state assignments and fate probabilities), given the distribution in the velocity vectors. Let *q* be a final quantity of interest. There are two main steps involved in computing *q*,

$$v \to T \to q,$$

where *v* stands for our inputs, that is, the velocity vectors, and *T* is the transition matrix defining the Markov chain. To get from *v* to *T*, we evaluate an analytical function that computes correlations and applies a softmax function. We can approximate this first part of the mapping with a Taylor series, which allows us to propagate analytically the distribution in *v* into *T*. For the second part of the mapping, we use the expected transition matrix to compute *q*. This yields an approximation to the expectation of the final quantity that we can then compare with the approximation we obtain from a MC scheme, which we treat as our ground truth.

### Approximating the expected transition matrix

In the first step, we compute the expected value of the transition matrix, given the distribution of the velocity vectors. Given a particular draw *v* from the distribution and a set of state-change vectors {*s*_k_}, we compute a vector of probabilities *p*, which lives on a *K*-simplex in *R*^K^. Let us denote the mapping from *v* to *p* by *h*,

$$h:R^\mathrm{G} \to R^\mathrm{K},$$

$$v \mapsto h(v) = p.$$

We can then formulate our problem as finding the expectation of *h* when applied to *v*, that is

$$E[h(v)]_{v \sim N(\mu ,{\Sigma}_v)}.$$

To approximate this, expand the ith component of *h* in a Taylor series around *μ*,

$$h_\mathrm{i}(v) = h_\mathrm{i}(\mu ) + \nabla _v^ \top h_\mathrm{i}(v)|_\mu (v - \mu ) + \frac{1}{2}(v - \mu )^ \top \nabla _v^2h_\mathrm{i}(v)|_\mu (v - \mu ) + O(v - \mu )^3.$$

Define the Hessian matrix of *h*_i_ at *v* = *μ* as

$$H^{(\mathrm{i})} = \nabla _v^2h_\mathrm{i}(v)|_\mu .$$

Taking the expectation of *h*_i_ and using the Taylor expansion,

$$E[h_\mathrm{i}(v)] \approx h_\mathrm{i}(\mu ) + \frac{1}{2}E[(v - \mu )^ \top H^{(\mathrm{i})}(v - \mu )].$$

Note that the first-order term cancels as *E*[*v*−*μ*] = 0. The second-order term can be further simplified by explicitly writing out the matrix multiplication,

$$E[(v - \mu )^ \top H^{(\mathrm{i})}(v - \mu )] = \mathop {\sum }\limits_{\mathrm{j,k} = 1}^G H_{\mathrm{j,k}}^{(\mathrm{i})}E[(v - \mu )_\mathrm{j}(v - \mu )_\mathrm{k}],$$

where we took the expectation inside the sum and the matrix elements outside the expectation as they do not involve *v*. For j≠k, the two terms inside the expectation involving *v* are independent given our distributional assumptions on *v* and the expectation can be taken separately. Using again the fact that *E*[*v*−*μ*] = 0, the sum equals zero for j≠k. It follows

$$\mathop {\sum }\limits_{\mathrm{j,k} = 1}^G H_{\mathrm{j,k}}^{(\mathrm{i})}E[(v - \mu )_\mathrm{j}(v - \mu )_\mathrm{k}] = \mathop {\sum}\limits_\mathrm{j} {H_{\mathrm{j,j}}^{(\mathrm{i})}E[(v_\mathrm{j} - \mu _\mathrm{j})^2]} = \mathop {\sum}\limits_\mathrm{j} {H_{\mathrm{j,j}}^{(\mathrm{i})}{{{\mathrm{Var}}}}[v_\mathrm{j}]} .$$

To summarize, our second-order approximation to the transition probabilities given the distribution in *v* reads

$$E[h_{\mathrm{i}}(v)] \approx h_{\mathrm{i}}(\mu ) + \frac{1}{2}\mathop {\sum }\limits_{\mathrm{j}} H_{\mathrm{j,j}}^{({\mathrm{i}})}\,{{{\mathrm{Var}}}}[v_{\mathrm{j}}].$$

We use automatic differentiation as implemented in JAX^96^ to compute the Hessian matrices *H*^(i)^, which ensures they are highly accurate and can be computed in a scalable manner. Further, because we do not hard-code the derivatives, our approach is highly flexible to future changes in the way we compute transition probabilities. If, for example, it turns out at a later point that an alternative metric works better than Pearson correlation, this is automatically taken care of in the propagation of uncertainties and no changes need to be made, apart from changing the forwards function that computes the transition probabilities themselves. The above procedure can be repeated for all components *i* and for all cells to yield the second-order approximation to the expected transition matrix *T*, given the distribution over each velocity vector.

### Approximating the expected final quantities

To arrive at the final quantities of interest, that is, state assignments and absorption probabilities, we use the expected transition matrix and proceed as in the deterministic case. We validate that this approximation gives very similar results to a fully stochastic approach based on MC sampling (Extended Data Fig. 3f,g). The MC approach is also available through our kernel interface by setting mode = ‘sampling’ when calling the method to compute the transition matrix. Thus, the user can conveniently choose between a fast approximate method given by our analytical approximation and a slower, asymptotically exact, method given by MC sampling.

#### The CellRank software package

The CellRank software package implements two main modules:

- kernels provide functionality to compute transition matrices based on (directed) single-cell data.
- estimators implement algorithms to perform inference based on kernels. For example, estimators compute macrostates and fate probabilities.

This modular and object-oriented design allows CellRank to be extended easily in two directions. Including more kernels can accommodate further directional single-cell data such as metabolic labeling or experimental time, while including more estimators enables learning new abstractions of cellular dynamics. The kernel module currently implements a

- VelocityKernel, which computes a transition matrix on the basis of a KNN graph and RNA velocity information.
- ConnectivityKernel, which row-normalizes the adjacency matrix underlying the KNN graph to obtain a valid transition matrix. This is essentially the transition matrix used in the diffusion pseudotime (DPT)^7^ algorithm.
- PrecomputedKernel, which accepts any precomputed transition matrix and allows for easy interfacing with the CellRank software.

All kernel classes are derived from a base kernel class that implements density normalization as implemented in Haghverdi et al.^7^. Instances of kernel classes can be combined by simply using the ‘+’ operator, potentially including weights. A typical code snippet to compute a transition matrix will look like this:

**Table:** 

from cellrank.tools.kernels import VelocityKernel, ConnectivityKernel
vk = VelocityKernel(adata).compute_transition_matrix()
ck = ConnectivityKernel(adata).compute_transition_matrix()
combined_kernel = 0.9*vk + 0.1*ck

The estimator module currently implements a

- CFLARE estimator. CFLARE (Clustering and Filtering of Left and Right Eigenvectors) computes terminal states directly by filtering cells in the top left eigenvectors and clustering them in the top right eigenvectors, thereby combining ideas of spectral clustering and stationary distributions.
- GPCCA estimator.

All estimator classes are derived from a base estimator class that enables computing fate probabilities, regardless of how terminal/intermediate states have been computed. A typical code snippet to compute macrostates and fate probabilities is:

**Table:** 

from cellrank.tools.estimators import GPCCA
# initialize the estimator
gpcca = GPCCA(combined_kernel)
# compute macrostates and identify the terminal states among them
gpcca.compute_macrostates()
gpcca.compute_terminal_states()
# compute fate probabilities
gpcca.compute_absorption_probabilities()

Both kernels and estimators implement a number of plotting functions to conveniently inspect results. We designed CellRank to be highly scalable to ever increasing cell numbers, widely applicable and extendable to problems in single-cell dynamical inference, user friendly with tutorials and comprehensive documentation, and robust with large code coverage through unit tests. CellRank is open source, fully integrated with SCANPY and scVelo and freely available at https://cellrank.org.

### Computing gene expression trends along lineages

CellRank computes fate probabilities that specify how likely each cell is to transition towards each identified terminal state (Computing fate probabilities). Combined with any pseudotemporal measure, this allows us to compute and compare gene expression trends towards specific terminal populations. In contrast to other methods like FateID^51^ or PAGA^8^, we do not define each lineage via a discrete assignment of cells obtained through a threshold or a clustering. Instead, we use all cells to fit each lineage, but we weight each cell according to its fate probability—our measure of lineage membership. Uncommitted cells can thus contribute to two or more fates, weighted by fate probabilities, while committed cells will be naturally excluded from alternative fates by virtue of fate probabilities nearing zero in these lineages.

### Pseudotemporal orderings

CellRank itself does not compute a pseudotemporal ordering of cells, as there are many established algorithms for this task, including DPT^7^, scVelo’s latent time^15^ or Palantir’s pseudotime^21^. A weak spot of these methods is that they rely on an initial cell to anchor their pseudotemporal ordering, whereas CellRank is the only method that can computationally identify initial states. Pseudotemporal orderings can be fed into CellRank, where we combine it with fate probabilities to compute gene expression trends along lineages. As mentioned above, fate probabilities are essential to make the gene expression trends specific to any particular lineage, by weighting each cell according to its contribution to that lineage.

### Imputing gene expression recovers trends from noisy data

To improve the robustness and resolution of gene expression trends, we adapt two strategies: imputed gene expression values and GAMs. For gene expression imputation, we use MAGIC^67^ by default; however, any imputed gene expression matrix can be supplied. MAGIC is based on KNN imputation and makes use of the covariance structure among neighboring cells to estimate expression levels for each gene. The KNN graph is computed globally, based on the expression values of all genes and not just the one we are currently considering.

### GAMs robustly fit gene expression values

Sliding window approaches are sensitive to local density differences and take only the current gene into account when determining gene expression trends. In contrast, we fit GAMs to expression values that have been imputed by borrowing information from neighboring cells via a KNN graph, allowing us to flexibly model many kinds of gene trends in a robust and scalable manner. We fit the expression trend for lineage t (associated with terminal state t) in gene *g* via

4 $$y_{\mathrm{g}i} = \beta _0 + f(\tau _\mathrm{i})\,\forall \mathrm{i}:F_{\mathrm{it}} > 0,$$

where *y*_gi_ is gene expression of gene g in cell i, *τ*_i_ is the pseudo temporal value of cell i and *F* is the fate matrix (Computing fate probabilities). By default, we use cubic splines for the smoothing functions *f* as these have been shown to be effective in capturing nonlinear relationships in trends^97^.

To visualize the smooth trend, we select 200 equally spaced testing points along pseudotime, and predict gene expression at each of them using the fitted model of equation (4). To estimate uncertainty along the trend, we use the s.d. of the residuals of the fit, given by

$$\sigma _{\hat y_\mathrm{p}} = \sqrt {\frac{{\mathop {\sum }\nolimits_{\mathrm{j} = 1}^n (y_\mathrm{j} - \widehat {y_\mathrm{j}})^2}}{{n - 2}}} \sqrt {1 + \frac{1}{n} + \frac{{(\tau _\mathrm{p} - \bar \tau )^2}}{{\mathop {\sum }\nolimits_{j = 1}^n (\tau _\mathrm{j} - \bar \tau )^2}}} ,$$

where $\hat y_\mathrm{p}$ denotes predicted gene expression at test point p, $\bar \tau$ denotes average pseudotime across all cells and *n* is the number of test points^98^. For the fitting of equation (4), we provide interfaces to both the R package mgcv^99,100^ as well as the Python package pyGAM^101^. We parallelize gene fitting to scale well in the number of genes, which is important when plotting heatmaps summarizing many gene expression trends.

### Visualizing gene expression trends for the pancreas example

For CellRank’s gene expression trends of lineage-associated genes along the alpha, beta, epsilon and delta fates, we used Palantir’s pseudotime^21^, MAGIC imputed data^67^ and the mgcv^99,100^ package to fit GAMs in a cubic spline basis. For the delta lineage, fate probabilities among early cells were very low (0.01 average fate probability among Ngn3 high EP cells; Fig. 2e). This reflects the small size of the delta population (70 cells or 3% of the data; Supplementary Fig. 10a,b) as well as the fact that delta cells are produced mostly at later stages in pancreatic development^37^. To still be able to reliably fit gene expression of early cells along the delta lineage, we clip weights below 0.01 to this threshold value. This was done only for the fitting of gene expression trends.

### Clustering gene expression trends

CellRank can cluster gene expression trends along a particular lineage to recover the main patterns of (transient) up- or downregulation towards a specific terminal state. We recover regulation of our gene set of interest along a specific lineage by fitting GAMs in pseudotime, supplying fate probabilities as cell-level lineage weights. Next, we cluster the GAM-smoothed gene expression trends. For this, we z-transform expression values and compute a principal component analysis (PCA) representation of the trends. By default, we use 50 PCs. We then compute a KNN graph in PC space and cluster the KNN graph using the louvain^68^ or leiden^102^ algorithms. For each recovered cluster, we compute its mean and s.d. (pointwise, for all testing points that were used for smoothing) and visualize them, together with the individual, smoothed trends per cluster. As gene-trend fitting is efficiently parallelized in CellRank, such an analysis can be performed in an unbiased fashion for large gene sets. For 10,000 genes, the runtime is about 6 min on a 2019 Macbook pro with a 2.8 GHz Intel Core i7 processor and 16 GB RAM.

### Clustering gene expression trends towards the delta fate

To cluster gene expression trends towards the delta fate in Fig. 3e, all genes expressed in at least ten cells were included (12,987 genes). Smooth gene expression trends along the delta lineage were determined using Palantir’s pseudotime^21^. We used *K* = 30 for the gene-trend KNN graph and the Louvain algorithm with resolution parameter set to 0.2 to avoid overclustering the trends.

### Uncovering putative driver genes

To find genes that are expressed at high levels in cells that are biased towards a particular fate, we compute Person’s correlation between expression levels of a set of genes and fate probabilities. We sort genes by their correlation values and consider high-scoring genes as candidate drivers. By default, we consider all genes that have passed preprocessing gene-filtering thresholds. The computation of correlation values can be restricted to a set of predefined clusters if one is interested in driver genes that act in a certain region of the phenotypic manifold.

### Uncovering putative driver genes for delta development

To uncover putative driver genes towards the delta fate in Fig. 3d,e, we considered 12,987 genes that were expressed in at least ten cells. We computed correlation of total-count normalized, log-transformed gene expression values with the probability of becoming a delta cell. We restricted correlation computation to the Fev+ cluster, where we expected the fate decision towards delta to occur.

### Robustness analysis

We were interested in evaluating how much CellRank’s fate probabilities change in response to changes in the following key preprocessing parameters:

- Weight given to transcriptomic similarities via the *λ* parameter;
- Number of neighbors *K* used for KNN graph construction;
- scVelo’s gene-filtering parameter "minsharedcounts", which determines how many counts a gene must have in both spliced and unspliced layers;
- scVelo’s gene-filtering parameter "ntopgenes", which determines the number of most highly variable genes used for the velocity computation;
- Number of PCs "npcs" used for KNN graph construction.

In addition, we were interested to see how much CellRank’s results change when we randomly subsample the number of cells to 90% of the original cell number and when we vary the number of macrostates. We used the pancreas example^24^ in all of the following comparisons.

### Robustness with respect to key preprocessing parameters

To evaluate robustness to preprocessing parameters, we varied only one parameter at a time and computed macrostates and their associated fate probabilities. We then compared fate probabilities for different parameter values by computing pairwise Pearson correlation among all possible pairs of parameter values. We did this separately for the alpha, beta, epsilon and delta lineages. For each lineage, we recorded the median and minimum correlation achieved across all the different comparisons. We always computed enough macrostates so that the alpha, beta, epsilon and delta states were included. Naturally, the precise location of the terminal states changed slightly across parameter combinations. For this reason, the correlation values we recorded reflect robustness of the entire CellRank workflow, including both the computation of terminal states as well as fate probabilities. In a separate comparison, we were interested in evaluating the robustness of just the last step of the CellRank algorithm, that is the computation of fate probabilities. For this, we kept the terminal states fixed across parameter variations and proceeded as above otherwise, computing pairwise Pearson correlations among fate probabilities per lineage across all parameter value combinations. To test whether CellRank’s robustness changes when we propagate uncertainty, we repeated all the aforementioned computations using our analytical approximation to propagate uncertainty.

### Statistical testing of increased robustness due to uncertainty propagation

To check whether propagating uncertainty increased robustness with respect to *K* used during neighborhood graph construction, we fixed the terminal states and computed pairwise correlations with and without uncertainty propagation. This yielded ten correlation values per lineage per method. We then applied a one-sided Wilcoxon signed-rank test separately for each lineage using the scipy^89^ implementation with an exact distribution for the test statistic. This test assumes paired data, and that each pair is drawn independently. Pairs in our case are given by correlations of fate probabilities for two different numbers of neighbors, *K*, computed with and without uncertainty propagation. We assume these to be paired as the same number of neighbors probably yields similar correlation values with and without uncertainty propagation. For the alpha, beta and epsilon lineages, this yielded the same test statistic because the signed ranks of the differences in correlation between uncertainty/no uncertainty propagation were the same, that is, uncertainty propagation always yielded higher correlation values and the one-sided Wilcoxon signed-rank test does not consider the actual magnitude of the differences, but just their sign and rank.

### Robustness with respect to random subsampling of cells

We subsampled the data to 90% of cells, computed macrostates and fate probabilities towards the alpha, beta, epsilon and delta states. We repeated this 20 times, recorded all computed fate probabilities and compared them pairwise per lineage using Pearson’s correlation for all possible pairs of random draws. As in the above evaluation for the key preprocessing parameters, we recorded minimum and median correlation per lineage across all pairs and we repeated this for fixed terminal states and for propagated uncertainty.

### Robustness with respect to the number of macrostates

To evaluate sensitivity with respect to this parameter, we varied the number of macrostates between 10 and 16 and confirmed that, inside this range, the key terminal and initial states exist and remain in the same location.

### Pancreas data example

We used an scRNA-seq time-series dataset comprising embryonic days 12.5−15.5 of pancreatic development in mice assayed using 10x Genomics^24^. We restricted the data to the last timepoint (E15.5) and to the Ngn3 low EP, Ngn3 high EP, Fev+ and endocrine clusters to focus on the late stages of endocrinogenesis where all of alpha, beta, epsilon and delta fates are present. For the main analysis of Figs. 2 and 3, we filtered out cycling cells to amplify the differentiation signal. In Extended Data Fig. 6, we include these cycling populations and show that convoluted signals of differentiation and proliferation pose no problem to CellRank. Our final subset for Figs. 2 and 3 contained 2,531 cells. We kept the original cluster annotations, which were available on a coarse level and on a fine level. On the fine level, the Fev+ cluster was subclustered into different populations biased towards different endocrine fates (Fig. 3a).

### Data preprocessing and velocity computation for the pancreas example

We used scVelo^15^ and SCANPY^76^ with mostly default parameters. Loom files containing raw spliced and unspliced counts were obtained by running the velocyto^14^ command-line pipeline. We filtered genes to be expressed in at least ten cells and to have at least 20 counts in both spliced and unspliced layers. We further normalized by total counts per cell, log-transformed the data and kept the top 2,000 highly variable genes. We then computed a PCA representation of the data and used the top 30 PCs to compute a KNN graph with *K* = 30. For velocity computation, we used scVelo’s dynamic model of splicing kinetics. We evaluate the robustness of CellRank’s results to changes in these preprocessing parameters (Robustness analysis).

### Embedding computation for the pancreas example

We used the KNN graph to compute a PAGA^8^ representation of the data. The PAGA graph was used to initialize the computation of a UMAP^28^ representation of the data. Note that UMAP was used only to visualize the data and was not supplied to CellRank to compute the transition matrix or any downstream quantities.

### CellRank parameters for the pancreas example

We used CellRank’s analytical stochastic approximation to compute transition probabilities and included a diffusion kernel with weight 0.2. We computed 12 macrostates and automatically detected the terminal alpha, beta and epsilon states. The delta population was picked up automatically as a macrostate. We manually assigned it the terminal label.

### Statistical testing of Fev+ subcluster delta fate bias

To check whether Fev+ delta cells were assigned significantly higher delta fate probability compared with other Fev+ clusters by CellRank, we applied a two-sided Welch unequal variances *t*-test. The test assumes two independent normally distributed samples with unequal variances and checks whether their means are significantly different.

### Comparing fate probabilities with observed cell-type frequencies

The pancreas system involves the nonhomeostatic generation of endocrine cells, and is thus not in steady state. In such a setting, we do not expect fate probabilities to perfectly follow observed cell-type frequencies, as different populations are produced at different developmental stages. For example, while 19% of cells in the pancreas data are terminal alpha, it is well known that these have been produced at earlier stages of endocrinogenesis (around E12.5) and not at E15.5 (ref. ^37^). However, these earlier alpha cells still exist at E15.5 and contribute to observed cell-type frequencies. It is a strength of CellRank that it correctly picks this up (Fig. 2e), and does not assign large fate probabilities for differentiating towards alpha cells at E15.5.

### Lung data example

We used an scRNA-seq time-series dataset of lung regeneration following bleomycin injury in mice assayed using Drop-seq^52,53^. It contains 18 timepoints comprising days 0–54 postinjury, with daily sampling from days 2–13 and wider lags between subsequent timepoints. Two replicate mice were used per timepoint. We restricted data to days 2–15 to ensure dense sampling. If timepoints are too far apart, RNA velocity cannot be used to predict the next likely cellular state because linear extrapolation is meaningful only on the time scales of the splicing kinetics. Our final subset contained 24,882 cells. We kept the original cluster annotations.

### Data preprocessing and velocity computation for the lung example

We used scVelo and SCANPY with mostly default parameters. Loom files containing raw spliced and unspliced counts were obtained by running the velocyto^14^ command-line pipeline. We filtered genes to be expressed in at least ten cells and to have at least 20 counts in both spliced and unspliced layers. We further normalized by total counts per cell, log-transformed the data and kept the top 2,000 highly variable genes. We kept the PCA coordinates from the original study and computed a KNN graph with *K* = 30 using the top 50 PCs. For velocity computation, we used scVelo’s dynamical model of splicing kinetics.

### Embedding computation for the lung example

The lung data was processed in three separate batches. We used BBKNN^103^ to compute a batch corrected KNN graph with ten neighbors in each batch. The corrected KNN graph was used to compute a UMAP representation of the data. Note that UMAP was used only to visualize the data and was not supplied to CellRank to compute the transition matrix or any downstream quantities. We did not use BBKNN to correct the graph we used for velocity computation as it is an open question how to do batch correction for velocity computation. We used uncorrected data for velocity computation.

### CellRank parameters for the lung example

We used the analytical stochastic approximation of CellRank to compute transition probabilities and included a diffusion kernel with weight 0.2. On the full data of Fig. 6a, we computed nine macrostates. On the reduced data of Supplementary Fig. 16a, we computed three macrostates.

### Defining stages of the differentiation trajectory

We reran CellRank on the subset of goblet and basal cells to investigate the trajectory at higher resolution. CellRank automatically detected initial and terminal states and computed fate probabilities towards the terminal states (Supplementary Fig. 16a–c). Further, we applied Palantir to the subset to compute a pseudotime (Supplementary Fig. 16d,e). We combined pseudotime with CellRank’s fate probabilities to define three stages of the dedifferentiation trajectory by requiring cells to have at least 0.66 basal probability. Cells passing this threshold were assigned to three bins of equal size along the pseudotemporal axis. We used this binning to define the three stages of the trajectory.

### Reprogramming data example

We used an scRNA-seq time-series dataset of MEFs in vitro reprogramming towards iEPs^48^ across a 28-day time course of retroviral overexpression of *Foxa1* and *Hnf4a*^49^. The original dataset contains 104,887 cells assayed using 10x and Drop-seq^53^. We analyzed the subset of 48,515 cells shown in figure 3 of Biddy et al.^49^, which is enriched for later stages of reprogramming and contains only cells assayed using 10x. We kept the original cluster annotations, the original 2D t-SNE embedding to visualize the data and the CellTag-derived successful versus dead-end labels from Biddy et al.^49^.

### Data preprocessing and velocity computation for the reprogramming example

We used scVelo^15^ and SCANPY^76^ with mostly default parameters. Loom files containing raw spliced and unspliced counts were obtained by running the velocyto^14^ command-line pipeline. We filtered genes to have at least 20 counts in both spliced and unspliced layers. We further normalized by total counts per cell, log-transformed the data and kept the top 2,000 highly variable genes. We computed a 30 nearest neighbor graph in the top 30 PCs and ran scVelo’s dynamical model of splicing kinetics to compute velocities.

### CellRank parameters for the reprogramming example

We use CellRank’s analytical stochastic approximation to compute transition probabilities and include a diffusion kernel with weight 0.2. We computed five macrostates.

### Comparing CellRank fate probabilities with CellTag labels

We sought to compare CellRank-computed fate probabilities towards the successful and dead-end terminal states (Fig. 4c) to CellTag-derived ground truth labels from the original publication^49^ via a classification task. Ground truth labels were binary (successful/dead-end) and available for a subset of all cells. We restricted the comparison to days 12, 15 and 21 where ground truth labels were available for 374, 582 and 1,312 cells, respectively. More ground truth labels were available for dead-end than for successful cells, which can give rise to misleading classification accuracy. We therefore subsampled dead-end cells until the proportions were even. For classification, we randomly assigned 60% of labeled cells per day into the training set and the remaining cells into the test set. Our final cell sets contained 208 (124 training/84 testing), 308 (184 training/124 testing) and 652 (391 training/261 testing) cells for days 12, 15 and 21, respectively. We trained logistic regression classifiers independently for each day to predict the ground truth success/dead-end labels based on CellRanks fate probabilities on the training set using the scikit-learn implementation^104^. To assess predictive performance, we computed receiver operating characteristic (ROC) curves for each day on the test set. In short, ROC curves are created by iterating over the decision threshold used to classify points as successful/dead-end, computing and plotting the true positive rate against the false positive rate for each decision threshold^105^. For each day, we also compute the area under the ROC curve (AUC). The AUC is a measure between 0 and 1 to summarize the entire ROC curve into a single value, which is then threshold-independent. A value of 1 corresponds to perfect classification, 0.5 corresponds to random guessing, that is, an uninformative classifier.

### Immunofluorescence stainings and microscopy on airway epithelial cells

Formalin-fixed paraffin-embedded lung sections (3.5 μm thick) from bleomycin-treated mice at day 10 (*n* = 2) and day 22 (*n* = 2) after bleomycin instillation, and from PBS-treated controls (*n* = 2) were stained as previously described^52^. In brief, after deparaffinization, rehydration and heat-mediated antigen retrieval with citrate buffer (10 mM, pH 6.0), sections were blocked with 5% bovine serum albumin for 1 h at room temperature and then incubated with the following primary antibodies overnight at 4 °C: rabbit anti-Bpifb1 (kindly provided by C. Bingle^106^, 1:500), mouse anti-Trp63 (abcam, catalog no. ab735, clone A4A, 1:50) and chicken anti-Krt5 (BioLegend, catalog no. Poly9059, 1:1,000).

The following secondary antibodies were used: Goat anti-rabbit Alexa Fluor 488 (Invitrogen, catalog no. A11008, 1:250), Goat anti-chicken Alexa Fluor 568 (Invitrogen, catalog no. A11041,1:250) and goat anti-mouse Alexa Fluor 647 (Invitrogen, catalog no. A21236, 1:250). Nuclei were visualized with 4′,6-diamidino-2-phenylindole.

Immunofluorescent images were acquired with an AxioImager.M2 microscope (Zeiss) using a Plan-Apochromat ×20/0.8 M27 objective. For quantification of immunofluorescence staining, five different intrapulmonary regions were recorded per mouse, and the percentage of positively stained cells normalized to the total number of airway cells was quantified manually using Fiji software (ImageJ, v.2.0.0).

### Ethics statement

Pathogen-free 8- to 10-week-old female C57BL/6J mice were purchased from Charles River Germany and maintained at the appropriate biosafety level at constant temperature (20–24 °C) and humidity (45–65%) with a 12 h light cycle. Animals were allowed food and water ad libitum. All animal experiments were performed in accordance with the governmental and international guidelines and ethical oversight by the local government for the administrative region of Upper Bavaria (Germany), registered under 55.2-1-54-2532-130-2014 and ROB-55.2-2532.Vet_02-16-208.

### Reporting Summary

Further information on research design is available in the Nature Research Reporting Summary linked to this article.

## Online content

Any methods, additional references, Nature Research reporting summaries, source data, extended data, supplementary information, acknowledgements, peer review information; details of author contributions and competing interests; and statements of data and code availability are available at 10.1038/s41592-021-01346-6.

## Supplementary information

Supplementary Information Supplementary Figs. 1–17, Tables 1–3 and Note 1–2.

Reporting Summary

## Data Availability

Raw published data for the pancreas^24^, lung^52^ and reprogramming^49^ examples are available from the Gene Expression Omnibus under accession codes GSE132188, GSE141259 and GSE99915, respectively. Processed data, including spliced and unspliced count abundances, is available from figshare under 10.6084/m9.figshare.c.5172299.

## Code availability

The CellRank software package is available at https://cellrank.org including documentation, tutorials and examples. Jupyter notebooks to reproduce our analysis and figures are available at https://github.com/theislab/cellrank_reproducibility.

## References

[1] Bendall, S. C. et al. Single-cell trajectory detection uncovers progression and regulatory coordination in human B cell development. *Cell* **157**, 714–725 (2014). 24766814 10.1016/j.cell.2014.04.005 PMC4045247

[2] Baron, C. S. & van Oudenaarden, A. Unravelling cellular relationships during development and regeneration using genetic lineage tracing. *Nat. Rev. Mol. Cell Biol.* **20**, 753–765 (2019). 31690888 10.1038/s41580-019-0186-3

[3] Wagner, D. E. & Klein, A. M. Lineage tracing meets single-cell omics: opportunities and challenges. *Nat. Rev. Genet.* **21**, 410–427 (2020). 32235876 10.1038/s41576-020-0223-2 PMC7307462

[4] Erhard, F. et al. scSLAM-seq reveals core features of transcription dynamics in single cells. *Nature* **571**, 419–423 (2019). 31292545 10.1038/s41586-019-1369-y

[5] Battich, N. et al. Sequencing metabolically labeled transcripts in single cells reveals mRNA turnover strategies. *Science* **367**, 1151–1156 (2020). 32139547 10.1126/science.aax3072

[6] Qiu, Q. et al. Massively parallel and time-resolved RNA sequencing in single cells with scNT-seq. *Nat. Methods* **17**, 991–1001 (2020). 32868927 10.1038/s41592-020-0935-4 PMC8103797

[7] Haghverdi, L., Büttner, M., Wolf, F. A., Buettner, F. & Theis, F. J. Diffusion pseudotime robustly reconstructs lineage branching. *Nat. Methods* **13**, 845–848 (2016). 27571553 10.1038/nmeth.3971

[8] Wolf, F. A. et al. PAGA: graph abstraction reconciles clustering with trajectory inference through a topology preserving map of single cells. *Genome Biol.* **20**, 59 (2019). 30890159 10.1186/s13059-019-1663-x PMC6425583

[9] Setty, M. et al. Wishbone identifies bifurcating developmental trajectories from single-cell data. *Nat. Biotechnol.* **34**, 637–645 (2016). 27136076 10.1038/nbt.3569 PMC4900897

[10] Qiu, X. et al. Reversed graph embedding resolves complex single-cell trajectories. *Nat. Methods* **14**, 979–982 (2017). 28825705 10.1038/nmeth.4402 PMC5764547

[11] Saelens, W., Cannoodt, R., Todorov, H. & Saeys, Y. A comparison of single-cell trajectory inference methods. *Nat. Biotechnol.* **37**, 547–554 (2019). 30936559 10.1038/s41587-019-0071-9

[12] Trapnell, C. et al. The dynamics and regulators of cell fate decisions are revealed by pseudotemporal ordering of single cells. *Nat. Biotechnol.* **32**, 381–386 (2014). 24658644 10.1038/nbt.2859 PMC4122333

[13] Weinreb, C., Wolock, S., Tusi, B. K., Socolovsky, M. & Klein, A. M. Fundamental limits on dynamic inference from single-cell snapshots. *Proc. Natl Acad. Sci. USA* **115**, E2467–E2476 (2018). 29463712 10.1073/pnas.1714723115 PMC5878004

[14] La Manno, G. et al. RNA velocity of single cells. *Nature* **560**, 494–498 (2018). 30089906 10.1038/s41586-018-0414-6 PMC6130801

[15] Bergen, V., Lange, M., Peidli, S., Wolf, F. A. & Theis, F. J. Generalizing RNA velocity to transient cell states through dynamical modeling. *Nat. Biotechnol.* **38**, 1408–1414 (2020). 32747759 10.1038/s41587-020-0591-3

[16] Gorin, G., Svensson, V. & Pachter, L. Protein velocity and acceleration from single-cell multiomics experiments. *Genome Biol.* **21**, 39 (2020). 32070398 10.1186/s13059-020-1945-3 PMC7029606

[17] Schiebinger, G. et al. Optimal-transport analysis of single-cell gene expression identifies developmental trajectories in reprogramming. *Cell* **176**, 1517 (2019). 30849376 10.1016/j.cell.2019.02.026 PMC6615720

[18] Fischer, D. S. et al. Inferring population dynamics from single-cell RNA-sequencing time series data. *Nat. Biotechnol.* **37**, 461–468 (2019). 30936567 10.1038/s41587-019-0088-0 PMC7397487

[19] Reuter, B., Fackeldey, K. & Weber, M. Generalized Markov modeling of nonreversible molecular kinetics. *J. Chem. Phys.* **150**, 174103 (2019). 31067901 10.1063/1.5064530

[20] Reuter, B., Weber, M., Fackeldey, K., Röblitz, S. & Garcia, M. E. Generalized Markov state modeling method for nonequilibrium biomolecular dynamics: exemplified on amyloid β conformational dynamics driven by an oscillating electric field. *J. Chem. Theory Comput.* **14**, 3579–3594 (2018). 29812922 10.1021/acs.jctc.8b00079

[21] Setty, M. et al. Characterization of cell fate probabilities in single-cell data with Palantir. *Nat. Biotechnol.* **37**, 451–460 (2019). 30899105 10.1038/s41587-019-0068-4 PMC7549125

[22] Buenrostro, J. D. et al. Integrated single-cell analysis maps the continuous regulatory landscape of human hematopoietic differentiation. *Cell* **173**, 1535–1548.e16 (2018). 29706549 10.1016/j.cell.2018.03.074 PMC5989727

[23] Stergachis, A. B. et al. Developmental fate and cellular maturity encoded in human regulatory DNA landscapes. *Cell* **154**, 888–903 (2013). 23953118 10.1016/j.cell.2013.07.020 PMC3962256

[24] Bastidas-Ponce, A. et al. Comprehensive single cell mRNA profiling reveals a detailed roadmap for pancreatic endocrinogenesis. *Development* **146**, dev. 173849 (2019). 10.1242/dev.173849 31160421

[25] Becht, E. et al. Dimensionality reduction for visualizing single-cell data using UMAP. *Nat. Biotechnol.* **37**, 38–44 (2019). 10.1038/nbt.4314 30531897

[26] Maaten, Lvander & Hinton, G. Visualizing data using t-SNE. *J. Mach. Learn. Res.* **9**, 2579–2605 (2008).

[27] Amir, E.-A. D. et al. viSNE enables visualization of high dimensional single-cell data and reveals phenotypic heterogeneity of leukemia. *Nat. Biotechnol.* **31**, 545–552 (2013). 23685480 10.1038/nbt.2594 PMC4076922

[28] McInnes, L., Healy, J. & Melville, J. UMAP: uniform manifold approximation and projection for dimension reduction. Preprint at *arXiv* https://arxiv.org/abs/1802.03426 (2018).

[29] Heiser, C. N. & Lau, K. S. A quantitative framework for evaluating single-cell data structure preservation by dimensionality reduction techniques. *Cell Rep.* **31**, 107576 (2020). 32375029 10.1016/j.celrep.2020.107576 PMC7305633

[30] Kobak, D. & Linderman, G. C. Initialization is critical for preserving global data structure in both t-SNE and UMAP. *Nat. Biotechnol.* **39**, 156–157 (2021). 33526945 10.1038/s41587-020-00809-z

[31] Cooley, S. M., Hamilton, T., Deeds, E. J. & Ray, J. C. J. A novel metric reveals previously unrecognized distortion in dimensionality reduction of scRNA-Seq data. Preprint at *bioRxiv* https://www.biorxiv.org/content/10.1101/689851v1 (2019).

[32] Luecken, M. D. & Theis, F. J. Current best practices in single-cell RNA-seq analysis: a tutorial. *Mol. Syst. Biol.* **15**, e8746 (2019). 31217225 10.15252/msb.20188746 PMC6582955

[33] Bastidas-Ponce, A., Scheibner, K., Lickert, H. & Bakhti, M. Cellular and molecular mechanisms coordinating pancreas development. *Development* **144**, 2873–2888 (2017). 28811309 10.1242/dev.140756

[34] Bastidas-Ponce, A. et al. Foxa2 and Pdx1 cooperatively regulate postnatal maturation of pancreatic β-cells. *Mol. Metab.* **6**, 524–534 (2017). 28580283 10.1016/j.molmet.2017.03.007 PMC5444078

[35] Zhang, J., McKenna, L. B., Bogue, C. W. & Kaestner, K. H. The diabetes gene Hhex maintains δ-cell differentiation and islet function. *Genes Dev.* **28**, 829–834 (2014). 24736842 10.1101/gad.235499.113 PMC4003275

[36] Krentz, N. A. J. et al. Single-cell transcriptome profiling of mouse and hESC-derived pancreatic progenitors. *Stem Cell Rep.* **11**, 1551–1564 (2018). 10.1016/j.stemcr.2018.11.008 PMC6294286 30540962

[37] Johansson, K. A. et al. Temporal control of neurogenin3 activity in pancreas progenitors reveals competence windows for the generation of different endocrine cell types. *Dev. Cell* **12**, 457–465 (2007). 17336910 10.1016/j.devcel.2007.02.010

[38] Berthault, C., Staels, W. & Scharfmann, R. Purification of pancreatic endocrine subsets reveals increased iron metabolism in beta cells. *Mol. Metab.* **42**, 101060 (2020). 32763423 10.1016/j.molmet.2020.101060 PMC7498953

[39] Cram, D. S., McIntosh, A., Oxbrow, L., Johnston, A. M. & DeAizpurua, H. J. Differential mRNA display analysis of two related but functionally distinct rat insulinoma (RIN) cell lines: identification of CD24 and its expression in the developing pancreas. *Differentiation* **64**, 237–246 (1999). 10365441 10.1046/j.1432-0436.1999.6440237.x

[40] Lee, K. et al. FOXA2 is required for enhancer priming during pancreatic differentiation. *Cell Rep.* **28**, 382–393.e7 (2019). 31291575 10.1016/j.celrep.2019.06.034 PMC6636862

[41] Ahlgren, U., Pfaff, S. L., Jessell, T. M., Edlund, T. & Edlund, H. Independent requirement for ISL1 in formation of pancreatic mesenchyme and islet cells. *Nature* **385**, 257–260 (1997). 9000074 10.1038/385257a0

[42] Hiesberger, T. et al. Mutation of hepatocyte nuclear factor-1beta inhibits Pkhd1 gene expression and produces renal cysts in mice. *J. Clin. Invest.* **113**, 814–825 (2004). 15067314 10.1172/JCI20083 PMC362119

[43] Haumaitre, C. et al. Lack of TCF2/vHNF1 in mice leads to pancreas agenesis. *Proc. Natl Acad. Sci. USA* **102**, 1490–1495 (2005). 15668393 10.1073/pnas.0405776102 PMC547822

[44] Kawase, S. et al. Regulatory factor X transcription factors control Musashi1 transcription in mouse neural stem/progenitor cells. *Stem Cells Dev.* **23**, 2250–2261 (2014). 25058468 10.1089/scd.2014.0219 PMC4155420

[45] Emery, P., Durand, B., Mach, B. & Reith, W. RFX proteins, a novel family of DNA binding proteins conserved in the eukaryotic kingdom. *Nucleic Acids Res.* **24**, 803–807 (1996). 8600444 10.1093/nar/24.5.803 PMC145730

[46] Ait-Lounis, A. et al. The transcription factor Rfx3 regulates beta-cell differentiation, function, and glucokinase expression. *Diabetes* **59**, 1674–1685 (2010). 20413507 10.2337/db09-0986 PMC2889767

[47] Smith, S. B. et al. Rfx6 directs islet formation and insulin production in mice and humans. *Nature* **463**, 775–780 (2010). 20148032 10.1038/nature08748 PMC2896718

[48] Morris, S. A. et al. Dissecting engineered cell types and enhancing cell fate conversion via CellNet. *Cell* **158**, 889–902 (2014). 25126792 10.1016/j.cell.2014.07.021 PMC4291075

[49] Biddy, B. A. et al. Single-cell mapping of lineage and identity in direct reprogramming. *Nature* **564**, 219–224 (2018). 30518857 10.1038/s41586-018-0744-4 PMC6635140

[50] Velten, L. et al. Human haematopoietic stem cell lineage commitment is a continuous process. *Nat. Cell Biol.* **19**, 271–281 (2017). 28319093 10.1038/ncb3493 PMC5496982

[51] Herman, J. S., Sagar & Grün, D. FateID infers cell fate bias in multipotent progenitors from single-cell RNA-seq data. *Nat. Methods* **15**, 379–386 (2018). 29630061 10.1038/nmeth.4662

[52] Strunz, M. et al. Alveolar regeneration through a Krt8 transitional stem cell state that persists in human lung fibrosis. *Nat. Commun.* **11**, 3559 (2020). 32678092 10.1038/s41467-020-17358-3 PMC7366678

[53] Macosko, E. Z. et al. Highly parallel genome-wide expression profiling of individual cells using nanoliter droplets. *Cell* **161**, 1202–1214 (2015). 26000488 10.1016/j.cell.2015.05.002 PMC4481139

[54] Tata, P. R. et al. Dedifferentiation of committed epithelial cells into stem cells in vivo. *Nature* **503**, 218–223 (2013). 24196716 10.1038/nature12777 PMC4035230

[55] Tetteh, P. W., Farin, H. F. & Clevers, H. Plasticity within stem cell hierarchies in mammalian epithelia. *Trends Cell Biol.* **25**, 100–108 (2015). 25308311 10.1016/j.tcb.2014.09.003

[56] Weinberger, S. E., Cockrill, B. A. & Mandel, J. *Principles of Pulmonary Medicine*. (Saunders/Elsevier, 2008).

[57] Hogan, B. L. M. et al. Repair and regeneration of the respiratory system: complexity, plasticity, and mechanisms of lung stem cell function. *Cell Stem Cell* **15**, 123–138 (2014). 25105578 10.1016/j.stem.2014.07.012 PMC4212493

[58] Rock, J. R., Randell, S. H. & Hogan, B. L. M. Airway basal stem cells: a perspective on their roles in epithelial homeostasis and remodeling. *Dis. Model. Mech.* **3**, 545–556 (2010). 20699479 10.1242/dmm.006031 PMC2931533

[59] Qiu, X. *et al*. Mapping transcriptomic vector fields of single cells. Preprint at *bioRxiv*10.1101/696724 (2021).

[60] Kimmel, J. C., Yi, N., Roy, M., Hendrickson, D. G. & Kelley, D. R. Differentiation reveals latent features of aging and an energy barrier in murine myogenesis. *Cell Rep.* **35**, 109046 (2021). 33910007 10.1016/j.celrep.2021.109046

[61] Weinreb, C., Rodriguez-Fraticelli, A., Camargo, F. D. & Klein, A. M. Lineage tracing on transcriptional landscapes links state to fate during differentiation. *Science* **367**, eaaw3381 (2020). 31974159 10.1126/science.aaw3381 PMC7608074

[62] Ranzoni, A. M. et al. Integrative single-cell RNA-seq and ATAC-seq analysis of human developmental hematopoiesis. *Cell Stem Cell* **28**, 472–487.e7 (2021). 33352111 10.1016/j.stem.2020.11.015 PMC7939551

[63] Van den Berge, K. et al. Trajectory-based differential expression analysis for single-cell sequencing data. *Nat. Commun.* **11**, 1201 (2020). 32139671 10.1038/s41467-020-14766-3 PMC7058077

[64] Nowotschin, S. et al. The emergent landscape of the mouse gut endoderm at single-cell resolution. *Nature* **569**, 361–367 (2019). 30959515 10.1038/s41586-019-1127-1 PMC6724221

[65] Forrow, A. & Schiebinger, G. LineageOT is a unified framework for lineage tracing and trajectory inference. *Nat. Commun.* **12**, 4940 (2021). 34400634 10.1038/s41467-021-25133-1 PMC8367995

[66] Cannoodt, R., Saelens, W., Deconinck, L. & Saeys, Y. Spearheading future omics analyses using dyngen, a multi-modal simulator of single cells. *Nat. Commun.* **12**, 3942 (2021). 34168133 10.1038/s41467-021-24152-2 PMC8225657

[67] van Dijk, D. et al. Recovering gene interactions from single-cell data using data diffusion. *Cell* **174**, 716–729.e27 (2018). 29961576 10.1016/j.cell.2018.05.061 PMC6771278

[68] Blondel, V. D., Guillaume, J.-L., Lambiotte, R. & Lefebvre, E. Fast unfolding of communities in large networks. *J. Stat. Mech.* **2008**, P10008 (2008).

[69] Sekiya, S. & Suzuki, A. Direct conversion of mouse fibroblasts to hepatocyte-like cells by defined factors. *Nature* **475**, 390–393 (2011). 21716291 10.1038/nature10263

[70] Stoffers, D. A., Zinkin, N. T., Stanojevic, V., Clarke, W. L. & Habener, J. F. Pancreatic agenesis attributable to a single nucleotide deletion in the human IPF1 gene coding sequence. *Nat. Genet.* **15**, 106–110 (1997). 8988180 10.1038/ng0197-106

[71] Jonsson, J., Carlsson, L., Edlund, T. & Edlund, H. Insulin-promoter-factor 1 is required for pancreas development in mice. *Nature* **371**, 606–609 (1994). 7935793 10.1038/371606a0

[72] Jaitin, D. A. et al. Massively parallel single-cell RNA-seq for marker-free decomposition of tissues into cell types. *Science* **343**, 776–779 (2014). 24531970 10.1126/science.1247651 PMC4412462

[73] Hendriks, G.-J. et al. NASC-seq monitors RNA synthesis in single cells. *Nat. Commun.* **10**, 3138 (2019). 31316066 10.1038/s41467-019-11028-9 PMC6637240

[74] Röblitz, S. & Weber, M. Fuzzy spectral clustering by PCCA+: application to Markov state models and data classification. *Adv. Data Anal. Classif.* **7**, 147–179 (2013).

[75] Spivak, D. I. Metric realization of fuzzy simplicial sets. *Self published notes* https://math.mit.edu/~dspivak/files/metric_realization.pdf (2012).

[76] Wolf, F. A., Angerer, P. & Theis, F. J. SCANPY: large-scale single-cell gene expression data analysis. *Genome Biol.* **19**, 15 (2018). 29409532 10.1186/s13059-017-1382-0 PMC5802054

[77] Coifman, R. R. et al. Geometric diffusions as a tool for harmonic analysis and structure definition of data: diffusion maps. *Proc. Natl Acad. Sci. USA* **102**, 7426–7431 (2005). 15899970 10.1073/pnas.0500334102 PMC1140422

[78] Soneson, C., Srivastava, A., Patro, R. & Stadler, M. B. Preprocessing choices affect RNA velocity results for droplet scRNA-seq data. *PLoS Comput. Biol.* **17**, e1008585 (2021). 33428615 10.1371/journal.pcbi.1008585 PMC7822509

[79] Raj, B. & Blencowe, B. J. Alternative splicing in the mammalian nervous system: recent insights into mechanisms and functional roles. *Neuron* **87**, 14–27 (2015). 26139367 10.1016/j.neuron.2015.05.004

[80] Martinez, N. M. & Lynch, K. W. Control of alternative splicing in immune responses: many regulators, many predictions, much still to learn. *Immunol. Rev.* **253**, 216–236 (2013). 23550649 10.1111/imr.12047 PMC3621013

[81] Pan, Q., Shai, O., Lee, L. J., Frey, B. J. & Blencowe, B. J. Deep surveying of alternative splicing complexity in the human transcriptome by high-throughput sequencing. *Nat. Genet.* **40**, 1413–1415 (2008). 18978789 10.1038/ng.259

[82] Wang, E. T. et al. Alternative isoform regulation in human tissue transcriptomes. *Nature* **456**, 470–476 (2008). 18978772 10.1038/nature07509 PMC2593745

[83] Reuter, B. *Generalisierte Markov-Modellierung: Modellierung Irreversibler β-Amyloid-Peptid-Dynamik unter Mikrowelleneinfluss* (Springer, 2020).

[84] Mucha, H.-J. *Big Data Clustering: Data Preprocessing, Variable Selection and Dimension Reduction* (WIAS, 2017).

[85] Golub, G. H. & Van Loan, C. F. *Matrix Computations* (JHU Press, 2013).

[86] Horn, R. A. & Johnson, C. R. *Matrix Analysis* (Cambridge Univ. Press, 2012).

[87] Kube, S. & Weber, M. A coarse graining method for the identification of transition rates between molecular conformations. *J. Chem. Phys.* **126**, 024103 (2007). 17228939 10.1063/1.2404953

[88] Weber, M. *Meshless Methods in Conformation Dynamics*. PhD Thesis, Freie Univ., Berlin (2006).

[89] Virtanen, P. et al. SciPy 1.0: fundamental algorithms for scientific computing in Python. *Nat. Methods* **17**, 261–272 (2020). 32015543 10.1038/s41592-019-0686-2 PMC7056644

[90] Reuter, B., Weber, M., Fackeldey, K., Röblitz, S. & Garcia, M. E. Generalized Markov state modeling method for nonequilibrium biomolecular dynamics: exemplified on amyloid β conformational dynamics driven by an oscillating electric field. *J. Chem. Theory Comput.* **14**, 3579–3594 (2018). 29812922 10.1021/acs.jctc.8b00079

[91] Reuter, B. *pyGPCCA: pyGPCCA - python GPCCA: Generalized Perron Cluster Cluster Analysis package to coarse-grain reversible and non-reversible Markov State Models*. (Github) https://github.com/msmdev/pyGPCCA

[92] Hernandez, V., Roman, J. E. & Vidal, V. SLEPc: a scalable and flexible toolkit for the solution of eigenvalue problems. *ACM Trans. Math. Softw.* **31**, 351–362 (2005).

[93] Dalcin, L. D., Paz, R. R., Kler, P. A. & Cosimo, A. Parallel distributed computing using Python. *Adv. Water Resour.* **34**, 1124–1139 (2011).

[94] Tolver, A. *An introduction to Markov chains*. (*Univ. of Copenhagen*, 2016).

[95] Saad, Y. & Schultz, M. H. GMRES: a generalized minimal residual algorithm for solving nonsymmetric linear systems. *SIAM J. Sci. Stat. Comput.* **7**, 856–869 (1986).

[96] Frostig, R., Johnson, M. & Leary, C. Compiling machine learning programs via high-level tracing. In *Proc. SYSML’18, February 2018, Stanford, CA USA* https://cs.stanford.edu/~rfrostig/pubs/jax-mlsys2018.pdf (2018).

[97] Hastie, T. & Tibshirani, R. Generalized additive models. *SSO Schweiz. Monatsschr. Zahnheilkd.* **1**, 297–310 (1986).

[98] DeSalvo, J. S. *Standard error of forecast in multiple regression: proof of a useful result*. (Rand Corporation, 1970).

[99] Mgcv: Mixed GAM computation vehicle with automatic smoothness estimation. (R Foundation for Statistical Computing, 2019) https://CRAN.R-project.org/package=mgcv

[100] Wood, S. N. *Generalized Additive Models: An Introduction with R*. (CRC Press/Taylor & Francis Group, 2017).

[101] Servén, D., Brummitt, C. pyGAM: Generalized additive models in Python. Preprint at *Zenodo*10.5281/zenodo.1476122 (2018).

[102] Traag, V. A., Waltman, L. & van Eck, N. J. From Louvain to Leiden: guaranteeing well-connected communities. *Sci. Rep.* **9**, 5233 (2019). 30914743 10.1038/s41598-019-41695-z PMC6435756

[103] Polański, K. et al. BBKNN: fast batch alignment of single cell transcriptomes. *Bioinformatics* **36**, 964–965 (2020). 31400197 10.1093/bioinformatics/btz625 PMC9883685

[104] Pedregosa, F. et al. Scikit-learn: machine learning in Python. *J. Mach. Learn. Res.* **12**, 2825–2830 (2011).

[105] Fawcett, T. An introduction to ROC analysis. *Pattern Recognit. Lett.* **27**, 861–874 (2006).

[106] Musa, M. et al. Differential localisation of BPIFA1 (SPLUNC1) and BPIFB1 (LPLUNC1) in the nasal and oral cavities of mice. *Cell Tissue Res.* **350**, 455–464 (2012). 22986921 10.1007/s00441-012-1490-9 PMC3505551

[107] Tirosh, I. et al. Dissecting the multicellular ecosystem of metastatic melanoma by single-cell RNA-seq. *Science* **352**, 189–196 (2016). 27124452 10.1126/science.aad0501 PMC4944528

[108] Byrnes, L. E. et al. Lineage dynamics of murine pancreatic development at single-cell resolution. *Nat. Commun.* **9**, 3922 (2018). 30254276 10.1038/s41467-018-06176-3 PMC6156586

