# Supplementary material for: CellRank for directed single-cell fate mapping
Source: Nat Methods. 2022 Jan 13;19(2):159–70. doi: 10.1038/s41592-021-01346-6 (PMC8828480; doi:10.1038/s41592-021-01346-6)
Supplement: Supplementary file 1 — Supplementary Figs. 1–17, Tables 1–3 and Note 1–2. [file 41592_2021_1346_MOESM1_ESM.pdf]

nature portfolio

https://doi.org/10.1038/s41592-021-01346-6

Supplementary information

CellRank for directed single-cell fate mapping

In the format provided by the authors and unedited

Supplementary Figures

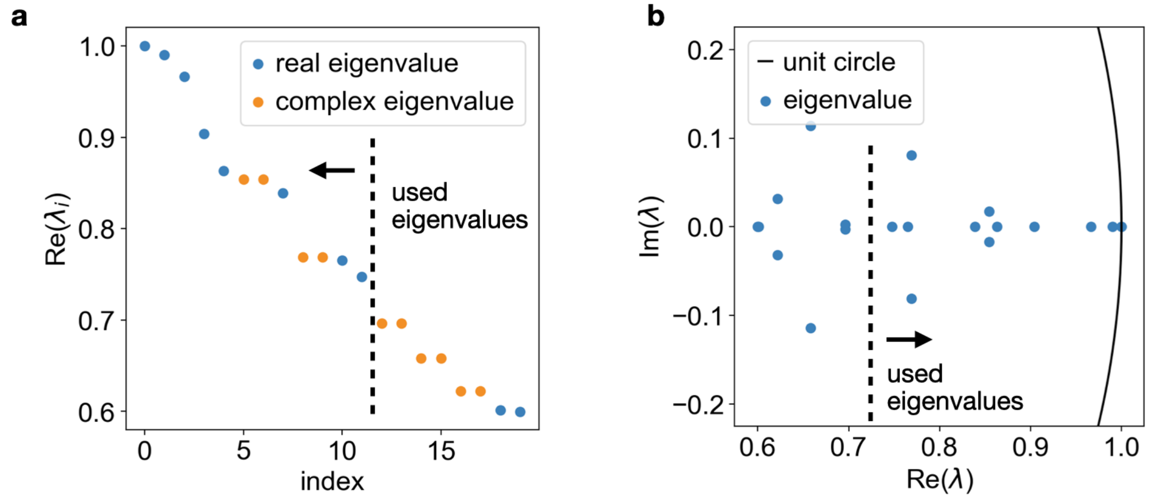

Suppl. Fig. 1 | Spectrum of the pancreas transition matrix

a. Real part of the top 20 eigenvalues. Purely real eigenvalues are shown in blue. Complex eigenvalues come in pairs of complex conjugates for real matrices and are shown in orange. Dashed line highlights the first 12 eigenvalues, which we use to compute macrostates in Fig. 2b. **b.** Eigenvalues from (**a**) in the complex plane.

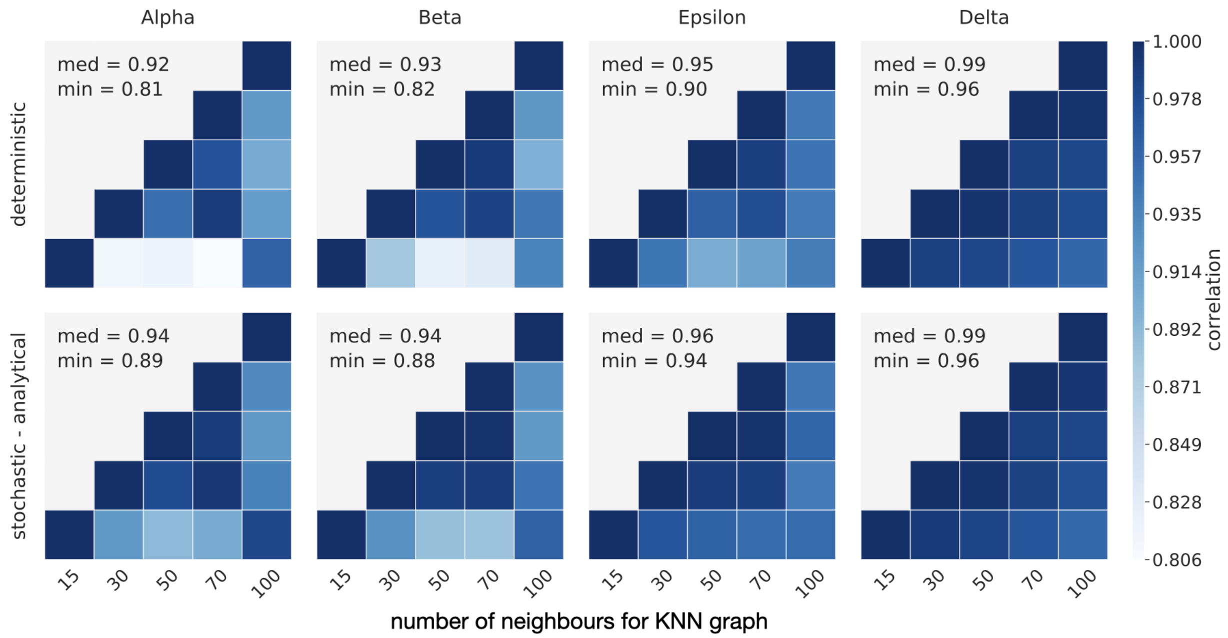

Suppl. Fig. 2 | Propagating uncertainty significantly increases robustness of fate probabilities

We show this here for the alpha, beta and epsilon lineages with respect to parameter changes (one-sided Wilcoxon signed-rank test, 10 correlation values per lineage, W = 55.0, P = 9.7 x 10^{-4}, Online Methods). For the delta lineage, no significant robustness increase was found. This plot shows variation in the number of neighbors during KNN graph construction (see Supplementary Fig. 8 and Supplementary Fig. 9 for more parameters).

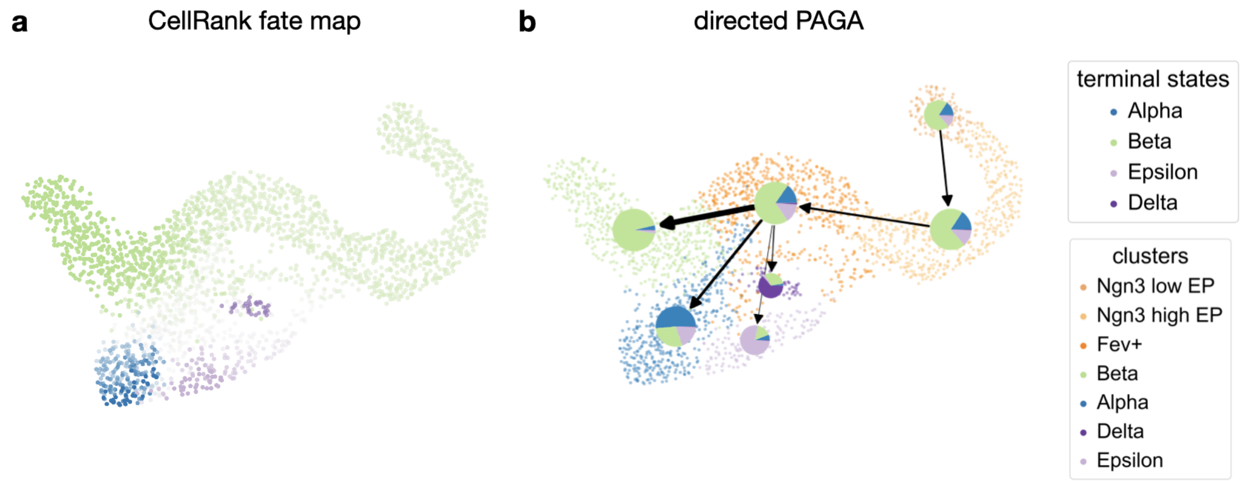

Suppl. Fig. 3 | Visualising fate probabilities in a new directed PAGA graph

a. Fate probabilities for the pancreas data from Fig. 2e, visualised as a fate map where each cell is colored according to its most likely fate. Color intensity reflects the degree of lineage priming. **b.** Probabilistic approximate graph abstraction (PAGA)[1] in a new directed layout, combined with CellRank's fate probabilities, shown as pie charts. Arrows represent aggregated velocity flow (Supplementary Note 1).

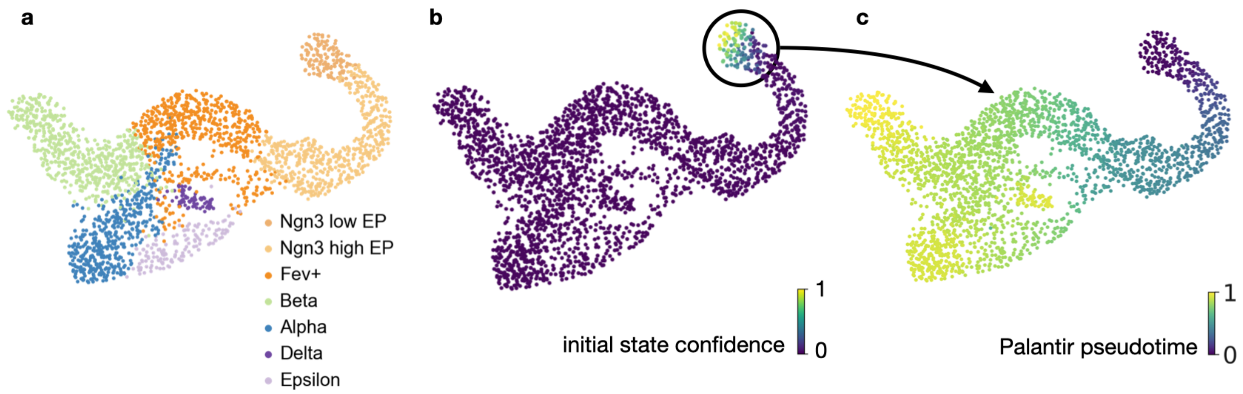

Suppl. Fig. 4 | Palantir pseudotime for the pancreas data

a. UMAP of the pancreas data of Fig. 2a with cluster annotations from the original publication[2]. **b.** Membership vector corresponding to the Ngn3^{low} EP_1 macrostate which we identified as an initial state. **c.** We selected one of the cells which had high initial state confidence in (**b**) and supplied it to Palantir to compute a pseudotemporal ordering of all cells[3].

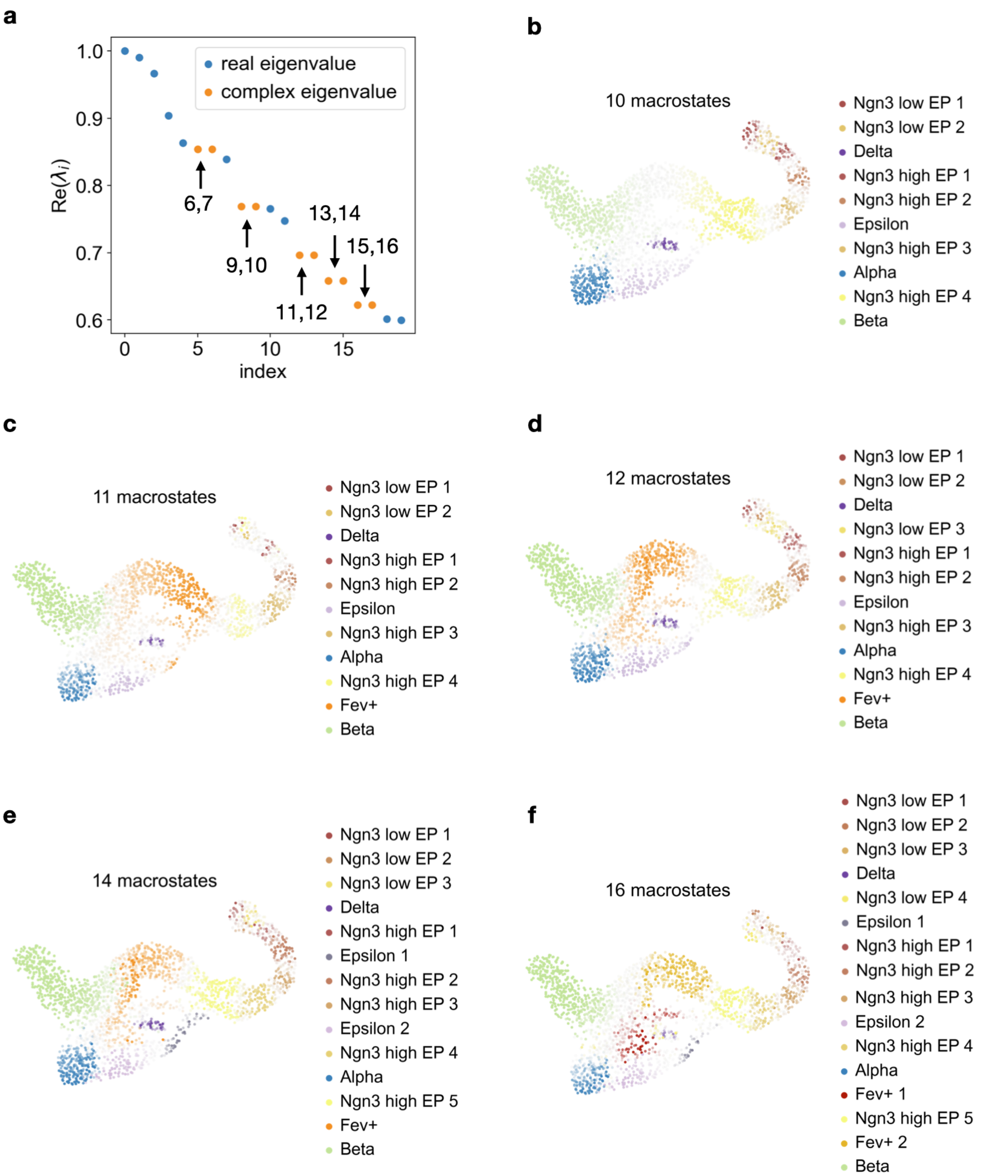

Suppl. Fig. 5 | Varying the number of macrostates for the pancreas does not change biological interpretation

a. Real part of the 20 eigenvalues with the largest real part for the pancreas transition matrix of Fig. 2. We highlight eigenvalues that come in pairs of complex conjugates. Splitting pairs of complex conjugates leads to non-invariant subspaces (Online Methods), therefore, we choose a number of states which always includes both eigenvalues from pairs of complex conjugates. **b-f.**

When varying the number of macrostates, we consistently recover the alpha, beta, epsilon, delta and Ngn3^{low} EP_1 states. Increasing the number of macrostates increases the resolution at which we interpret the data. However, for the findings we report in Fig. 2, any of the number of macrostates presented here would lead to similar results and near identical biological interpretation.

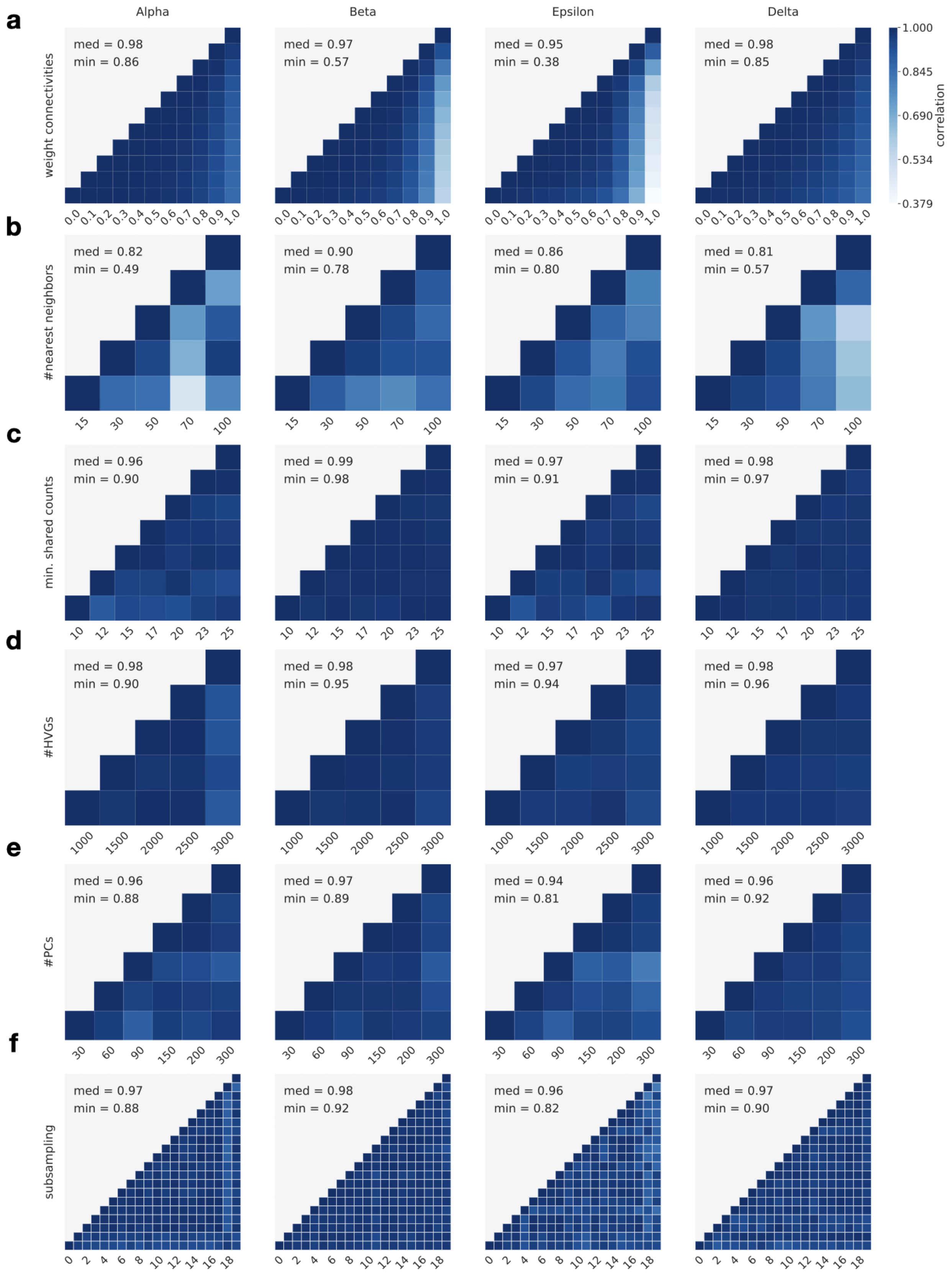

a
b
c
d
e
f
Alpha
Beta
Epsilon
Delta
weight connectivities
#nearest neighbors
min. shared counts
#HVGs
#PCs
subsampling
correlation
1.000
0.845
0.690
0.534
0.379
med = 0.98
min = 0.86
med = 0.97
min = 0.57
med = 0.95
min = 0.38
med = 0.98
min = 0.85
med = 0.82
min = 0.49
med = 0.90
min = 0.78
med = 0.86
min = 0.80
med = 0.81
min = 0.57
med = 0.96
min = 0.90
med = 0.99
min = 0.98
med = 0.97
min = 0.91
med = 0.98
min = 0.97
med = 0.98
min = 0.90
med = 0.98
min = 0.95
med = 0.97
min = 0.94
med = 0.98
min = 0.96
med = 0.96
min = 0.88
med = 0.97
min = 0.89
med = 0.94
min = 0.81
med = 0.96
min = 0.92
med = 0.97
min = 0.88
med = 0.98
min = 0.92
med = 0.96
min = 0.82
med = 0.97
min = 0.90
0.0 0.1 0.2 0.3 0.4 0.5 0.6 0.7 0.8 0.9 1.0
15 30 50 70 100
10 12 15 17 20 23 25
1000 1500 2000 2500 3000
30 60 90 150 200 300
0 2 4 6 8 10 12 14 16 18

Suppl. Fig. 6 | CellRank is robust to parameter choice and random subsampling

a-e Pairwise correlations of fate probabilities per lineage when varying (**a**) the weight given to transcriptomic similarity (lambda) (**b**) the number of nearest neighbors in KNN graph construction, (**c**) the gene filtering parameter “min_shared_counts” which determines the minimum required number of spliced and unspliced counts, (**d**) the number of highly variable genes, (**e**) the number of principal components used for KNN graph construction. Across the 4 parameters, we achieve a minimum median correlation of 0.81, highlighting CellRanks robustness to a wide range of parameter choices. **f.** Pairwise correlations of fate probabilities per lineage when randomly subsampling the data to 90% of cells. CellRank is very robust to subsampling with a minimum median correlation of 0.96.

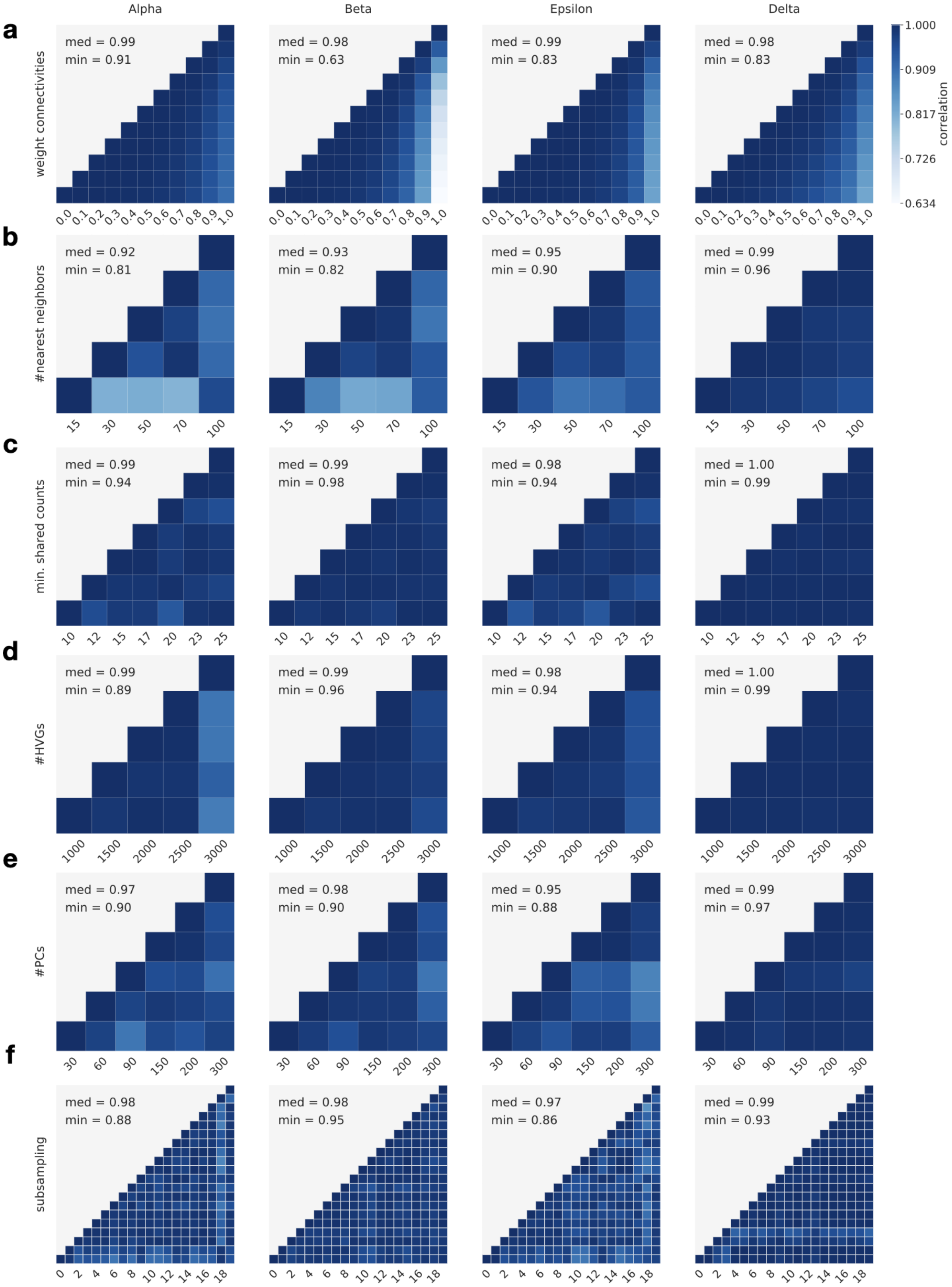
Alpha
Beta
Epsilon
Delta
a
weight connectivities
med = 0.99
min = 0.91
med = 0.98
min = 0.63
med = 0.99
min = 0.83
med = 0.98
min = 0.83
0.0 0.1 0.2 0.3 0.4 0.5 0.6 0.7 0.8 0.9 1.0
1.000
0.909
0.817
0.726
0.634
correlation
b
#nearest neighbors
med = 0.92
min = 0.81
med = 0.93
min = 0.82
med = 0.95
min = 0.90
med = 0.99
min = 0.96
15 30 50 70 100
c
min. shared counts
med = 0.99
min = 0.94
med = 0.99
min = 0.98
med = 0.98
min = 0.94
med = 1.00
min = 0.99
10 12 15 17 20 23 25
d
#HVGs
med = 0.99
min = 0.89
med = 0.99
min = 0.96
med = 0.98
min = 0.94
med = 1.00
min = 0.99
1000 1500 2000 2500 3000
e
#PCs
med = 0.97
min = 0.90
med = 0.98
min = 0.90
med = 0.95
min = 0.88
med = 0.99
min = 0.97
30 60 90 150 200 300
f
subsampling
med = 0.98
min = 0.88
med = 0.98
min = 0.95
med = 0.97
min = 0.86
med = 0.99
min = 0.93
0 2 4 6 8 10 12 14 16 18

Suppl. Fig. 7 | Fixing the terminal states further increases robustness

a-f Like Supplementary Fig. 6, only that we fix the terminal states to restrict the robustness comparison to the computation of fate probabilities, i.e. the terminal states have been computed once and were fixed across all parameter variations and subsampling of cells. Note that the color scale changed. This increases the minimum median correlation for parameter variations (**a-d**) from 0.81 with recomputed terminal states to 0.92 here and for subsampling (**f**) from 0.96 with recomputed terminal states to 0.97 here.

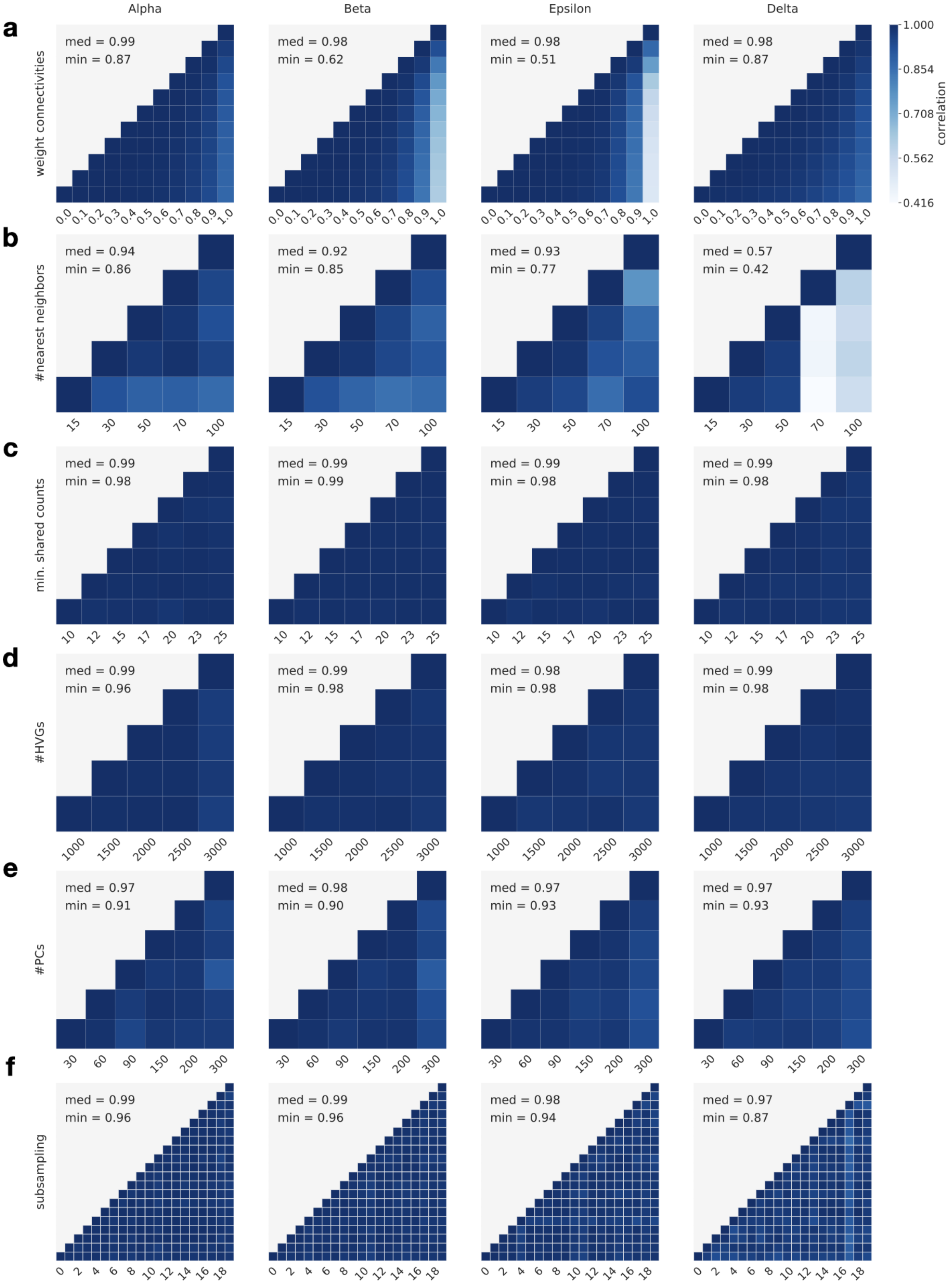

Alpha
Beta
Epsilon
Delta
a
weight connectivities
med = 0.99
min = 0.87
med = 0.98
min = 0.62
med = 0.98
min = 0.51
med = 0.98
min = 0.87
0.0 0.1 0.2 0.3 0.4 0.5 0.6 0.7 0.8 0.9 1.0
1.000
0.854
0.708
0.562
0.416
correlation
b
#nearest neighbors
med = 0.94
min = 0.86
med = 0.92
min = 0.85
med = 0.93
min = 0.77
med = 0.57
min = 0.42
15 30 50 70 100
c
min. shared counts
med = 0.99
min = 0.98
med = 0.99
min = 0.99
med = 0.99
min = 0.98
med = 0.99
min = 0.98
10 12 15 17 20 23 25
d
#HVGs
med = 0.99
min = 0.96
med = 0.99
min = 0.98
med = 0.98
min = 0.98
med = 0.99
min = 0.98
1000 1500 2000 2500 3000
e
#PCs
med = 0.97
min = 0.91
med = 0.98
min = 0.90
med = 0.97
min = 0.93
med = 0.97
min = 0.93
30 60 90 150 200 300
f
subsampling
med = 0.99
min = 0.96
med = 0.99
min = 0.96
med = 0.98
min = 0.94
med = 0.97
min = 0.87
0 2 4 6 8 10 12 14 16 18

Suppl. Fig. 8 | Robustness increases when propagating uncertainty

a-f. Like Supplementary Fig. 6, only that we use the analytical approximation to propagate uncertainty into the transition probabilities. Apart from the delta lineage for the number of nearest neighbors, this increases the minimum median correlation for parameter variations (**a-e)** from 0.81 in the deterministic case to 0.92 here. When we vary the number of nearest neighbors for the delta lineage, we observe outlier terminal states when using 70 nearest neighbors. This effect disappears when we fix the terminal states (Supplementary Fig. 9). **f.** For subsampling, using the stochastic approximation increases the minimum median correlation from 0.96 in the deterministic case to 0.97 here.

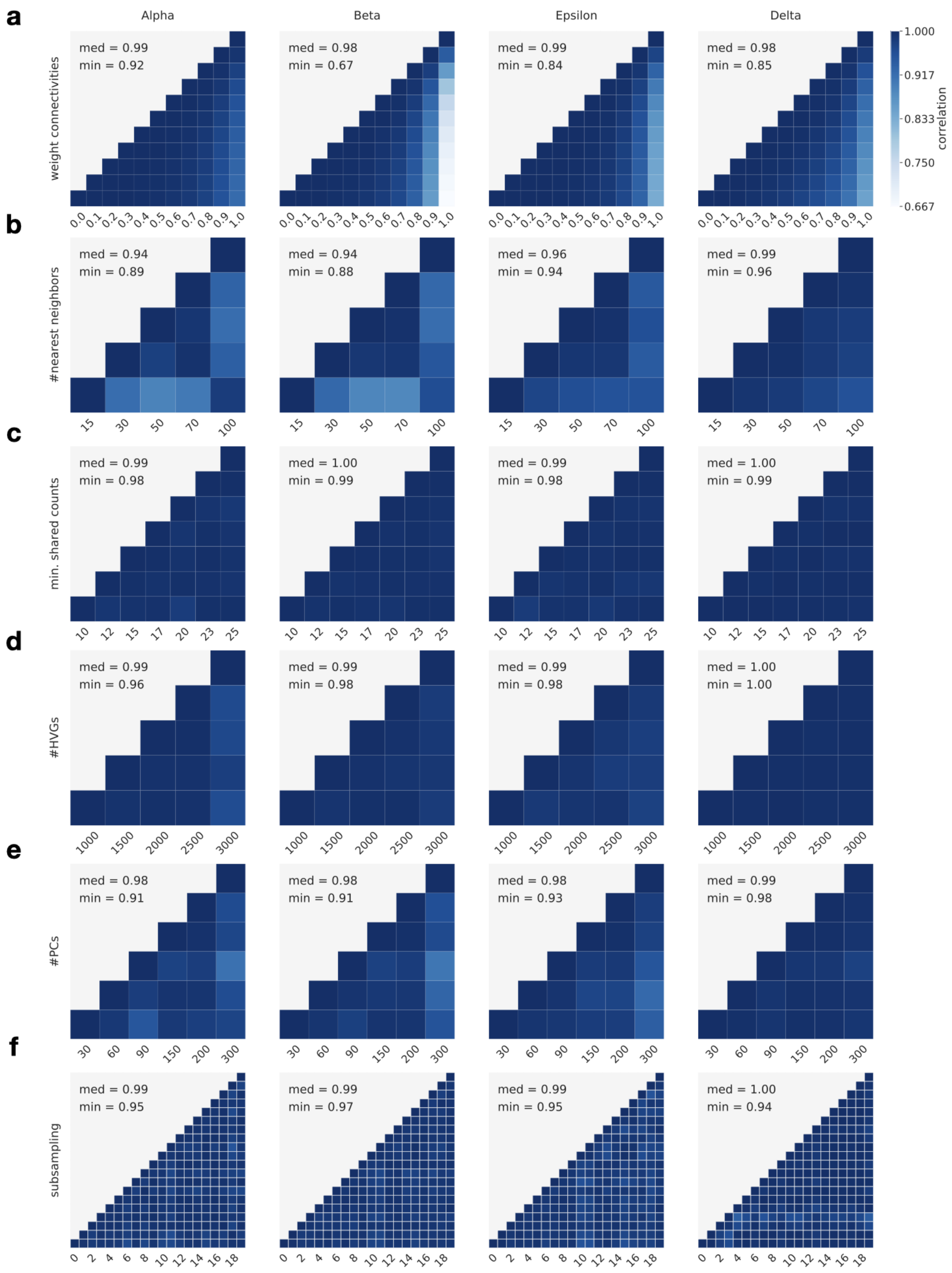
a
b
c
d
e
f
Alpha
Beta
Epsilon
Delta
weight connectivities
#nearest neighbors
min. shared counts
#HVGs
#PCs
subsampling
correlation
med = 0.99
min = 0.92
med = 0.98
min = 0.67
med = 0.99
min = 0.84
med = 0.98
min = 0.85
med = 0.94
min = 0.89
med = 0.94
min = 0.88
med = 0.96
min = 0.94
med = 0.99
min = 0.96
med = 0.99
min = 0.98
med = 1.00
min = 0.99
med = 0.99
min = 0.98
med = 1.00
min = 0.99
med = 0.99
min = 0.96
med = 0.99
min = 0.98
med = 0.99
min = 0.98
med = 1.00
min = 1.00
med = 0.98
min = 0.91
med = 0.98
min = 0.91
med = 0.98
min = 0.93
med = 0.99
min = 0.98
med = 0.99
min = 0.95
med = 0.99
min = 0.97
med = 0.99
min = 0.95
med = 1.00
min = 0.94

Suppl. Fig. 9 | Robustness further increases when propagating uncertainty and fixing the terminal states

a-f. Like Supplementary Fig. 8, only that we fix the terminal states to restrict the robustness comparison to the computation of fate probabilities, i.e. the terminal states have been computed once and were fixed across all parameter perturbations and subsampling of cells. This increases the minimum median correlation for parameter variations (**a-e)** from 0.57 with recomputed terminal states to 0.94 here and for subsampling (**f)** from 0.97 with recomputed terminal states to 0.99 here.

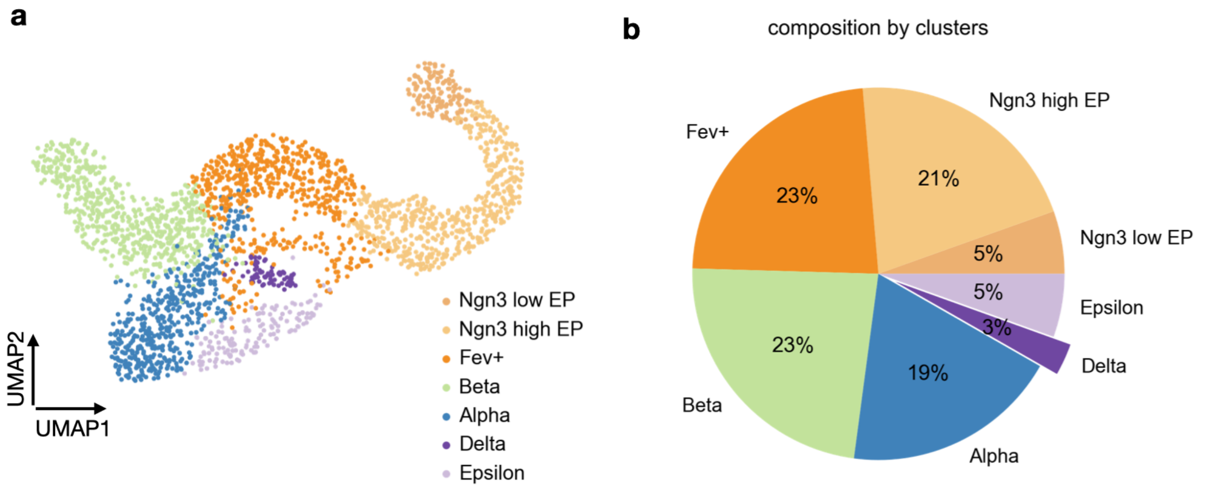

Suppl. Fig. 10 | Cell type proportions in the pancreas data

a. UMAP representation of the pancreas data from Fig. 2a with original cluster annotations[2]. **b.** Cell type proportions. Delta cells are the rarest cell type in this data with only 3% abundance.

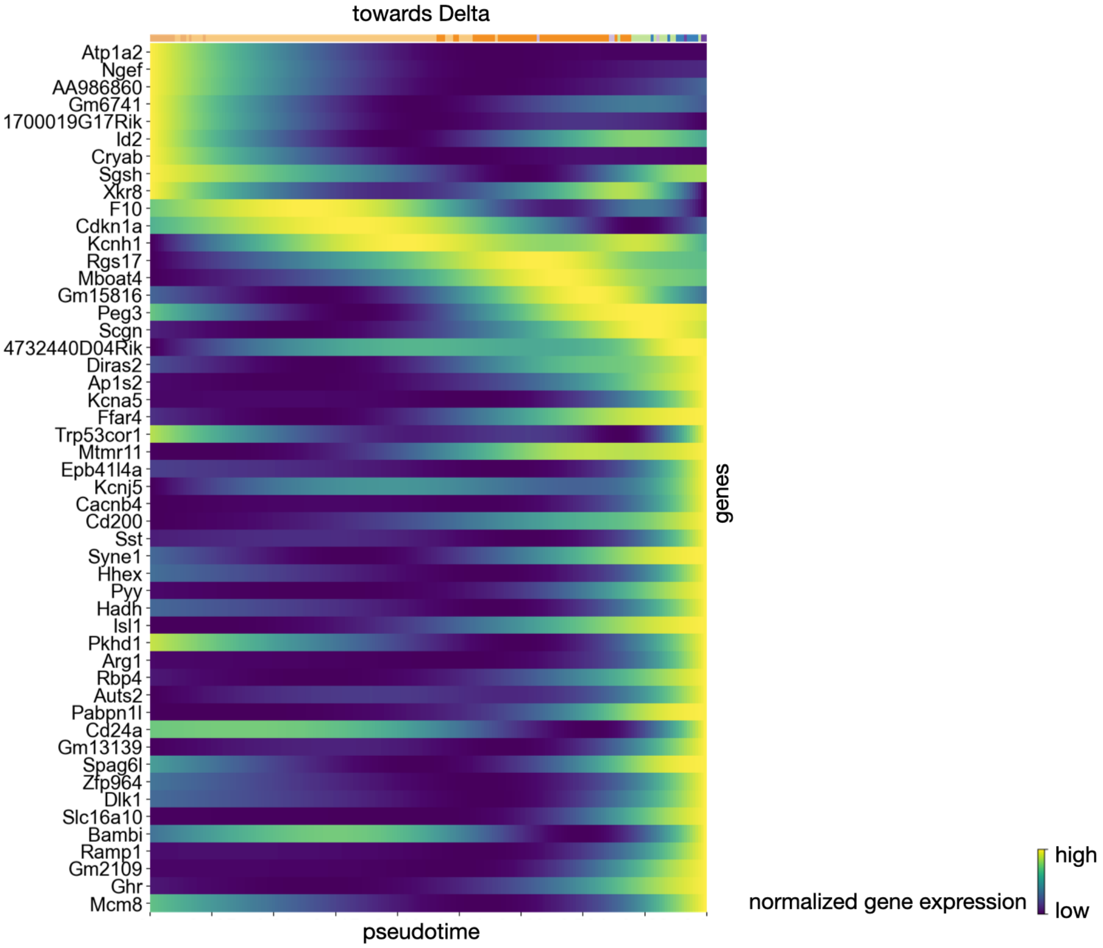

Suppl. Fig. 11 | Heatmap of genes whose expression correlates well with delta fate

Heatmap of Fig. 3d with all gene names shown.

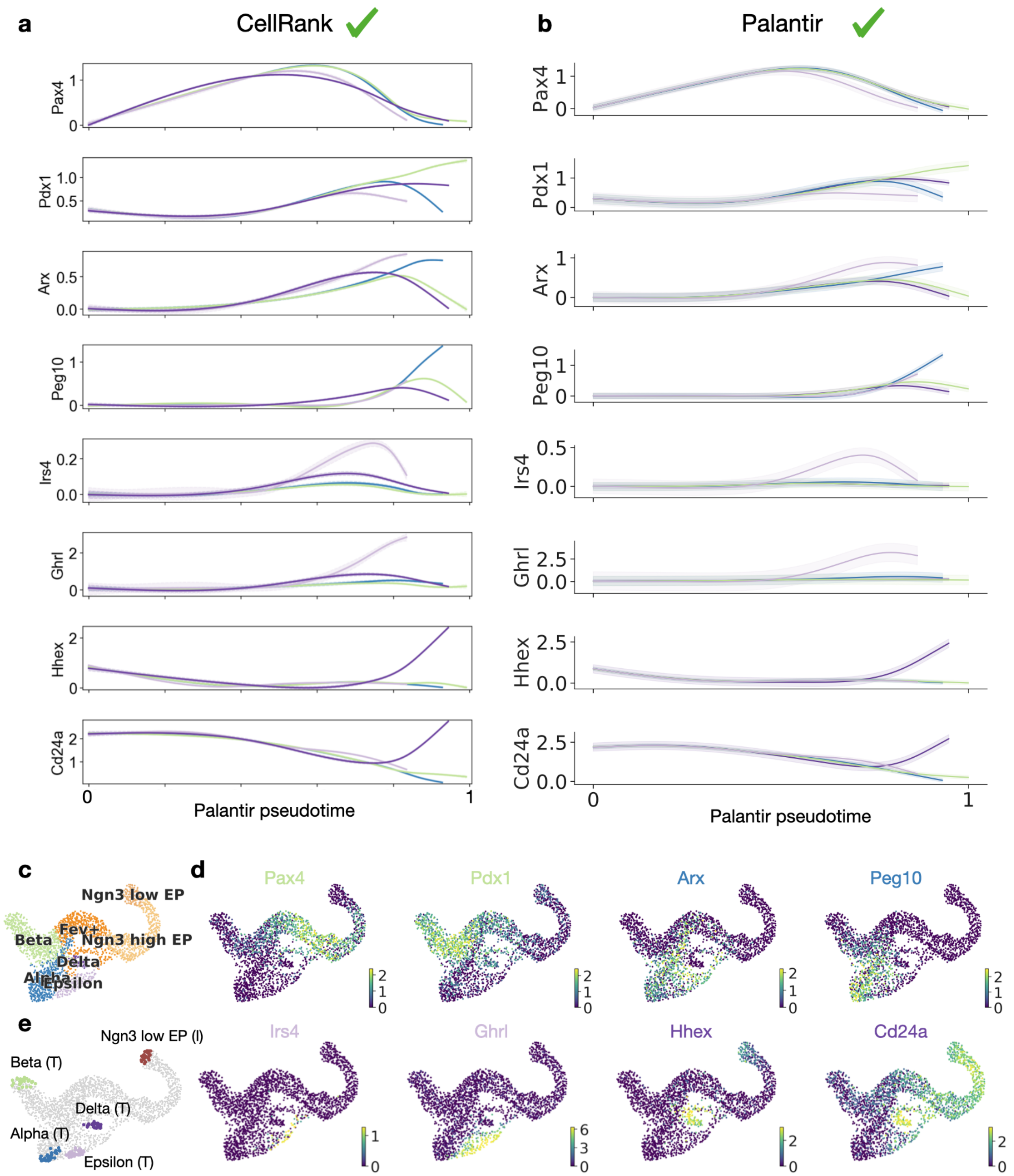

Suppl. Fig. 12 | Gene expression trends for CellRank and Palantir

a-b. Gene expression trends for key regulators *Pax4*[4] and *Pdx1*[5–7] (beta), *Arx*[4] (alpha), *Ghrl*[4] (epsilon), *Hhex*[8] and *Cd24a*[9,10] (delta) as well as lineage associated genes *Peg10*[11,12] (alpha) and *Irs4*[11] (epsilon) for CellRank (**a**) and Palantir (**b**). The x-axis is given by Palantir's pseudotime (Supplementary Fig. 4c). Expression values were imputed using MAGIC[13]. Green ticks indicate that methods correctly predicted lineage-specific gene regulation. CellRank and

Palantir give similar results because both methods were supplied with CellRank's terminal states. **c.** UMAP with cluster labels from ref.[2] **d.** Expression of the genes from (**a**) and (**b**) in the UMAP. **e.** CellRank's initial and terminal states, as in Fig. 2d. (T) denotes a terminal state, (I) denotes an initial state.

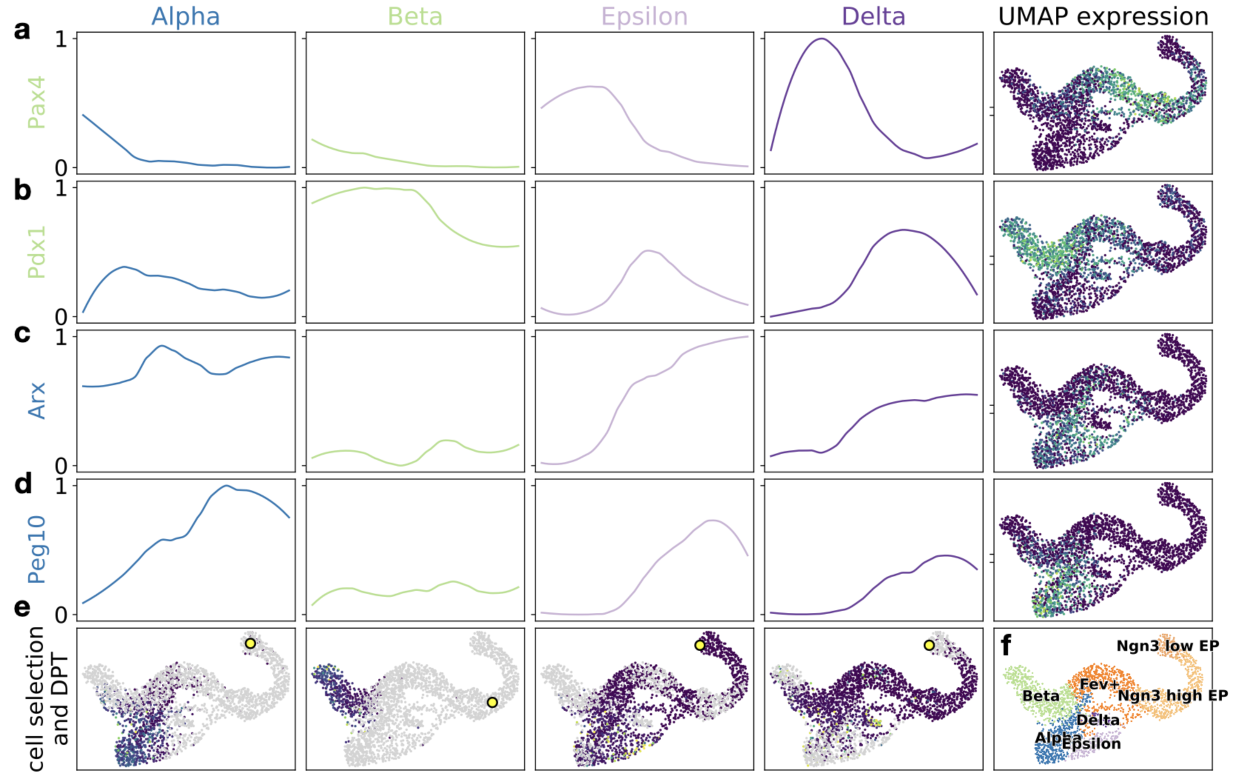

Suppl. Fig. 13 | FateID gene expression trends for alpha- and beta-fate associated genes

a-d. Gene expression trends for key regulators *Pax4*[4] and *Pdx1*[5–7] (beta), *Arx*[4] (alpha) as well as the lineage-associated gene *Peg10*[11,12] (alpha). Each gene is colored by its associated lineage. Expression trends computed using FateID towards the alpha, beta, epsilon and delta fates for these genes are shown in the first four columns. Normalised gene expression is plotted against indices of the pseudo-temporally ordered cells assigned to the given lineage. The line represents a local regression of z-transformed gene expression values (Supplementary Note 2). The last column shows gene expression values in the UMAP from low (blue) to high (yellow). **e.** Cells assigned to each lineage by FateID, colored by diffusion pseudotime[14] (DPT) which was used for gene-trend smoothing (Supplementary Note 2). The yellow dot denotes the root cell used for DPT computation in the corresponding lineage. Cells not assigned to a lineage are colored in grey. **f.** Cluster annotations from the original publication[2].

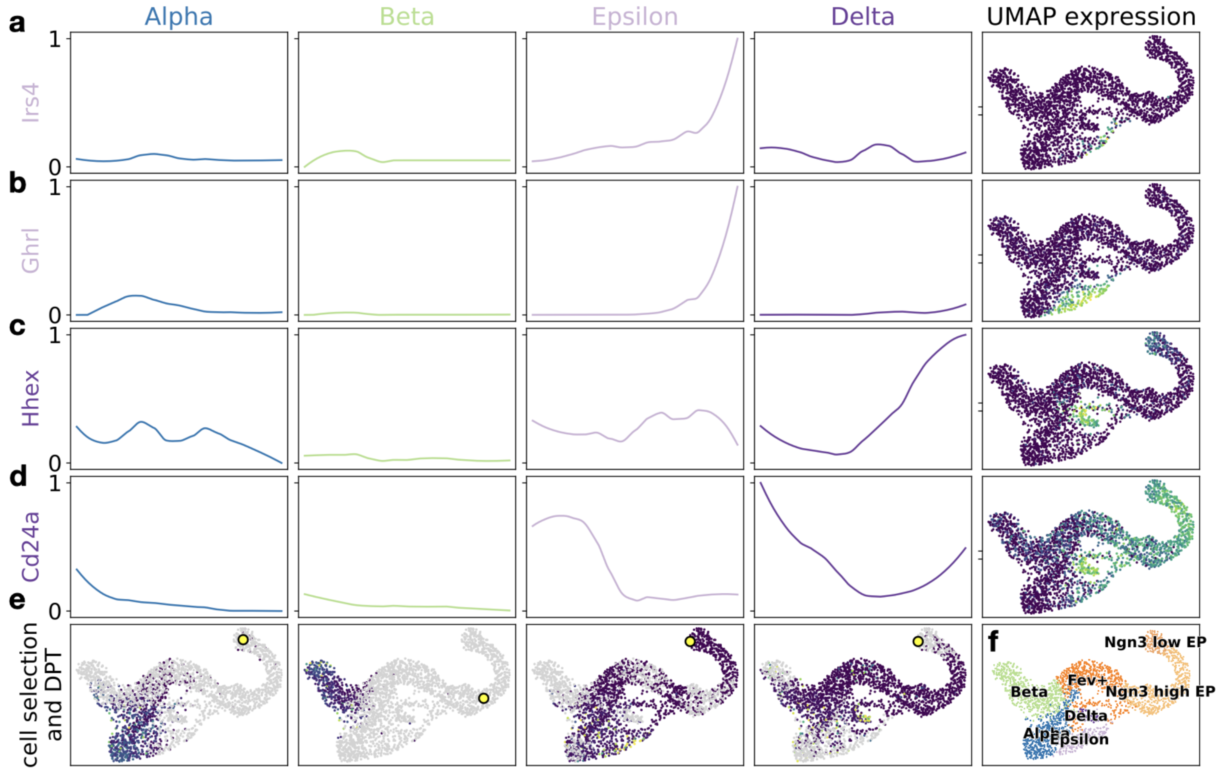

Suppl. Fig. 14 | FateID gene expression trends for epsilon- and delta-fate associated genes

a-d. Same analysis as in Supplementary Fig. 13, for the key lineage drivers *Ghrl*[4] (epsilon), *Hhex*[8] and *Cd24a*[9,10] (delta) as well as for the lineage associated gene *Irs4*[11] (epsilon). Panels **e** and **f** remain unchanged.

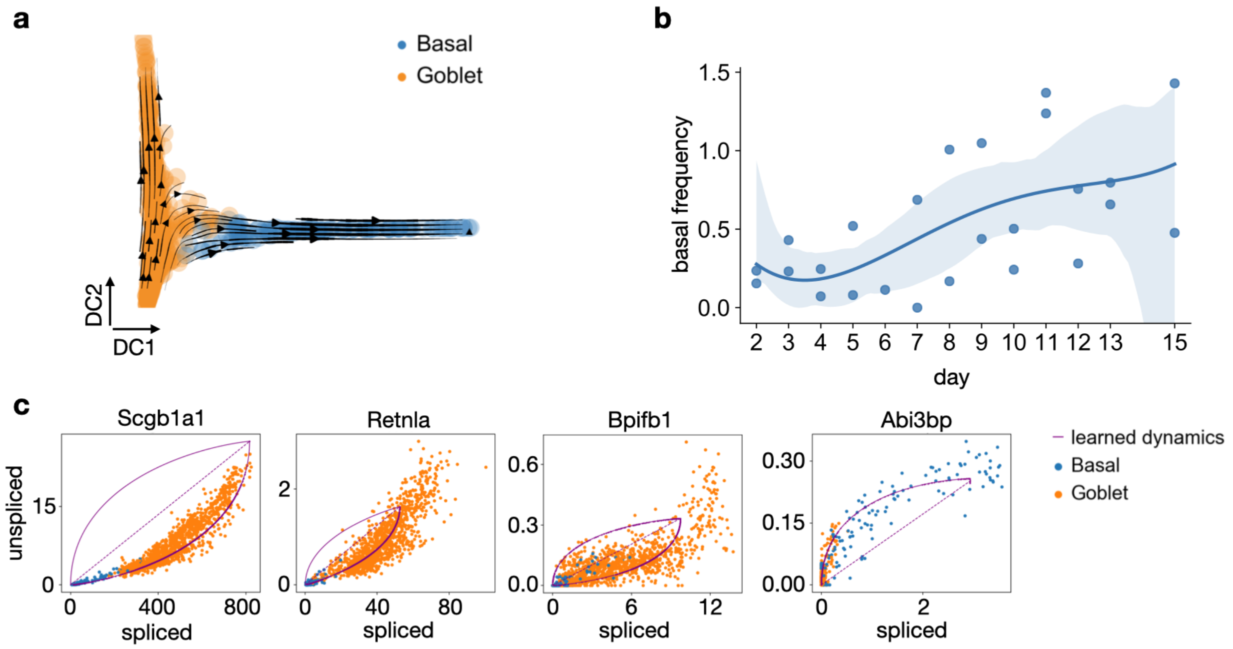

Suppl. Fig. 15 | Basal cell frequency increases over time and gene-wise velocities support dedifferentiation

a. Diffusion map computed on the subset of basal and goblet cells, showing scVelo computed velocities as streamlines. **b.** Proportion of basal cells per sample for each of the two samples available per time point. Blue line shows a 4th order polynomial regression fit, shaded regions are 95% confidence intervals computed through bootstrap sampling. **c.** Scatter plots of spliced vs. unspliced counts for *Scgb1a1, Retnla, Bpifb1* and *Abi3bp,* all of which are among the top 30 likelihood genes according to scVelo's dynamical model of splicing kinetics[15], colored by cell type. Purple line shows scVelo's fitted splicing dynamics which support the goblet -> basal direction for all 4 genes. Both *Scbg1a1*[16] as well as *Bpifb1*[17] are known markers for secretory/goblet cells and are downregulated in the transition. The top 100 likelihood genes further include known goblet cell markers *Muc5b* and *Muc5ac*[18], highlighting that velocities are driven by biologically meaningful genes (data now shown).

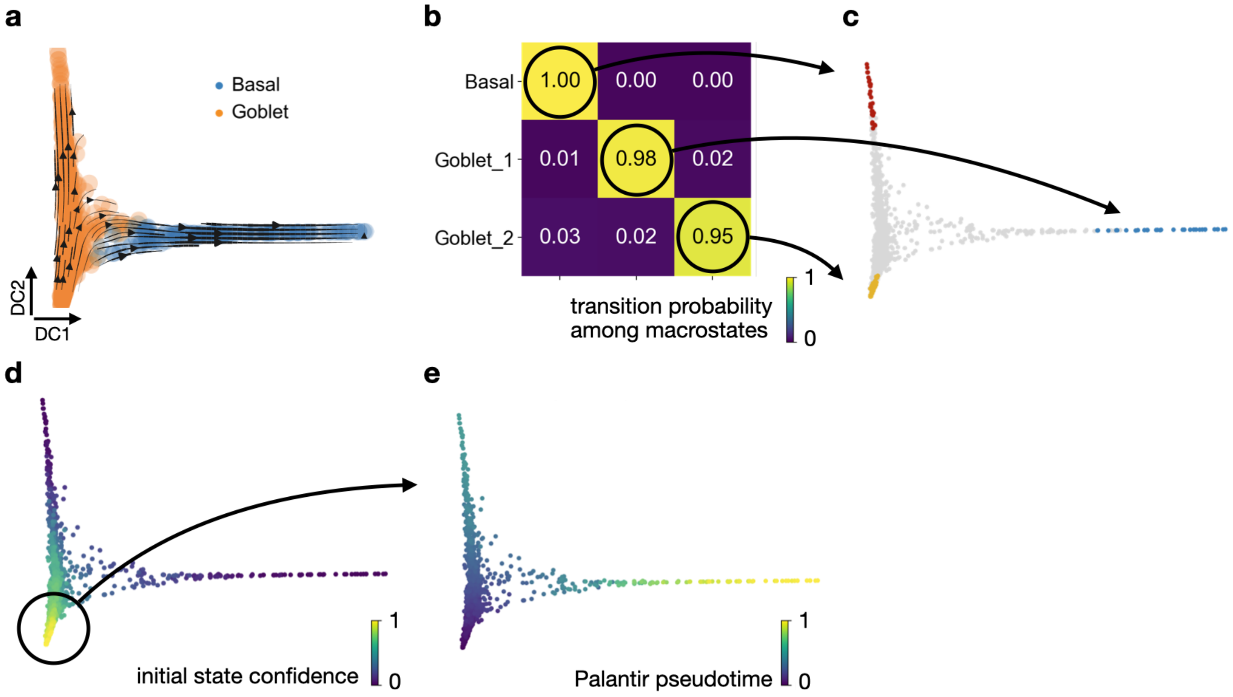

Suppl. Fig. 16 | Computing a pseudotime for the goblet to basal transitions

a. Diffusion map of a subset of the cells from the lung data of Fig. 6d labelled as "Goblet" and "Basal" in the original publication[19]. **b** Coarse-grained transition matrix, computed for three macrostates. The macrostate labelled as 'Goblet_2' was automatically detected as initial by CellRank because it had the smallest value in the CGSD. **c.** Showing the 30 cells most confidently assigned to their macrostate in the diffusion map. We kept the color for the basal state but created two new colors for the initial and terminal goblet states because they both overlap with the same transcriptomic goblet cluster and hence would both get the same color. **d.** Membership vector corresponding to the initial 'Goblet_2' state, here labelled as 'initial state confidence'. The cell which had the maximum value in the initial state confidence was used as initial cells to compute Palantir's pseudotime. **e.** Palantir pseudotime.

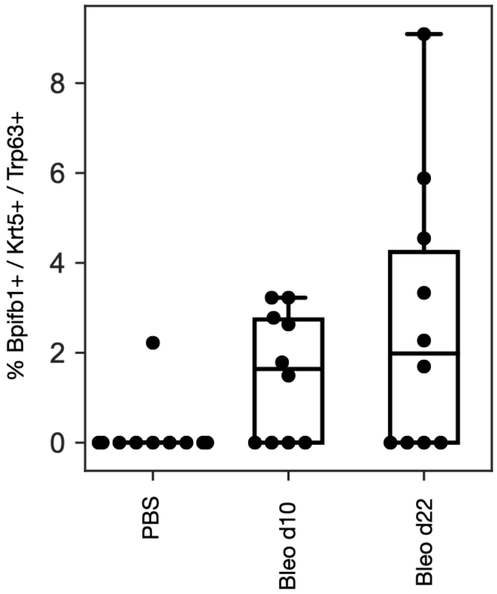

Suppl. Fig. 17 | Quantifying the abundance of triple positive cells

We quantify the abundance of cells positive for the goblet cell marker *Bpifb1* as well as the basal cell markers *Krt5* and *Trp63* in the three stages in control mice treated with PBS, ten days past bleomycin injury (Bleo d10), and 22 days past bleomycin injury (Bleo d22) in ten different intrapulmonary airway regions, derived from two independent biological replicates (n = 5 airway regions per mouse). We find triple positive cells in bleomycin-injured lungs and rarely in PBS treated control mice.

Supplementary tables

#cells (k)	CR (I/T) [μ]	CR (I/T) [σ]	CR (FP) [μ]	CR (FP) [σ]	P (FP) [μ]	P (FP) [σ]	S (FP) [μ]	S (FP) [σ]	F (FP) [μ]	F (FP) [σ]	V (I/T) [μ]	V (I/T) [σ]
**10**	2.8	0.9	1.5	2.2	27.3	3	6.5	0.3	160.5	8.8	525.5	5.9
**20**	5	0.1	4.2	0.8	102.5	10.4	11.9	0.4	531.1	31.7	2195.5	34.9
**30**	7.6	0.1	8.8	0.8	217.6	19.8	17.7	0.4	1029.6	57.8	5325.9	274
**40**	11.1	0.5	18.3	1.6	447.7	52.6	22.9	0.3	1840.8	89.5	9889.8	271.9
**50**	15	0.3	32.1	2.9	750.1	66.1	28.9	0.3	2690.5	117.5	NA	NA
**60**	18.8	2	35.9	6.2	1092.4	119.5	35	0.6	3772.8	147	NA	NA
**70**	21.8	0.5	48.5	1.8	1524.1	158.9	42.1	0.5	5151.4	217.9	NA	NA
**80**	26.2	1.8	65.7	2.5	2352.1	292.4	48.4	0.5	7414.3	454.8	NA	NA
**90**	29.7	1.3	96.9	11.5	3424.8	130.4	54.3	0.6	9752.7	380.9	NA	NA
**100**	32.5	0.9	124.8	4.1	4371.5	226.3	60.3	1.2	NA	NA	NA	NA

Suppl. Tab. 1 | Compute time benchmarks

Compute times for CellRank (CR), Palantir[3] (P), STEMNET[20] (S), FateID[21] (F) and velocyto[22] (V) for the 100k reprogramming dataset of ref.[23] to compute fate probabilities (FP) and/or initial and terminal states (I/T) (Supplementary Note 2). Values represent time in seconds. CellRank was the only method to compute all of initial states, terminal states and fate probabilities. We repeated each computation 10 times to compute mean **[μ]** and standard error on the mean **[σ]**. While FateID failed on 100k cells due to memory constraints, velocyto exceeded our time budget of 10k seconds on cell numbers exceeding 40k (indicated by “NA”).

#cells (k)	CR (I/T) [μ]	CR (I/T) [σ]	CR (FP) [μ]	CR (FP) [σ]	P (FP) [μ]	P (FP) [σ]	S (FP) [μ]	S (FP) [σ]	F (FP) [μ]	F (FP) [σ]	V (I/T) [μ]	V (I/T) [σ]
**10**	7.04	0.15	9.9	0.09	9.74	0.06	0.23	0	2.55	0	16.24	0.05
**20**	10.59	0.01	12.64	0.1	14.15	0.09	0.46	0	9.81	0.62	57.58	0.04
**30**	13.81	0.04	15.53	0.08	20.02	0.19	0.68	0	18.5	0.01	118.81	0.03
**40**	17.18	0.02	18.26	0.07	27.43	0.34	0.91	0	34.88	0.12	206.32	1.13
**50**	20.39	0.04	21.02	0.1	36.89	0.28	1.14	0	52.72	0.35	NA	NA
**60**	23.61	0.03	23.83	0.09	47.38	0.55	1.36	0	73.43	1.85	NA	NA
**70**	26.88	0.02	26.68	0.06	53.24	0.81	1.59	0	98.92	1.28	NA	NA
**80**	30.14	0.02	29.63	0.13	66.28	1.2	1.81	0	124.65	1.96	NA	NA
**90**	33.41	0.03	32.55	0.15	81.19	1.08	2.04	0	155.1	3.07	NA	NA
**100**	36.69	0.03	35.52	0.14	98.26	0.83	2.26	0	NA	NA	NA	NA

Suppl. Tab. 2 | Peak memory usage benchmarks

Peak memory usage for CellRank (CR), Palantir[3] (P), STEMNET[20] (S), FateID[21] (F) and velocyto[22] (V) for the 100k reprogramming dataset of ref.[23] to compute fate probabilities (FP) and/or initial and terminal states (I/T) (Supplementary Note 2). Values represent peak memory usage in GiB. CellRank was the only method to compute all of initial states, terminal states and fate probabilities. We repeated each computation 10 times to compute mean **[μ]** and standard error on the mean **[σ]**. While FateID failed on 100k cells due to memory constraints, velocyto exceeded our time budget of 10k seconds on cell numbers exceeding 40k (indicated by "NA"). CellRank and Palantir make use of parallelisation to speed up their computations which increases peak memory usage (Supplementary Note 2). We report decreased peak memory usage on 100k cells using a single core for Palantir and CellRank in Supplementary Tab. 3.

#cells (k)	CR (I/T) [μ]	CR (I/T) [σ]	CR (FP) [μ]	CR (FP) [σ]	P (FP) [μ]	P (FP) [σ]
**100**	12.8	0.02	14.22	0.05	82.5	4.35

Suppl. Tab. 3 | Peak memory usage on a single core

Running the memory benchmark of Supplementary Tab. 2 on a single core on 100k cells for the methods which make use of parallelisation, CellRank (CR) and Palantir[3] (P) to compute fate probabilities (FP) and/or initial and terminal states (I/T). Values represent peak memory usage in GiB.We repeated each computation 10 times to compute mean **[μ]** and standard error on the mean **[σ].**

Supplementary Figure and Table References

1. Wolf, F. A. *et al.* PAGA: graph abstraction reconciles clustering with trajectory inference through a topology preserving map of single cells. *Genome Biol.* **20**, 59 (2019).
2. Bastidas-Ponce, A. *et al.* Comprehensive single cell mRNA profiling reveals a detailed roadmap for pancreatic endocrinogenesis. *Development* **146**, (2019).
3. Setty, M. *et al.* Characterization of cell fate probabilities in single-cell data with Palantir. *Nat. Biotechnol.* **37**, 451–460 (2019).
4. Bastidas-Ponce, A., Scheibner, K., Lickert, H. & Bakhti, M. Cellular and molecular mechanisms coordinating pancreas development. *Development* **144**, 2873–2888 (2017).
5. Bastidas-Ponce, A. *et al.* Foxa2 and Pdx1 cooperatively regulate postnatal maturation of pancreatic β-cells. *Mol Metab* **6**, 524–534 (2017).
6. Stoffers, D. A., Zinkin, N. T., Stanojevic, V., Clarke, W. L. & Habener, J. F. Pancreatic agenesis attributable to a single nucleotide deletion in the human IPF1 gene coding sequence. *Nat. Genet.* **15**, 106–110 (1997).
7. Jonsson, J., Carlsson, L., Edlund, T. & Edlund, H. Insulin-promoter-factor 1 is required for pancreas development in mice. *Nature* **371**, 606–609 (1994).
8. Zhang, J., McKenna, L. B., Bogue, C. W. & Kaestner, K. H. The diabetes gene Hhex maintains δ-cell differentiation and islet function. *Genes Dev.* **28**, 829–834 (2014).
9. Berthault, C., Staels, W. & Scharfmann, R. Purification of pancreatic endocrine subsets reveals increased iron metabolism in beta cells. *Mol Metab* **42**, 101060 (2020).
10. Cram, D. S., McIntosh, A., Oxbrow, L., Johnston, A. M. & DeAizpurua, H. J. Differential mRNA display analysis of two related but functionally distinct rat insulinoma (RIN) cell lines: identification of CD24 and its expression in the developing pancreas. *Differentiation* **64**, 237–246 (1999).
11. Krentz, N. A. J. *et al.* Single-Cell Transcriptome Profiling of Mouse and hESC-Derived

Pancreatic Progenitors. *Stem Cell Reports* **11**, 1551–1564 (2018).

12. Byrnes, L. E. *et al.* Lineage dynamics of murine pancreatic development at single-cell resolution. *Nat. Commun.* **9**, 3922 (2018).
13. van Dijk, D. *et al.* Recovering Gene Interactions from Single-Cell Data Using Data Diffusion. *Cell* **174**, 716–729.e27 (2018).
14. Haghverdi, L., Büttner, M., Wolf, F. A., Buettner, F. & Theis, F. J. Diffusion pseudotime robustly reconstructs lineage branching. *Nat. Methods* **13**, 845–848 (2016).
15. Bergen, V., Lange, M., Peidli, S., Wolf, F. A. & Theis, F. J. Generalizing RNA velocity to transient cell states through dynamical modeling. *Nat. Biotechnol.* (2020) doi:10.1038/s41587-020-0591-3.
16. Rawlins, E. L. & Perl, A.-K. The a'MAZE'ing world of lung-specific transgenic mice. *Am. J. Respir. Cell Mol. Biol.* **46**, 269–282 (2012).
17. Musa, M. *et al.* Differential localisation of BPIFA1 (SPLUNC1) and BPIFB1 (LPLUNC1) in the nasal and oral cavities of mice. *Cell Tissue Res.* **350**, 455–464 (2012).
18. Portal, C. *et al.* In vivo imaging of the Muc5b gel-forming mucin. *Sci. Rep.* **7**, 44591 (2017).
19. Strunz, M. *et al.* Alveolar regeneration through a Krt8 transitional stem cell state that persists in human lung fibrosis. *Nature Communications* vol. 11 (2020).
20. Velten, L. *et al.* Human haematopoietic stem cell lineage commitment is a continuous process. *Nat. Cell Biol.* **19**, 271–281 (2017).
21. Herman, J. S., Sagar & Grün, D. FateID infers cell fate bias in multipotent progenitors from single-cell RNA-seq data. *Nat. Methods* **15**, 379–386 (2018).
22. La Manno, G. *et al.* RNA velocity of single cells. *Nature* **560**, 494–498 (2018).
23. Biddy, B. A. *et al.* Single-cell mapping of lineage and identity in direct reprogramming. *Nature* **564**, 219–224 (2018).

September 19, 2021

CellRank - Supplementary Notes

Marius Lange[1,2], Volker Bergen[1,2], Michal Klein[1], Manu Setty[3], Bernhard Reuter[4,5], Mostafa Bakhti[6,7], Heiko Lickert[6,7], Meshal Ansari[1,8], Janine Schniering[8], Herbert B. Schiller[8], Dana Pe'er[3*], Fabian J. Theis[1,2,9*]

1 Institute of Computational Biology, Helmholtz Center Munich, Germany.
2 Department of Mathematics, TU Munich, Germany.
3 Program for Computational and Systems Biology, Sloan Kettering Institute, Memorial Sloan Kettering Cancer Center, New York, NY, USA.
4 Department of Computer Science, University of Tübingen, Germany.
5 Zuse Institute Berlin (ZIB), Takustr. 7, 14195 Berlin, Germany.
6 Institute of Diabetes and Regeneration Research, Helmholtz Center Munich, Germany.
7 German Center for Diabetes Research (DZD), Neuherberg, Germany.
8 Comprehensive Pneumology Center (CPC) / Institute of Lung Biology and Disease (ILBD), Helmholtz Zentrum München, Member of the German Center for Lung Research (DZL), Munich, Germany
9 TUM School of Life Sciences Weihenstephan, Technical University of Munich, Germany.
*Corresponding authors: fabian.theis@helmholtz-muenchen.de and peerd@mskcc.org

Contents

1 Computing a directed PAGA graph 2
2 Methods comparison 2
2.1 Algorithm overview 2
2.2 Comparing initial and terminal states 4
2.3 Comparing fate probabilities 4
2.4 Comparing gene expression trends 5
2.5 Comparing runtime 5
2.6 Comparing peak memory usage 6

Supplementary Notes

1 Computing a directed PAGA graph

Partition-based graph abstraction (PAGA)[1] provides an interpretable, graph-like connectivity map of the data manifold. It is obtained by associating a node with each manifold partition (e.g. cell type) and connecting each node by weighted edges that represent a statistical measure of connectivity between the partitions. The model considers groups/nodes as connected if their number of inter-edges exceeds what would have been expected under random assignment. The connection strength can be interpreted as confidence in the presence of an actual connection and allows discarding spurious, noise-related connections.

Here, we extend PAGA by directing the edges as to reflect the RNA velocity vector field rather than transcriptome similarity. The connectivity strengths are defined based on the velocity graph (Online Methods). Inter-edges are considered whose correlation passes a certain threshold (default: 0.1). The number of inter-edges are then tested against random assignment for significance.

To further constrain the single cell graph, we compute a gene-shared latent time using scVelo[2]. In short, this aggregates the per-gene time assignments computed in scVelo's dynamical model to a global scale which faithfully approximates a single-cells internal clock. Once we have computed the initial states using CellRank, we can use these as a prior for latent time. Latent time, initial and terminal states can in turn be used as a prior to regularize the directed graph. At single-cell level, we use latent time as a constraint to prune the cell-to cell transition edges to those that match the ordering of cells given by latent time. For the initial and terminal states, the edges are further constrained to only retain those cell-to-cell transitions that constitute outgoing flows for cells in initial cellular populations, and to incoming flows for cells in terminal populations.

Finally, a minimum spanning is constructed for the directed abstracted graph. It is obtained by pruning node-to-node edges such that only the most confident path from one node to another is retained. If there are multiple paths to reach a particular node, only the path with the highest confidence is kept.

2 Methods comparison

We compared CellRank with the following similarity-based tools that compute probabilistic fate assignments on the single cell level: Palantir[3], FateID[4] and STEMNET[5]. Additionally, we compared to the velocity-based Velocyto[6] algorithm. We compared these methods in terms of the identification of initial and terminal states, fate probabilities, gene expression trends, run-time and memory usage.

2.1 Algorithm overview

The Palantir algorithm Palantir[3] computes a KNN graph in the space of diffusion components and uses this graph to compute a pseudotime via iteratively updating shortest path distances from a set of waypoints. Palantir required us to provide a number of *waypoint cells* - essentially a smaller number of cells that the system is reduced to in order to make it computationally feasible. We set this number to 1200 cells for the pancreas data and to 15% of the total cell number of the runtime and memory benchmarks on the reprogramming dataset[7] below. For pseudotime computation, an initial cells needs to be supplied by the user. The pseudotime is used to direct edges in the KNN graph by removing edges that point from later cells to earlier cells in pseudotime. The stationary distribution of the resulting directed transition matrix is combined with extrema in the diffusion components to identify terminal cells. Absorption probabilities towards the terminal cells serve as fate probabilities. Gene expression trends are computed similarly to CellRank, by fitting GAMs in pseudotime where each cell contributes to each lineage according to its fate probabilities.

The FateID algorithm FateID[4] either requires the user to provide terminal populations directly or through a set of marker genes. Terminal populations are used to train a random forest classifier. The classifier is applied to a set of cells in the neighborhood of each terminal cluster where it predicts the likely fate of these cells. The training set is iteratively expanded and the Random forest is re-trained on expanding populations, thus moving from the committed populations backward in time, classifying the fate of increasingly earlier cells. Two key parameters here are the size of the training and test sets used for the Random forest classifier, which we set to 1% of the data in all benchmarks. Gene expression trends are computed by selecting a (discrete) set of cells which pass a certain threshold for fate bias towards a specific terminal population. A principal curve is fit to these cells in a low dimensional embedding and pseudotime is assigned via projection onto this curve. Alternatively, the authors recommend to compute diffusion pseudotime[8] (DPT) on the set of cells selected for a particular lineage. Gene expression values are then normalised and a local regression (LOESS) is performed to obtain mean trends. In contrast to CellRank and Palantir, this approach does not provide confidence intervals for the expression trends, it is dependent on low dimensional embeddings (principal curve fit) and it discreetly assigns cells to lineages, thereby ignoring the gradual nature of fate commitment when visualizing gene expression trends. Since different cells are selected for different lineages, the computed pseudo-temporal orderings are incompatible and gene trends along different lineages cannot be visualized jointly.

The STEMNET algorithm STEMNET[5] requires the user to provide the terminal populations directly as input to the algorithm. It then trains an elastic-net regularized generalized linear model on the terminal populations to predict state membership. This first step serves as feature selection - it selects a set of genes which are specific to their terminal populations. In the next step, the classifier uses expression of these genes to predict fate bias for the remaining, transient cells. STEMNET uses the computed fate probabilities to place cells on a simplex in 2 dimensions as a dimensionality reduction method. It does not offer a method to visualize gene expression trends.

The Velocyto algorithm Velocyto[6] was the original algorithm to compute RNA velocity based on ratios of spliced to unspliced counts by solving a system of two linear ordinary differential equations (ODEs) per gene under a steady-state assumption. This is equivalent to scVelo's deterministic mode of computing velocities[2]. Besides the computation of velocities, part of the velocyto software package enables the user to compute initial and terminal states. The first step in this computation is the construction of a directed cell-cell transition matrix based on transcriptomic displacements and velocity vectors, in a similar vain to what we have described in the Online Methods. However, we emphasize the following critical differences to CellRank's transition matrix construction:

- The KNN graph, obtained through calling velocyto's `estimate_transition_prob` method, is constructed in 2D tSNE embedding space, ignoring much of the high dimensional variation present in single-cell dynamics. In CellRank, in contrast, we construct the KNN graph in 30-dimensional principal component space, making sure that we capture all relevant biological variation.
- The KNN graph does not enforce velocity vector alignment with the local manifold structure in the same sense as CellRank, because the local neighborhood considered by velocyto is too large. The dentate gyrus is the only dataset where the authors apply their method to find initial and terminal states, and they consider 4000 nearest neighbors[6] out of 18,213 total cells in this dataset, corresponding to 22% of the data - an extremely large neighborhood. These large, blunt neighborhoods are almost certain to miss the fine structure of the phenotypic manifold, including rare populations and transitional populations. In contrast, CellRank considers neighborhoods of only 30 nearest neighbors by default. This is important to reduce noise by forcing velocity vectors to align with the local structure of the phenotypic manifold.

- The final transition matrix, obtained by calling velocyto's `prepare_markov` method, is constructed by subsampling cells to evenly cover the 2D tSNE embedding space. The authors subsample the original 18,213 cells of the dentate gyrus to 2,625 cells, arguing that this helps to reduce density-driven effects. However, relying on the 2D data representation carries a large risk of missing important variation that drives complex single-cell dynamics, leading in particular to the loss of rare and transitional cell populations. CellRank does not subsample, but rather constructs the transition matrix on all cells in the data, ensuring that we capture all variation needed to describe the biological process. To reduce the complexity of the data and to extract the key dynamics, we coarse-grain the large transition matrix using principled mathematical tools.
- Velocyto does not propagate velocity uncertainty. This feature is key to CellRank's success as it enables us to handle situations with noisy, uncertain velocity information

Once the transition matrix has been constructed, velocyto initialises a uniform distribution over all cells and propagates it forward and backward by repeatedly multiplying with the (transposed) transition matrix to obtain the terminal and initial states, respectively. We note that this procedure is equivalent to computing the stationary distribution of the Markov chain (under some mild conditions on the Markov chain), which can be done much more efficiently by considering the normalized left eigenvector corresponding to eigenvalue 1 (Online Methods). This computationally more efficient variant of the original velocyto approach for finding initial and terminal states is implemented in CellRank as well. However, note that this procedure does not separate out individual initial/terminal states - it only gives a probability for each cell to be initial/terminal, but it does not say anything about which cell belongs to which initial/terminal state. That is why we extended this approach in CellRank through GPCCA, enabling us to find multiple initial/terminal states and an assignment of cells to these states. Further, velocyto does not compute fate probabilities.

2.2 Comparing initial and terminal states

While STEMNET and FateID do not provide any automatic means to identify initial and terminal states, Palantir can automatically compute terminal states. Velocyto cannot identify individual initial and terminal states but it can compute a single distribution over all initial and terminal states, respectively. We run Palantir with default parameters, filtering to 1500 highly variable genes and using enough principal components to retain 85% of variance in the data (403 PCs). We also run Velocyto with mostly default parameters, following the analysis of their dentate gyrus data which is available from the Velocyto notebook repository[6]. We varied parameters to accommodate for the smaller cell number in our data (approx. 2.5k) compared to the cell number in the dentate gyrus data (approx. 18k). We filtered to 3000 highly variable genes and used a an elbow heuristic employed by Velocyto to select 21 principal components. We imputed spliced and unsliced counts using a KNN graph with 100 neighbors. We computed cosine correlations using a KNN graph with larger neighborhood size of 200. We subsampled cells to evenly cover the embedding which left us with 1,994 out of the original 2,531 cells. We run forwards/backwards diffusion on the Markov chain for 2500 steps starting from a uniform initial distribution.

2.3 Comparing fate probabilities

In order to enable a fair comparison across methods, we supplied Palantir, FateID and STEMNET with CellRank's identified terminal states and compared predicted fate probabilities. Velocyto does not provide fate probabilities. Methods differed in the format they require terminal state information to be passed: for Palantir, we passed individual cells, i.e. 4 cells, one for each of the three terminal states and one initial cell from the initial state. For STEMNET, we passed populations of cells defined through the underlying transcriptomic clusters. For FateID, we passed marker genes to identify the terminal populations. For each terminal state, we passed its corresponding hormone-

production associated gene, i.e. *Ins1* for beta, *Gcg* for alpha, *Ghrl* for epsilon and *Sst* for delta[9]. We checked whether methods correctly predicted beta to be the dominant fate among early cells by computing the average fate prediction among Ngn3 high EP cells.

2.4 Comparing gene expression trends

To visualize gene expression trends, we used the functionalities that each method provided. STEMNET and Velocyto do not provide options to compute gene expression trends. For CellRank, we visualized gene expression trends as described in the Online Methods. For Palantir, we used default parameters. For FateID, it was difficult to find a good threshold value to assign cells to lineages. If this value is too high, then early cells in the trajectory are not selected and the terminal states are isolated. If this value is too low, then for a subset of the lineages, very unlikely cells are assigned and the trends are very unspecific. The default value is 0.25, which was too high in our case. We decided to set the threshold at 0.15, which was a compromise between trying to have early cells in every lineage and making sure that irrelevant cells are not assigned. We computed DPT[8] on the set of the selected cells, as recommended in the original publication. To identify a root cell for each lineage, we first computed DPT on the entire data-set, then subsetted to a lineage-specific set of cells and picked the cell with the earliest original DPT value as the root cell for the second DPT computation. We visualized expression trends for the key lineage drivers *Pax4*[10] and *Pdx1*[11–13] (beta), *Arx*[10] (alpha), *Ghrl*[9] (epsilon) and *Hhex*[14] and *Cd24a*[15,16] (delta) as well as the lineage accociated genes *Peg10*[17,18] (alpha) and *Irs4*[18] (epsilon). In Fig. 5c, we checked whether methods correctly predicted upregulation of *Pdx1* along the beta fate.

2.5 Comparing runtime

We compared run-time of the five methods applied to a scRNA-seq dataset comprising 100k cells undergoing reprogramming from mouse embryonic fibroblasts to induced endoderm progenitor cells[7]. We randomly subsampled the data-set to obtain 10 data-sets of increasing size, starting from 10k cells in steps of 10k until 100k cells. For each sub-sampled dataset, we applied each method 10 times and computed the mean run-time as well as the standard error on the mean.

Overview of tasks CellRank was the only method to compute both initial and terminal states as well as fate probabilities. The other methods could compute varying subsets of these properties. Therefore, the actual task we benchmarked differed slightly across methods. In the following list, (I) denotes the computation of initial states, (T) denotes the computation of terminal states and (F) denotes the computation of fate probabilities.

- CellRank: (I + T), F
- Palantir: F
- FateID: F
- STEMNET: F
- Velocyto: (I + T)

For CellRank, we separately recorded the time it took to compute initial and terminal states as well as fate probabilities.

Initial and terminal states: what we benchmarked For initial and terminal states in CellRank, we included in this benchmark the entire workflow from computing the transition matrix via decomposing it into macrostates to identifying the initial/terminal states among the macrostates. For Velocyto to compute initial/terminal states, we benchmarked the `estimate_transition_probability`

method (computes cosine correlations), the `calculate_embedding_shift` method (transforms cosine correlation into probabilities), the `prepare_markov` method (adds Gaussian noise to the transition probabilities) and the `run_markov` method (runs the Markov chain forwards/backwards in time).

Fate probabilities: CellRank used to provide terminal state information We used CellRank to compute 3 terminal states and we supplied these to Palantir, FateID and STEMNET for the computation of fate probabilities to ensure that the number of terminal states is consistent across methods. Methods differed in the format they require terminal state information to be passed: for Palantir, we passed individual cells, i.e. three terminal cells and one initial cell (taken from the earliest time point of the reprogramming data). For STEMNET, we passed a set of cells for each terminal state by choosing the cells which have been most confidently assigned to each terminal state by CellRank. For each terminal state, we passed a number of cells that was equal to 1% of the total cell number. FateID requires marker genes to identify the terminal populations, so we computed the top 3 lineage drivers per CellRank-identified terminal state and passed these.

Fate probabilities: what we benchmarked For fate probabilities, we benchmarked the `compute_absorption_probabilities()` method (CellRank), the `run_palantir()` function (Palantir), the `fateBias()` function (FateID) and the `runSTEMNET()` function (STEMNET).

Computational resources used Comparisons were run on an Intel(R) Xeon(R) Gold 6126 CPU @ 2.60GHz and 32 cores. Each job was allocated at least 90 GiB RAM and we recorded the actual peak memory usage (see below). FateID did not finish on 100k cells because of a memory error due to densification of a large matrix.

2.6 Comparing peak memory usage

The setup was identical to the setup for the runtime comparison above, only that we recorded peak memory usage of each method (Supplementary Tab. 2). For the Python-based methods CellRank, Palantir and velocyto, we used the `memory-profiler`[19] package whereas for the R-based packages STEMNET and FateID, we used the peakRAM[20] profiler. CellRank and Palantir efficiently parallelize their computations across several cores which increases their peak memory consumption. We repeated our evaluation for these two methods on 100k cells using just a single core to estimate the size of this effect (Supplementary Tab. 3).

References

[1] F. Alexander Wolf, et al. PAGA: graph abstraction reconciles clustering with trajectory inference through a topology preserving map of single cells. *Genome Biology*, 20(1):59, March 2019. ISSN 1474-760X. doi: 10.1186/s13059-019-1663-x. URL https://doi.org/10.1186/s13059-019-1663-x.

[2] Volker Bergen, et al. Generalizing RNA velocity to transient cell states through dynamical modeling. *Nature Biotechnology*, pages 1–7, August 2020. ISSN 1546-1696. doi: 10.1038/s41587-020-0591-3. URL https://www.nature.com/articles/s41587-020-0591-3. Publisher: Nature Publishing Group.

[3] Manu Setty, et al. Characterization of cell fate probabilities in single-cell data with Palantir. *Nature Biotechnology*, 37(4):451, April 2019. ISSN 1546-1696. doi: 10.1038/s41587-019-0068-4. URL https://www.nature.com/articles/s41587-019-0068-4.

[4] Josip S. Herman, et al. FateID infers cell fate bias in multipotent progenitors from single-cell RNA-seq data. *Nature Methods*, 15(5):379–386, May 2018. ISSN 1548-7105. doi: 10.1038/nmeth.4662. URL https://www.nature.com/articles/nmeth.4662. Number: 5 Publisher: Nature Publishing Group.

[5] Lars Velten, et al. Human haematopoietic stem cell lineage commitment is a continuous process. *Nature Cell Biology*, 19(4):271–281, April 2017. ISSN 1476-4679. doi: 10.1038/ncb3493. URL https://www.nature.com/articles/ncb3493. Number: 4 Publisher: Nature Publishing Group.

[6] Gioele La Manno, et al. RNA velocity of single cells. *Nature*, page 1, August 2018. ISSN 1476-4687. doi: 10.1038/s41586-018-0414-6. URL https://www.nature.com/articles/s41586-018-0414-6.

[7] Brent A. Biddy, et al. Single-cell mapping of lineage and identity in direct reprogramming. *Nature*, 564(7735):219, December 2018. ISSN 1476-4687. doi: 10.1038/s41586-018-0744-4. URL https://www.nature.com/articles/s41586-018-0744-4.

[8] Laleh Haghverdi, et al. Diffusion pseudotime robustly reconstructs lineage branching. *Nature Methods*, 13(10):845–848, October 2016. ISSN 1548-7105. doi: 10.1038/nmeth.3971. URL https://www.nature.com/articles/nmeth.3971.

[9] Aimée Bastidas-Ponce, et al. Cellular and molecular mechanisms coordinating pancreas development. *Development*, 144(16):2873–2888, August 2017. ISSN 0950-1991, 1477-9129. doi: 10.1242/dev.140756. URL https://dev.biologists.org/content/144/16/2873. Publisher: Oxford University Press for The Company of Biologists Limited Section: REVIEW.

[10] Patrick Collombat, et al. Opposing actions of Arx and Pax4 in endocrine pancreas development. *Genes & Development*, 17(20):2591–2603, October 2003. ISSN 0890-9369, 1549-5477. doi: 10.1101/gad.269003. URL http://genesdev.cshlp.org/content/17/20/2591. Company: Cold Spring Harbor Laboratory Press Distributor: Cold Spring Harbor Laboratory Press Institution: Cold Spring Harbor Laboratory Press Label: Cold Spring Harbor Laboratory Press Publisher: Cold Spring Harbor Lab.

[11] Aimée Bastidas-Ponce, et al. Foxa2 and Pdx1 cooperatively regulate postnatal maturation of pancreatic -cells. *Molecular Metabolism*, 6(6):524–534, June 2017. ISSN 2212-8778. doi: 10.1016/j.molmet.2017.03.007. URL http://www.sciencedirect.com/science/article/pii/S2212877817300510.

[12] J. Jonsson, et al. Insulin-promoter-factor 1 is required for pancreas development in mice. *Nature*, 371 (6498):606–609, 1994. doi: 10.1038/371606a0.

[13] D.A. Stoffers, et al. Pancreatic agenesis attributable to a single nucleotide deletion in the human IPF1 gene coding sequence. *Nature Genetics*, 15(1):106–110, 1997. doi: 10.1038/ng0197-106.

[14] Jia Zhang, et al. The diabetes gene Hhex maintains -cell differentiation and islet function. *Genes & Development*, 28(8):829–834, April 2014. ISSN 0890-9369. doi: 10.1101/gad.235499.113. URL https://www.ncbi.nlm.nih.gov/pmc/articles/PMC4003275/.

[15] C. Berthault, et al. Purification of pancreatic endocrine subsets reveals increased iron metabolism in beta-cells. *Molecular Metabolism*, page 101060, August 2020. ISSN 22128778. doi: 10.1016/j.molmet.2020.101060. URL https://linkinghub.elsevier.com/retrieve/pii/S2212877820301344.

[16] David S. Cram, et al. Differential mRNA display analysis of two related but functionally distinct rat insulinoma (RIN) cell lines: identification of CD24 and its expression in the developing pancreas.

Differentiation, 64(4):237–246, May 1999. ISSN 03014681. doi: 10.1046/j.1432-0436.1999.6440237.x. URL https://linkinghub.elsevier.com/retrieve/pii/S0301468109603984.

[17] Lauren E. Byrnes, et al. Lineage dynamics of murine pancreatic development at single-cell resolution. *Nature Communications*, 9(1):3922, September 2018. ISSN 2041-1723. doi: 10.1038/s41467-018-06176-3. URL https://www.nature.com/articles/s41467-018-06176-3. Number: 1 Publisher: Nature Publishing Group.

[18] Nicole A. J. Krentz, et al. Single-Cell Transcriptome Profiling of Mouse and hESC-Derived Pancreatic Progenitors. *Stem Cell Reports*, 11(6):1551–1564, December 2018. ISSN 2213-6711. doi: 10.1016/j.stemcr.2018.11.008. URL https://www.sciencedirect.com/science/article/pii/S2213671118304764.

[19] Fabian Pedregosa. memory-profiler: A module for monitoring memory usage of a python program, 2012. URL https://github.com/pythonprofilers/memory_profiler.

[20] Thomas Quinn. peakRAM: Monitor the Total and Peak RAM Used by an Expression or Function, January 2017. URL https://CRAN.R-project.org/package=peakRAM.
