# Supplementary material for: CellRank for directed single-cell fate mapping
Source: Nat Methods. 2022 Jan 13;19(2):159–70. doi: 10.1038/s41592-021-01346-6 (PMC8828480; doi:10.1038/s41592-021-01346-6)
Supplement: Supplementary file 2 — Reporting Summary [file 41592_2021_1346_MOESM2_ESM.pdf]

nature research

Corresponding author(s): Fabian Theis, Dana Pe'er

Last updated by author(s): Oct 5, 2021

Reporting Summary

Nature Research wishes to improve the reproducibility of the work that we publish. This form provides structure for consistency and transparency in reporting. For further information on Nature Research policies, see our Editorial Policies and the Editorial Policy Checklist.

Statistics

For all statistical analyses, confirm that the following items are present in the figure legend, table legend, main text, or Methods section.

n/a	Confirmed	
☐	☒	The exact sample size (*n*) for each experimental group/condition, given as a discrete number and unit of measurement
☐	☒	A statement on whether measurements were taken from distinct samples or whether the same sample was measured repeatedly
☐	☒	The statistical test(s) used AND whether they are one- or two-sided<br>*Only common tests should be described solely by name; describe more complex techniques in the Methods section.*
☐	☒	A description of all covariates tested
☐	☒	A description of any assumptions or corrections, such as tests of normality and adjustment for multiple comparisons
☐	☒	A full description of the statistical parameters including central tendency (e.g. means) or other basic estimates (e.g. regression coefficient) AND variation (e.g. standard deviation) or associated estimates of uncertainty (e.g. confidence intervals)
☐	☒	For null hypothesis testing, the test statistic (e.g. F, t, r) with confidence intervals, effect sizes, degrees of freedom and P value noted<br>*Give P values as exact values whenever suitable.*
☒	☐	For Bayesian analysis, information on the choice of priors and Markov chain Monte Carlo settings
☒	☐	For hierarchical and complex designs, identification of the appropriate level for tests and full reporting of outcomes
☐	☒	Estimates of effect sizes (e.g. Cohen's *d*, Pearson's *r*), indicating how they were calculated

Our web collection on statistics for biologists contains articles on many of the points above.

Software and code

Policy information about availability of computer code

Data collection	No software was used for data collection.
Data analysis	We used scVelo v0.2.4, scanpy v1.8.1 and cellrank v1.5.0. We share our software in an open-source package on github (https://cellrank.org), jupyter notebooks (jupyter_core v4.6.3) to reproduce our figures and analysis in a public github repository (https://github.com/theislab/cellrank_reproducibility).

For manuscripts utilizing custom algorithms or software that are central to the research but not yet described in published literature, software must be made available to editors and reviewers. We strongly encourage code deposition in a community repository (e.g. GitHub). See the Nature Research guidelines for submitting code & software for further information.

Data

Policy information about availability of data

All manuscripts must include a data availability statement. This statement should provide the following information, where applicable:

- Accession codes, unique identifiers, or web links for publicly available datasets
- A list of figures that have associated raw data
- A description of any restrictions on data availability

Raw published data for the pancreas, lung and reprogramming examples is available from the Gene Expression Omnibus (GEO) under accession codes GSE132188, GSE141259 and GSE99915, respectively. Processed data, including spliced and unspliced count abundances, is available from figshare under https://doi.org/10.6084/m9.figshare.c.5172299.

Field-specific reporting

Please select the one below that is the best fit for your research. If you are not sure, read the appropriate sections before making your selection.

☒ Life sciences ☐ Behavioural & social sciences ☐ Ecological, evolutionary & environmental sciences

For a reference copy of the document with all sections, see nature.com/documents/nr-reporting-summary-flat.pdf

Life sciences study design

All studies must disclose on these points even when the disclosure is negative.

Sample size	No sample size calculation was performed in this study. Sample sizes were based on previous experience that resulted in generation of statistically significant data.
Data exclusions	Pancreas dataset: subset to the late stages of endocrinogenesis to focus on the process of Alpha, Beta, Epsilon and Delta fate establishment. Lung data: subset to days 2-15 to ensure that sampling is dense enough to have overlapping populations across timepoints, required for RNA velocity. Reprogramming data: subset to late stages of reprogramming, equivalent to Fig. 3 of original publication. See Methods for full details.
Replication	For the experimental follow-up of the predicted dedifferentiation trajectory (Fig. 6), we assessed and quantified 10 intrapulmonary airway regions derived from two independent biological replicates, which gave similar findings.
Randomization	Mice were randomized according to different treatment and time point groups.
Blinding	Investigators were not blinded to group allocations due to unblinding by the treatment effect.

Reporting for specific materials, systems and methods

We require information from authors about some types of materials, experimental systems and methods used in many studies. Here, indicate whether each material, system or method listed is relevant to your study. If you are not sure if a list item applies to your research, read the appropriate section before selecting a response.

Materials & experimental systems

n/a	Involved in the study
☐	☒ Antibodies
☒	☐ Eukaryotic cell lines
☒	☐ Palaeontology and archaeology
☐	☒ Animals and other organisms
☒	☐ Human research participants
☒	☐ Clinical data
☒	☐ Dual use research of concern

Methods

n/a	Involved in the study
☒	☐ ChIP-seq
☒	☐ Flow cytometry
☒	☐ MRI-based neuroimaging

Antibodies

Antibodies used	Primary antibodies: rabbit anti-Bpifb1 (kindly provided and produced by C. Bingle, 1:500; Musa, M. et al. Differential localisation of BPIFA1 (SPLUNC1) and BPIFB1 (LPLUNC1) in the nasal and oral cavities of mice. Cell Tissue Res. 350, 455–464 (2012)), mouse anti-Trp63 (abcam, ab735, clone 4A4, 1:50) and chicken anti-Krt5 (BioLegend, Poly9059, 1:1,000). Secondary antibodies: Goat anti-rabbit Alexa Fluor® 488 (Invitrogen, A11008, 1:250), Goat anti-chicken Alexa Fluor® 568 (Invitrogen, A11041,1:250) and goat anti-mouse Alexa Fluor® 647 (Invitrogen, A21236, 1:250). Cell nuclei were visualized with 4',6-diamidino-2-phenylindole (DAPI).
Validation	All antibodies used have been validated by the manufacturers (cf. antibodies above) and by multiple citations for use in immunofluorescence stainings. For Bpifb1: Musa, M. et al., Cell Tissue Res. 350, 455–464 (2012) and Bingle, L. et al., Histochem. Cell Biol. 138, 749–758 (2012). For Trp63: Kumar, P. A. et al., Cell vol. 147 525–538 (2011), and Zuo, W. et al., Nature 517, 616–620 (2015), For Krt5: Weiner, A. I. et al. NPJ Regen Med 4, 17 (2019).

Animals and other organisms

Policy information about studies involving animals; ARRIVE guidelines recommended for reporting animal research

Laboratory animals	Pathogen-free eight to ten weeks old female C57BL/6J mice were purchased from Charles River Germany and maintained at the appropriate biosafety level at ambient temperature (20-24°C) and humidity (45-65%) with a 12 hour light cycle. Animals were allowed food and water ad libitum.

Wild animals	This study did not involve wild animals.
Field-collected samples	This study did not involve field-collected samples.
Ethics oversight	All animal experiments were performed in accordance with the governmental and international guidelines and ethical oversight by the local government for the administrative region of Upper Bavaria (Germany), registered under 55.2-1-54-2532-130-2014 and ROB-55.2-2532.Vet_02-16-208.

Note that full information on the approval of the study protocol must also be provided in the manuscript.
